# From Target Product Profiles (TPPs) to Target Specimen Profiles (TSPs): A New Concept in Infectious Disease Biobanking for Diagnostic Applications

**DOI:** 10.3390/diagnostics15121503

**Published:** 2025-06-13

**Authors:** Fay Betsou, Warren Fransman, Patrick Lammie

**Affiliations:** 1 FIND (Foundation for Innovative New Diagnostics), 1218 Geneva, Switzerland; warren.fransman@finddx.org; 2 Task Force for Global Health, Decatur, GA 30030, USA; plammie@taskforce.org

**Keywords:** diagnostics, biospecimens, biobanking, infectious diseases

## Abstract

A target specimen profile (TSP) corresponds to the required characteristics of the specimen panels needed to demonstrate that a diagnostic kit meets the target product profile (TPP). TSPs can guide biobanks in the prospective collection of sample panels to support the development and validation of diagnostics.

## 1. Introduction

The development of new diagnostic tests continues to be a major need to support public health efforts designed to address a growing list of neglected diseases and emerging pathogens. Assessing the performance of these new tests requires robust validation protocols and well-characterized biological specimens. Herein, we describe a target specimen profile (TSP), a new tool to facilitate robust test validation.

For the sake of clarity in this article, the “development” of an in vitro diagnostic (IVD) refers to the initial identification or definition of promising diagnostic biomarkers, requiring relatively small numbers of clinically well-characterized biospecimens. The “prequalification assessment” (PQA) of an IVD device refers to checking the overall suitability for approval or use by the World Health Organization (WHO) [1]. The “validation” of an IVD device refers to the confirmation of the analytical and clinical performance characteristics (accuracy, precision, limits of detection and quantification, specificity, linearity, and robustness), according to the U.S. Food and Drug Administration (FDA)’s requirements of 510 k clearance, pre-market approval, or de novo submission, and to the EU IVD Regulation 2017/746 requirements [2]. “Evaluation” of an IVD device refers to a performance comparison (bias, agreement with reference methods), conducted by the manufacturer (as part of the validation process) and/or by end users. A target product profile (TPP) typically outlines the specifications that a new diagnostic kit is expected to fulfill (https://www.who.int/observatories/global-observatory-on-health-research-and-development/analyses-and-syntheses/target-product-profile/who-target-product-profiles (accessed on 10 June 2025)). Developers of diagnostic kits tend to use TPPs in their development and validation pipelines to ensure an end product meets expectations, for example, in terms of diagnostic sensitivity and specificity. The development, PQA, and validation of IVD devices (https://sites.bu.edu/qualityofmedicalproducts/files/2017/06/2017-WHO-PREQUALIFICATION-OF-IVDs.pdf (accessed on 10 June 2025)) requires biospecimens and associated data. These biospecimens should be fit for the intended purpose. Over the past 20 years, professional biobanks have been developed with the aim of providing fit-for-purpose biological resources to academic and industrial end users and product developers. Hence, the concept of a target specimen profile (TSP) was developed. A TSP report outlines the required characteristics of the specimen panels needed to demonstrate that a diagnostic kit meets the TPP.

## 2. Target Specimen Profile (TSP)

The purpose of a TSP is to provide a description of the biospecimen panel to support biobanks or biobank-hosting organizations in their specimen collection efforts.

We developed a standardized TSP template, including all of the qualitative and quantitative characteristics a specimen panel must possess to enable (i) the identification of new diagnostic biomarkers (development phase), (ii) the validation of the diagnostic method based on the identified biomarkers, including evaluation, but also (iii) the production of quality control materials to be used in the diagnostic kit, and (iv) the production of quality control materials to be used in external quality assurance (EQA) programs for the diagnostic method. This concept was applied to eight neglected tropical diseases (NTDs) (human African trypanosomiasis, cutaneous and visceral leishmaniasis, leprosy, lymphatic filariasis, onchocerciasis, schistosomiasis, soil-transmitted helminthiasis, and trachoma) and to two infectious diseases with pandemic potential: Nipah and Lassa fever.

The list of specifications corresponding to a TSP can be found in the corresponding TSP report, in the tables in the section “Panel needs”, and in the sections “Important biospecimen annotations” and “Need for samples corresponding to different strains”.

## 3. TSP Report Structure and Contents

Each TSP report includes an introduction with a general background about the specific infectious disease and justification of its diagnostic needs. Molecular biology assays, based on the detection or measurement of nucleic acids, and assays based on the detection or measurement of analytes other than nucleic acids, found in different types of biospecimens, are both within the scope of the report.

A TSP report for a given TSP includes the following specifications that the specimen panel must fulfill: information about the types of specimens, such as skin scrapings, slit skin, lesion swabs or skin lesion fine needs aspirate specimens, specifically applying to post-kala-azar dermal leishmaniasis (PKDL), in the example of the leishmaniasis TSP report, their necessary and useful annotations (metadata, clinical and biological annotations), such as Kato–Katz fecal examination-based egg counting results for *Schistosoma mansoni* and polycarbonate-filtered urine microscopy-based egg counting results for *Schistosoma haematobium*, as specified in the Schistosomiasis TSP report, their preanalytical specifications, their geographical origins, their reference analytical characterizations, their volumes and quantities, and the numbers of the various categories of patients and donors.

These specifications have been developed for each context in which new diagnostic kits may be used, such as disease elimination, disease surveillance, confirmation of suspected cases, and screening in areas of high or low endemicity. As an example, skin snips, urine, saliva, serum, plasma, and dry blood spot (DBS) specimens are needed in the context of mapping for prevalence of *Onchocerca volvulus*, while only serum, plasma, and DBS are needed in the context of the Massive Drug Administration (MDA) stopping decision-making, as specified in the Onchocerciasis TSP report.

The TSP reports also include a short review of existing commercialized diagnostic methods, as well as information about recognized reference methods, FDA-approved methods (https://www.fda.gov/medical-devices/device-advice-comprehensive-regulatory-assistance/medical-device-databases (accessed on 10 June 2025)), existing reference materials (https://www.nibsc.org/ (accessed on 10 June 2025)), and/or EQA programs (https://www.eptis.org/ (accessed on 10 June 2025)). Potential cross-reactivities are explained to justify the need for different types of negative controls. As an example, in the Human African Trypanosomiasis TSP report, it is specified that serum specimens containing antibodies to microfilariae and *Plasmodium* species are important to assess specificity.

Information about known genotypic and strain variability is included to justify the need to collect biospecimens from different geographical regions. As an example, in the Lassa virus TSP report, it is specified that collection of specimens from different endemic regions is important since virus strain variation at the amino acid level is as high as 12% and most serum samples have strain-specific antibodies.

Finally, informative annexes include information about potential sources of existing biospecimens, based on scientific publications or ongoing or completed clinical trials.

Detailed tables include guidance for the optimal number of patients/donors and optimal volumes for each type of biospecimen of interest.

Positivity status is unambiguously defined as an attribute of the patient/donor. As an example, in the Lymphatic Filariasis TSP report, it is explained that positivity for *Wucheria bancrofti, Brugia malayi,* or *Brugia timori* should be defined based on the presence of live worms.

Two methods can be used for the calculation of sample sizes. Sample sizes can be estimated according to Zhou et al.’s statistical approach [3] for diagnostic sensitivity and specificity levels, using https://finddx.shinyapps.io/SampleSize (accessed on 10 June 2025) at a significance level of α = 0.05 and for a full width of the 95% confidence interval of the evaluated sensitivity and specificity of 10%. Alternatively, sample sizes for the desired diagnostic sensitivity and specificity levels can be estimated according to Buderer’s statistical approach [4]. This uses PASS 2021 software at an actual significance level of between 0.05 and 0.15 and with 80% power for a diagnostic evaluation to detect a reduction in sensitivity or specificity of 10%.

The TSP report template can be used in any infectious disease area, but also beyond infectious diseases, with slight adaptations. For example, in oncology, a section would be needed, with specifications on the pathological Tumor–Node–Metastasis (pTNM) system and the specific molecular subtypes of cancer specimens. Ten TSP reports, illustrating the concept, can be found in the Supplementary Materials. The TSP report for leishmaniasis includes specifications for both cutaneous and visceral leishmaniasis. These illustrative TSP reports include specifications that are large enough to cover the needs of any diagnostic manufacturer, in a product- and company-agnostic way, based on the current state of the art. As technologies evolve, the specifications of the TSPs should also evolve. Finally, these TSP reports correspond to the ideal specimen panels, incurring significant costs for their collection. Hence, financial support would be needed, especially in Low- and Middle-Income Country (LMIC) biobanks to deploy such ideal TSPs.

## 4. Conclusions

We advocate for biobanks, the strategy of which is to support the diagnostics industry in developing and following TSPs. TSPs should be used to guide biobanks in the prospective collection of specimen panels to support the development and validation of new diagnostics and corresponding TPPs. The TSP concept should also help diagnostics developers to select the most appropriate specimen panels to support product development and validation. It has been shown that validation of new diagnostic biomarkers often fails [5]. Overall, the TSP concept is expected to decrease the number of failed diagnostic biomarkers, and to accelerate the development and implementation of diagnostics, including those for NTDs.

## Data Availability

All data are available in the Supplementary Materials.

## Supplementary Materials

The following supporting information can be downloaded at: https://www.mdpi.com/article/10.3390/diagnostics15121503/s1.
