# Supplementary material for: From Target Product Profiles (TPPs) to Target Specimen Profiles (TSPs): A New Concept in Infectious Disease Biobanking for Diagnostic Applications
Source: Diagnostics (Basel). 2025 Jun 13;15(12):1503. doi: 10.3390/diagnostics15121503 (PMC12191804; doi:10.3390/diagnostics15121503)
Supplement: Supplementary file 1 [file diagnostics-15-01503-s001.zip › diagnostics-3662952-supplementary.pdf]

FIND Diagnosis for all

SCHISTOSOMIASIS, TSP Report

Introduction

Schistosomiasis (bilharziasis) is caused by certain species of blood trematodes in the genus *Schistosoma*. The three main species infecting humans are S*chistosoma haematobium*, *S. japonicum*, and *S. mansoni*. Three other species, more localized geographically, are *S. mekongi*, *S. intercalatum,* and *S. guineensis*. There have also been a few reports of hybrid schistosomes of cattle origin (*S. haematobium*, *S. bovis*, *S. curassoni, S. mattheei*) infecting humans. Adult stages of *S. mansoni*, *S. japonicum*, *S. mekongi*, and *S. intercalatum* reside in the mesenteric venous plexus of infected hosts and eggs are shed in feces, while *S. haematobium* adult worms are found in the venous plexus of the lower urinary tract and eggs are shed in urine.

A *Schistosoma* virtual biobank is expected to support findability and availability of biospecimen panels for (i) EQA programs (or equivalent inter-laboratory exercises), reference and quality control material production (ii) method validation, including evaluation and/or PQA, and (iii) R&D other than validation of the analytical and clinical performance characteristics of a diagnostic test. Such R&D includes the identification of novel diagnostic biomarkers and/or their preliminary evaluation.

Scope

This document is intended as a description of the needs of biological materials in terms of *Schistosoma* panels.

The scope of this document includes the needs of biospecimens for development, then validation of molecular biology (nucleic acid based) assays and assays based on detection or measurement of analytes, other than nucleic acids, found in biological fluids (serum, plasma, urine, stool). Assays intended to be used both in a context of monitoring and evaluation and in a context of post-elimination surveillance are in the scope. *S. mansoni* and *S. haematobium* are the primary focus of this document.

The following are out of the scope of this document: morphological assays (microscopy based), assays on organ biopsies, assays specific for neuroschistosomiasis using CSF matrix, assays specific for genital schistosomiasis using genital sample matrices.

Sources

The content of this report is based on information found in the **References**. Periodic revision can be made as information on ongoing and scheduled diagnostic development projects, funded by different donors becomes available.

Interviews with Dr Lieven Stuyver (J&J) and Dr Henck Schallig (University Amsterdam) were also conducted.

Biological diagnosis of Schistosomiasis

Although microscopy-based assays are not in the scope of this document, traditional microscopy is still considered as the reference method and therefore biospecimen annotation with microscopy data is important.

Stool or urine samples can be examined microscopically for parasite eggs (stool for *S. mansoni* or *S. japonicum* eggs and urine for *S. haematobium* eggs). The eggs tend to be passed intermittently and in small amounts.

Examples of direct parasitological assays include the stool miracidium hatching test (based on the positive phototropic behavior of miracidia) and the urine syringe filtration, both combined with microscopy.

For increased sensitivity, optimal practice for *S. mansoni* is to collect three consecutive faecal samples on three consecutive days, with two slides for each sample. Optimal practice for *S. haematobium* is to collect three consecutive urine samples on three consecutive days.

Important biospecimen annotations

- Necessary annotation for *S. mansoni*: Kato-Katz faecal examination-based egg counting results.
- Necessary annotation for *S. haematobium*: polycarbonate filtered urine microscopy-based egg counting results
- Useful annotation for *S. mansoni*: POC-CCA results, if available
- Useful annotation for *S. haematobium*: urine dipstick for hematuria level

Nucleic acid based (molecular biology) assays

PCR based assays in serum/plasma or urine can target specific *Schistosoma* cfDNA or ccfRNA molecules. Critical preanalytical factors include the time of day, presence of stabilizer in the blood collection tube, the blood or urine pre-centrifugation time and temperature (time to centrifugation), the urine centrifugation conditions (uncentrifuged urine should be used), the serum/plasma or urine freezing temperature, the freeze-thaw cycles and the nucleic acid extraction kit or method used.

PCR based assays in faecal material specifically target *S. mansoni* DNA. Critical preanalytical factors include the stool stabilizing/fixative solution used for collection (non-formalin containing solution should be used), the homogenization modalities, and the nucleic acid extraction kit or method used.

As a sidenote, PCR in urine or feces, is generally specific and sensitive enough, to be used not only for diagnosis, but also for monitoring response to treatment.

Exosomal biomarkers in urine may be of interest for *S. haematobium* (Dr Henck Schallig, personal communication).

Non nucleic acid based (e.g. serological) assays

The *Schistosoma* acute infection phase extends for 12-16 weeks after infection. Serological assays for antibodies – in serum/plasma/dried blood spot samples - do not discriminate between active and past infection nor do they have therapeutic predictive value.

Assays for circulating antigens – in serum/plasma, whole blood, dried blood spot, or urine samples – include urine POC and lateral flow assays for circulating cathodic antigen CCA. Multiplex Lateral Flow assays have also been developed.

Critical preanalytical factors for analytes other than antibodies include the type of anticoagulant (for blood specimens), the time and temperature between collection and start of processing, the time and temperature between end of processing and freezing, and the long term freezing temperature.

Metabolite biomarkers in stool specimens may be of interest for *S. mansoni*, however a test based on stool metabolites would be impractical because of extreme sensitivity to preanalytical factors (Dr Lieven Stuyver, oral communication).

Potential serologic cross reactivities (important to inform the needs for validation of specificity, as relevant, depending on the analyte):

- When measuring antibodies, cross reactivity may exist against different schistosoma (co-infection *S. mansoni, S. haematobium*) and other helminth parasites (co-infection with *Ascaris lumbricoides, Trichuris trichiura*).
- When measuring CCA, specificity should be confirmed against *Strongyloides stercoralis, Fasciola* spp, *Taenia* spp. antigens. Furthermore, there may be serological cross reactivity between *S. mansoni* and *Plasmodium falciparum*.

According to Dr Leven Stuyver, cross reactions with *Onchocerca volvulus,* for some biomarkers of non-viable worms, probably exist (oral communication).

Table 1 gives the different contexts of use of diagnostic tests and corresponding sample types and method types.

Table 1

Context of use	Sample type and method
Monitoring and evaluation *S. mansoni*	Stool (NAAT),<br>Urine (immunoenzymatic method for CCA, CAA or other biomarker),<br>DBS (immunoenzymatic method for CAA or other biomarker)
Post-elimination surveillance *S. mansoni*	Serum/plasma (IgG ELISA)
Monitoring and evaluation *S. haematobium*	Urine (NAAT, immunoenzymatic method for CCA, CAA or other biomarker),<br>DBS (immunoenzymatic method for CAA or other biomarker)
Post-elimination surveillance *S. haematobium*	Serum/plasma (IgG ELISA)

Need for samples corresponding to different *S. mansoni* or *S. haematobium* strains:

Relevant geographical areas for *S. mansoni* biospecimen collection are East and West Africa, and South America, while for *S. haematobium* relevant areas are North and South Africa and Middle East. Some genetic differences among *Schistosoma* strains from different geographical areas have been identified using microsatellite markers. Although no impact on diagnostic performance has been reported till now, samples from multiple locations are desirable.

If *S. japonicum* positive samples are included, for purposes of validation of specificity, these would originate from the Philippines or China.

Reference methods:

- For *S. mansoni*: Kato-Katz faecal examination-based egg counting results.
- For *S. haematobium*: polycarbonate filtered urine microscopy-based egg counting results
- New reference method(s) with improved sensitivity and specificity need(s) to be established for live *S. mansoni* or *S. haematobium*

Reference materials

No WHO international standard exists for schistosomiasis.

FDA reference panels

No FDA reference panel for schistosomiasis could be found.

Validated (FDA approved) methods

- Anti-Schistosoma species kit (AMICO LAB Inc.) for *Schistosoma* (IgG, IgM)
- Amizyme Schistosoma spp. Species kit (AMICO LAB Inc.), for *S. mansoni* (IgG, IgM)

Other methods that have been made/are available

- Rapid tests are available from Maternova (Schistosomiasis rapid test) for *S. haematobium* and *S. mansoni* (CCA in urine) and from Rapid Medical Diagnostics for *S. haematobium* and *S. mansoni* (CCA in urine). However, WHO recommends POC-CCA to be used for *S. mansoni* only, not for *S. haematobium*.
- ELISA tests are available from MyBioSource, Demeditec, Abcam, ImmunoBiological Laboratories-America (IBL), Aviva Systems Biology, Creative Diagnostics.

No information on the composition of the panels that have been used for method validation by the companies could be found for any of these RDT or ELISA tests.

EQA programs

No commercially available EQA program for any parasitic disease and methods other than microscopy, except for toxoplasmosis, could be found on the CDC site.

On the EPTIS website, antibody-based serology schemes for *Schistosoma* are listed as being provided by the Reference Institute for Bioanalytics and by NEQAS.

The results from a recently organized academic EQA scheme (organized by the Parasitology Laboratory at Ghent University, Belgium, see Cools et al. with 12 stool items and 8 DNA items) have recently been published.

Panel needs

R&D / method validation needs

No data from any FDA submission that could be used to inform us on number of reference positive samples used for sensitivity assessment, number of reference negative samples for specificity assessment, number and type of samples for cross reactivity assessment, could be found.

Table 2a shows the needs in terms of panels for initial development /feasibility studies, for both nucleic acid-based and non-nucleic acid-based assays.

Table 2a

**Sample type**	**Quantity per donor (for one development panel)**	**Number of biospecimen donors (from multiple locations)**
Serum/ plasma	1ml	30-60 *S. mansoni* positive<br>30-60 *S. haematobium* positive<br>30-60 *Schistosoma* negative
Urine	2ml	30-60 *S. mansoni* positive<br>30-60 *S. haematobium* positive<br>30-60 *Schistosoma* negative
DBS	6 DBS (10μl)	30-60 *S. mansoni* positive<br>30-60 *S. haematobium* positive<br>30-60 *Schistosoma* negative
Stool	400mg	30-60 *S. mansoni* positive<br>30-60 *S. haematobium* positive<br>30-60 *Schistosoma* negative

Table 2b shows the needs in terms of complete validation panels for each indication and for both nucleic acid-based and non-nucleic acid-based assays. *S. japonicum* samples are included for purposes of validation of specificity.

Numbers of samples being based on the WHO TPP. Sample sizes have been estimated according to the NM Fenn Buderer statistical approach, for the desired diagnostic sensitivity and specificity levels, using PASS 2021 software at an actual significance level between 0.05 and 0.15 and with 80% power to detect a reduction in sensitivity or specificity of 10%.

Table 2b

Sample type	Quantity per donor (for 1 validation panel)	Number of biospecimen donors
Serum/ plasma	0,5ml	70(1)/20(2)/50(3) *S. mansoni* positive<br>70(1)/20(2)/50(3) *S. haematobium* positive<br>30(1)/30(2)/100(3) *S. japonicum* positive<br>200(1)/200(2)/700(3) *Schistosoma* negative
		Plus, 30 *Schistosoma* negative/and positive for any of the following: *Strongyloides spp, Fasciola spp, Taenia spp, Plasmodium falciparum*<br>*Applicable to antigen detection assays only*
Urine	1ml	70(1)/20(2)/50(3) *S. mansoni* positive<br>70(1)/20(2)/50(3) *S. haematobium* positive<br>30(1)/30(2)/100(3) *S. japonicum* positive<br>200(1)/200(2)/700(3) *Schistosoma* negative
Whole blood or DBS	0,2ml WB or 1 DBS	70(1)/20(2)/50(3) *S. mansoni* positive<br>70(1)/20(2)/50(3) *S. haematobium* positive<br>30(1)/30(2)/100(3) *S. japonicum* positive<br>200(1)/200(2)/700(3) *Schistosoma* negative
Stool	200mg	70(1)/20(2)/50(3) *S. mansoni* positive<br>70(1)/20(2)/50(3) *S. haematobium* positive<br>30(1)/30(2)/100(3) *S. japonicum* positive<br>200(1)/200(2)/700(3) *Schistosoma* negative

[1] applicable to a monitoring/evaluation context of use (assumed prevalence 30%, expected sensitivity 80%, expected specificity 97%, desired precision 10%). Collection should be from a region under endemic conditions.
[2] applicable to a surveillance context of use, as initial/screening test (assumed prevalence 5%, expected sensitivity 99%, expected specificity 60%, desired precision 10%). Collection should be from a region under elimination conditions.
[3] applicable to a surveillance context of use, as confirmatory test (assumed prevalence 5%, expected sensitivity 90%, expected specificity 99%, desired precision 10%). Collection should be from a region under elimination conditions.

Positivity status is an attribute of the donor. Positivity is defined by positive direct parasitological morphological assays with monomorphic results by a reference method, detecting active or live parasites, applied to at least one relevant biospecimen type from the donor.

Complete biospecimen sets, including all biospecimen types, from the same donor are preferred. It is critical that for a validation panel, all biospecimens come from the same collection, to avoid preanalytical bias. Preanalytical bias is unavoidable if using biospecimens from different collections with different or undocumented preanalytical specifications.

Possible retrospective sources of such specimens can be found in published literature and in completed or ongoing clinical trials (Annex 1). The most suitable sources would be **longitudinal** collections, including both pre-treatment and post-treatment samples.

Although the first priority is the needs for development, then for validation of new methods, in order for a new method to be deployed in the field, an external quality assurance (EQA) program is needed, as well as quality control (QC) materials to be included in the kits.

EQA needs

The needs for EQA panels for nucleic acid based and non-nucleic acid-based assays are listed below. Table 3 shows the minimum necessary quantities per panel for **one EQA scheme and round** for **40 participating laboratories**, with each laboratory receiving 0,1ml serum/plasma or urine, 200mg stabilized stool, and/or 1 DBS. The quantities correspond to the quantities to be distributed to the participating laboratories, supplemented by the quantities necessary to perform homogeneity and stability testing, and value assignment by the EQA provider.

The current best source of historical samples would be schistosomiasis screening centers in endemic countries.

Table 3

	Sample type	Quantity per item (for one EQA round)	Number of items
Non nucleic acid based	Serum (or plasma), antibody-based	6ml*	2 items *S. mansoni* positive<br>2 items *S. haematobium* positive<br>2 items *Schistosoma* negative
	Serum (or plasma), other biomarker-based	6ml*	2 items *S. mansoni* positive<br>2 items *S. haematobium* positive<br>2 items *Schistosoma* negative
Nucleic acid based	Urine	6ml*	2 items *S. haematobium* positive<br>2 items *Schistosoma* negative
	Stool	10g*	2 items *S. mansoni* positive<br>2 items *Schistosoma* negative
	DBS	60 spots	2 items *S. mansoni* positive<br>2 items *S. haematobium* positive<br>2 items *Schistosoma* negative

** can be pooled material*

Note. Urine and stool items for *Schistosoma* nucleic acid-based methods can be produced by spiking with DNA and/or RNA, extracted from *Schistosoma* worms, and corresponding to different copy number concentrations. Therefore, access to such clinical items can be considered as of secondary priority.

Commercialized kit, QC material needs

For one kit, depending on the kit specifications in terms of target and matrix, one of the following may apply

- At least 500ml of pooled *S. mansoni* serum/plasma, or
- At least 500ml of pooled *S. haematobium* serum/plasma, or
- At least 500ml of pooled *S. mansoni* urine, or
- At least 500ml of pooled *S. haematobium* urine

For antigen detection kits, an antigen solution can be used (no need for biological QC materials).

References consulted for the *Schistosoma* TSP

DG Colley et al. Human schistosomiasis. Lancet 2014;383:2253-2264

KGAD Weerakoon et al. Advances in the diagnosis of human schistosomiasis. Clin Microbiol Reviews 2015;28:939-967

R Hinz et al. Serological approaches for the diagnosis of schistosomiasis-a review. Mol Cell Probes 2017;31:2-21

J Utzinger et al. New diagnostic tools in schistosomiasis. Clin Microbiol Inf 2015;21:529-542

KG Weerakoon et al. DNA diagnostics for Schistosoma control, Tropical Med Inf Dis 2018;3:81

MG Cavalcanti et al. The advances in molecular and new point-of-care (POC) diagnosis of schistosomiasis pre- and post- praziquantel use: in the pursuit of more reliable approaches for low endemic and non-enedmic areas. Frontiers Immunol 2019;10:858

PLAM Corstjens et al. Tools for diagnosis, monitoring and screening of Schistosoma infections utilizing lateral-flow based assays and upconverting phosphor labels. Parasitology 2014;141:1841-1855

KJ Moendeg et al. Geographic strain differentiation of Schistosoma japonicum in the Philippines using microsatellite markers. PLOS Negl Trop Dis 2017;11:e0005749.

Global Schistosomiasis Alliance, Communication Piece (*confidential*), Commercially available diagnostic tests, 2020

https://www.cdc.gov/parasites/schistosomiasis/index.html

https://cdn.who.int/media/docs/default-source/ntds/schistosomiasis-(bilharzia)/call-for-consultation-tpps/who-tpp-scope-schistosomiasis-surveillance-narrative.pdf?sfvrsn=21d418c7_9

https://cdn.who.int/media/docs/default-source/ntds/schistosomiasis-(bilharzia)/call-for-consultation-tpps/who-tpp-scope-schistosomiasis-monitoring-evaluation-narrative.pdf?sfvrsn=93ac066a_11

https://path.azureedge.net/media/documents/2015.01.15_BMGF_SCH_postMDA_Ab.pdf

https://www.nibsc.org/products/brm_product_catalogue/who_standards.aspx

https://www.accessdata.fda.gov/scripts/cdrh/devicesatfda/index.cfm

https://www.biocompare.com/pfu/110627/soids/350163/ELISA_Kit/Schistosoma

https://www.eptis.org/

W Ammerlaan, F Betsou. Biospecimen science of human blood for cfDNA mutation analyses. Current Pathobiology Reports 2019, *7*(2): 9-15.

AF Harmon et al. Comparison of three different preservatives for morphological and real time PCR analysis of Haemonchus contortus eggs. Vet Parasitol 2007;145 :361-365.

FD Halstead et al. Universal extraction method for gastrointestinal pathogens. J Med Microbiol 2013;62:1535-1539.

P Cools et al. First international external quality assessment scheme of nucleic acid amplification tests for the detection of Schistosoma and soil-transmitted helminths, including Strongyloides: A pilot study. PLOS Negl Trop Dis 2020;14:e0008231.

NM Fenn Buderer. Statistical methodology: I. Incorporating the prevalence of disease into the sample size calculation for sensitivity and specificity. Acad Emerg Med 1996;3:895-900.

Acronyms

CAA	Circulating anodic antigen
CCA	Circulating cathodic antigen
ccfRNA	Circulating cell free RNA
cfDNA	Cell free DNA
CSF	Cerebrospinal fluid
DBS	Dry blood spot (venous blood or finger stick blood)
DTAG-NTD	Diagnostic Technical Advisory Group for Neglected Tropical Diseases
ELISA	Enzyme linked immunosorbent assay
EQA	External Quality Assurance
MTA	Material transfer agreement
NAAT	Nucleic acid amplification test
POC	Point of care
PQA	WHO prequalification assessment
RDT	Rapid diagnostic test

FIND
Diagnosis for all

Annex 1

Specifications of potential sources of biospecimens from published studies and from completed or ongoing clinical trials. Included are articles published after 2015 and clinical trials with at least 100 participants and completed after 2010.

Type of collection	Reference	Contact
Several hundred children and adults from Cote d'Ivoire<br><br>Urine and stool samples	doi: 10.1186/s12889-018-5044-2	Yves-Nathan T. Tian-Bi, Unité de Formation et de Recherche Biosciences, Université Félix Houphouët-Boigny, 22 BP 770, Abidjan, 22, Côte d'Ivoire
More than 1000 donors<br><br>Urine and stool samples	doi: 10.1111/tmi.12545	Richard Sanya, Medical Research Council/UVRI Uganda Research Unit on AIDS, Entebbe, Uganda
Urine samples from children But quite "old", from 2010-2012	https://clinicaltrials.gov/ct2/show/NCT00870649?term=bilhvax&rank=2	Dr. Gilles Riveau (Biomedical Research Center Espoir pour la Santé, St Louis, Senegal)
Urine and serum from 45 patients from the Philipinnes	doi: 10.1016/j.actatropica.2014.05.003	ychigusa@dokkyomed.ac.jp (Yuichi Chigusa) Laboratory of Tropical Medicine and Parasitology, Dokkyo Medical University, Mibu 321-0293, Tochigi, Japan
Urine and 3x stool samples from 250 donors from Brazil	doi: 10.1371/journal.pntd.0006232	stefan.geiger76@gmail.com Departamento de Parasitologia, Instituto de Ciências Biolo´gicas, Universidade Federal de Minas Gerais,<br>Belo Horizonte, Brasil
Urine and stool samples from 2000 children from Kenya	doi: 10.1186/s12889-018-5414-9	Collins Okoyo, comondi@kemri.org; collinsomondiokoyo@gmail.com Eastern and Southern Africa Centre of International Parasite

		Control, Kenya Medical Research Institute (KEMRI)
Urine and stool samples from 300 participants from Brasil	doi: 10.1590/0037-8682-0423-2016	Dr. José Roberto Lambertucci. jrlambertu@gmail.com Serviço de Doenças Infecciosas e Parasitárias, Departamento de Clínica Médica, Faculdade de Medicina, Universidade Federal de Minas Gerais, Belo, Horizonte, MG, Brasil
Stool samples from 100 participants from Brasil		Jose Mauro Peralta, peralta@micro.ufrj.br Departmento de Imunologia, Instituto de Microbiologia Paulo de Góes, Universidade Federal do Rio de Janeiro
Urine and stool samples samples from 300 children from Tanzania	doi: 10.1371/journal.pone.0202499	antje.fuss@medmissio.de Medical Mission Institute, Wuerzburg, German
Stool samples from 400 children from Brasil	doi: 10.1371/journal.pntd. 0006314	Pedro Fernandez-Soto pfsoto@usal.es Infectious and Tropical Diseases Research Group (e-INTRO), Biomedical Research Institute of Salamanca-Research Centre for Tropical Diseases at the University of Salamanca (IBSAL-CIETUS), Faculty of Pharmacy, University of Salamanca, Salamanca, Spain
Urine and stool samples from 100 children from Zambia	doi: 10.1371/journal.pone.0189400	nilanjan.lodh@marquette.ed Department of Clinical Laboratory Science, Marquette University, Milwaukee, Wisconsin, USA
Serum, urine and stool samples from 175 participants from Brasil	doi: 10.1016/j.actatropica.2018.03.002	Naftale Katz, nkatz@cpqrr.fiocruz.br Laboratory of Schistosomiasis, Instituto René Rachou, Fundação Oswaldo Cruz (Fiocruz), Avenida Augusto de Lima 1715, Belo Horizonte, Minas Gerais, 30190-002, Brazil.

FIND Diagnosis for all

Urine and stool samples from 150 participants from Brasil	doi: 10.1590/0037-8682-0070-2016	liliane_siqueira@cpqrr.fiocruz.br Laboratório de Esquistossomose, Centro de Pesquisas René Rachou, Fundação Oswaldo Cruz, Belo Horizonte, Minas Gerais, Brasil
Serum, urine and stool samples from 580 participants from Brasil	doi: 10.1371/journal.pntd.0006274	Carlos Graeff-Teixeira, graeff.teixeira@gmail.com Laboratorio de Biologia Parasitaria, School of Sciences, Pontifıcia Universidade Catolica do Rio Grande do Sul, Porto Alegre, Brazil
Urine and stool samples from 400 children from Yemen	doi: 10.3390/ijms160716085	Hesham M. Al-Mekhlafi , halmekhlafi@yahoo.com Department of Parasitology, Faculty of Medicine and Health Sciences, Sana'a University, 1247 Sana'a, Yemen ; Kek Heng Chua khchua@um.edu.my Department of Biomedical Science, Faculty of Medicine, University of Malaya, 50603 Kuala Lumpur, Malaysia
Urine samples from 1700 children from Pemba island	doi: 10.1371/journal.pntd.0003752	Stefanie Knopp, s.knopp@unibas.ch Wolfson Wellcome Biomedical Laboratories, Department of Life Sciences, Natural History Museum, London, United Kingdom, and Department of Epidemiology and Public Health, Swiss Tropical and Public Health Institute, Basel, Switzerland
Serum samples from 90 travelers/migrant s	doi: 10.1007/s10096-018-3303-x	Lisette van Lieshout lvanlieshout@lumc.nl Department of Parasitology, Leiden University Medical Center, L4-Q, PO Box 9600, 2300 RC Leiden, The Netherlands
Urine and stool samples from 80 and 160	doi: 10.1371/journal.pntd.0004778	Paulo Marcos Zech Coelho coelhopm@cpqrr.fiocruz.b

participants respectively from Brasil		Schistosomiasis Laboratory, Rene Rachou Research Center, Oswaldo Cruz Foundation (Fiocruz), Belo Horizonte, Minas Gerais, Brazil
Serum, urine and stool samples from 370 asylum seekers in Italy	PLoS Negl Trop Dis. (2017) 11:e0005593	dora.buonfrate@sacrocuore.it Centre for Tropical Diseases, Ospedale Sacro Cuore Don Calabria, Negrar, Verona, Italy
Serum, urine and stool samples from 100 migrants in Switzerland	doi: 10.1016/j.tmaid.2018.09.004	Niklaus D Labhardt Swiss Tropical and Public Health Institute, Basel, Switzerland; University of Basel, Basel, Switzerland; Division of Infectious Diseases and Hospital Epidemiology, University Hospital Basel, Basel, Switzerland
Urine and stool samples from 200 and 760 participants from Senegal and Kenya respectively	doi: 10.1371/journal.pntd.0003959	vanlieshout@lumc.nl , E.A.van_Lieshout@lumc.nl Department of Parasitology, Leiden University Medical Center, Leiden, The Netherlands
167 participants, Cote d'Ivoire	https://clinicaltrials.gov/ct2/show/NCT02868385?recrs=ae&cond=Schistosomiasis&draw=2&rank=2	Jean Coulibaly, Centre Suisse de Recherches Scientifiques en Côte d'Ivoire
700 children, Zimbabwe	https://clinicaltrials.gov/ct2/show/NCT02495909?recrs=ae&cond=Schistosomiasis&draw=2&rank=3	Francisca Mutapi, University of Edinburgh
350 participants, Zimbabwe	https://clinicaltrials.gov/ct2/show/NCT01424410?recrs=ae&cond=Schistosomiasis&draw=2&rank=4	Francisca Mutapi, University of Edinburgh
95 children, Senegal	https://clinicaltrials.gov/ct2/show/NCT03799510?recrs=ae&cond=Schistosomiasis&draw=2&rank=5	Modou DIOP, Biomedical Research Center ESPOIR POUR LA SANTE
106 donors, urine and serum samples	https://clinicaltrials.gov/ct2/show/NCT02194712?recrs=ae&cond=Schistosomiasis&draw=2&rank=9	M.P. Grobusch, VU University Medical Center P.J.J. van Genderen, Harbour Hospital Rotterdam

FIND Diagnosis for all

		M. Roestenberg, Leiden University Medical Center
335 children, serum and plasma, Egypt	https://clinicaltrials.gov/ct2/show/NCT02144389?recrs=ae&cond=Schistosomiasis&draw=2&rank=13	Rashika El Ridi, Cairo University
290 participants, serum and stool, Uganda*	https://clinicaltrials.gov/ct2/show/NCT03910972?recrs=ae&cond=Schistosomiasis&draw=2&rank=15	Hannah Kibuuka, Makerere University
200 participants, stool and urine, Egypt	https://clinicaltrials.gov/ct2/show/NCT01529710?recrs=ae&cond=Schistosomiasis&draw=2&rank=18	Ayat A Haggag, Ministry of Health
800 children, stool samples, Uganda	https://clinicaltrials.gov/ct2/show/NCT01901484?cond=Schistosomiasis&draw=2&rank=33	Allen Nalugwa, CHDC Makerere University
186 participants, urine and stool samples, Indonesia	https://clinicaltrials.gov/ct2/show/NCT03870204?cond=Schistosomiasis&draw=2&rank=36	Muhammad Karyana, Ina-Respond
311 children, stool and plasma samples, Côte d'Ivoire and Kenya	https://clinicaltrials.gov/ct2/show/NCT03845140?cond=Schistosomiasis&draw=2&rank=38	Eliezer N'Goran, eliezerngoran@yahoo.fr , University de Cocody; Pauline Mwinzi, pmwinzi@kemri.org , Kemri Kisumu
370 participants, serum, urine, placental blood, Philippines	https://clinicaltrials.gov/ct2/show/NCT00486863?cond=Schistosomiasis&draw=2&rank=39	RITM
120 participants, urine samples, Tanzania	https://clinicaltrials.gov/ct2/show/NCT03133832?cond=Schistosomiasis&draw=2&rank=41	Kun Yang, Jiangsu Institute of Parasitic Diseases
250 children, urine samples, Senegal	https://clinicaltrials.gov/ct2/show/NCT04635553?cond=Schistosomiasis&draw=2&rank=42	Bruno SENGHOR, bruno.senghor@ird.fr IRD
520 participants, urine samples, Sudan	https://clinicaltrials.gov/ct2/show/NCT01558336?cond=Schistosomiasis&draw=2&rank=43	Ishag Adam, Professor, University of Khartoum
100 participants, urine samples, Gabon	https://clinicaltrials.gov/ct2/show/NCT03779347?cond=Schistosomiasis&draw=2&rank=45	Ayola A ADEGNIKA, aadegnika@cermel.org, Josiane Y Honkpehedji, hyjosy@gmail.com Centre de Recherches Medicales de Lambaréné

726 children, stool and/or urine samples, Senegal	https://clinicaltrials.gov/ct2/show/NCT03893097?cond=Schistosomiasis&draw=2&rank=46	Moustapha Mbow, Institut de Recherche en Santé, de Surveillance Épidémiologique et de Formation (IRESSEF)
250 children, urine samples, Senegal	https://clinicaltrials.gov/ct2/show/NCT00870649?cond=Schistosomiasis&draw=2&rank=48	Gilles RIVEAU, INSERM
345 children, urine and stool samples, Ghana	https://clinicaltrials.gov/ct2/show/NCT01459146?cond=Schistosomiasis&draw=2&rank=52	Ernest C Opoku, Navrongo Health Research Centre, Ghana
414 participants, Ethiopia	https://clinicaltrials.gov/ct2/show/NCT01260012?cond=Schistosomiasis&draw=2&rank=55	Nega Berhe, nega_berhe@yahoo.com Aklilu Lemma Institute of Pathobiology, Addis Ababa University

ONCHOCERCIASIS, TSP Report

Introduction

Onchocerciasis is caused by nematodes (roundworms) *Onchocerca volvulus* that inhabit subcutaneous tissues.

A *Onchocerca* virtual biobank is expected to support findability and availability of biospecimen panels for (i) EQA programs (or equivalent inter-laboratory exercises), reference and quality control material production (ii) method validation, including evaluation and/or PQA, and (iii) R&D other than validation of the analytical and clinical performance characteristics of a diagnostic test. Such R&D includes the identification of novel diagnostic biomarkers and/or their preliminary evaluation.

Scope

This document is intended as a description of the needs of biological materials in terms of *Onchocerca* panels.

The scope of this document includes the needs of biospecimens for development, then validation of molecular biology (nucleic acid-based) assays on skin biopsies or fluid biospecimens, and assays based on detection or measurement of analytes, other than nucleic acids, found in biological fluids (serum, plasma, whole blood, urine). Assays intended to be used both in a context of mapping and in a context of MDA stopping are in the scope.

The following are out of the scope of this document: morphological assays (microscopy-based), intradermal assays. Ocular onchocerciasis is also out of the scope.

Sources

The content of this report is based on information found in the **References** and consultation with the **DTAG**-NTD on onchocerciasis. Periodic revision can be made as information on ongoing and scheduled diagnostic development projects, funded by different donors becomes available.

An interview with Dr Lieven Stuyver (J&J) was also conducted.

Biological diagnosis of Onchocerciasis

Although microscopy-based assays and endodermal assays are not in the scope of this document, traditional microscopy on the one hand, and endodermal Mazzoti reaction on the other hand, are still considered as reference methods and therefore biospecimen annotation with these data is important.

The most common method of diagnosis is the skin snip. A 1- to 2- mg shaving or biopsy of the skin is done to identify larvae, which emerge from the skin when it is put in physiologic solutions (e.g. normal saline). Typically, 6 snips are taken from different areas of the body. The diethylcarbamazine (DEC) patch test – develops a Mazzotti type reaction to microfilaria in the skin (not commercially available, and only tested in few published studies).

In patients with nodules in the skin, the nodule can be surgically removed and microscopically examined for adult worms.

Infections in the eye can be diagnosed with a slit-lamp examination of the anterior part of the eye where the larvae or the lesions they cause are visible.

Important biospecimen annotations

- Necessary annotation for *Onchocerca volvulus*: (i) skin snip microscopy or DEC patch test, (ii) Ov16 status
- Useful annotation for *Onchocerca volvulus*: nodule clinical palpation results

Nucleic acid based (molecular biology) assays

Polymerase chain reaction (PCR) of the skin snip allows laboratories to diagnose onchocerciasis.

Critical preanalytical factors for skin snips include the type of solution that is used to collect the skin snip and the DNA extraction kit or method used. Saline leads to microfilariae exiting the tissue. No formalin containing solution should be used. Critical preanalytical factors for fluid biospecimens include time and temperature between collection and start of processing, time and temperature between end of processing and storage, long term storage temperature, and DNA extraction kit or method used.

Non nucleic acid based (serological) assays

Serological assays for anti-*Onchocerca* antibodies use serum/plasma or dried blood spots.

Serological assays for antibodies – in serum/plasma/dried blood spot samples - do not discriminate between active and past infection nor do they have therapeutic predictive value.

Critical preanalytical factors include the long-term storage conditions.

Metabolite biomarkers in plasma or urine may be of interest (Dr Lieven Stuyver, oral communication).

Potential cross reactivities (important to inform the needs for validation of specificity, as relevant, depending on the analyte):

When performing PCR, specificity should be confirmed against *Mansonella streptocerca*.

When performing serology for anti-*Onchocerca* antibodies, specificity should be established against the following pathogens (most important ones, in bold):

> ***Loa loa, Mansonella ozzardi, Mansonella perstans, Mansonella streptocerca, Strongyloides stercoralis, Wuchereria bancrofti,*** *Brugia timori, Ascaris lumbricoides, Hymenolepsis nana, Schistosoma spp., Taenia solium, Toxocara spp., Trichuris spp., Entamoeba spp., Giardia intestinalis, Iodamoeba butschii.*

Table 1 gives the different contexts of use of diagnostic tests and corresponding sample types and method types.

Table 1

Context of use	Sample type and method
Mapping and identifying areas with >2% prevalence of *Onchocerca volvulus*	Skin snip, biological fluids (whole blood, urine, saliva) (NAAT)<br>Serum/plasma or DBS (IgG ELISA)
MDA stopping decision making and certifying areas with <1% prevalence of *Onchocerca volvulus*	Serum/plasma or DBS (IgG ELISA)

Need for samples corresponding to different *Onchocerca volvulus* strains:

The main relevant geographical area for *Onchocerca volvulus* biospecimen collection is Africa, while low and focal prevalence is observed in the Middle East and South America. Some morphological differences among *Onchocerca volvulus* strains from different geographical areas have been noticed, and samples originating from different countries are desirable.

Reference methods:

- Skin snip microscopy or PCR
- New reference method(s) need(s) to be established for live, female worm presence

Reference materials

No WHO international standard exists for onchocerciasis.

A recombinant IgG4 antibody has been proposed as a quality control material by PATH (Golden et al. 2016).

FDA reference panels

No FDA reference panel for onchocerciasis could be found.

Validated (FDA approved) methods

No FDA approved diagnostic method could be found.

Other methods that have been made/are available

- OV 16 RDT (SD Bioline)
- Ov 16 ELISA (SD Bioline, Abbott)
- Biplex RDT Onchocerciasis/Lymphatic Filariasis IgG4 (SD Bioline)
- OV16 IgG4 ELISA (Biorad AbD Serotec, ACROBiosystems)
- OV luciferase immunoprecipitation system (LIPS) assay (not commercialised)

The Biplex panel diagnostic performance has been established using 55 Ov negative/Wb negative, 20 *Strongyloides stercolaris* positive/Ov negative/Wb negative, 75 Wb positive and 75 Ov positive samples. This gives an idea of the type of panel that has been used to evaluate a commercial kit.

Some academic efforts have been made for identification of diagnostic metabolic biomarkers in urine or cfDNA or ccfRNA biomarkets in plasma, but have not been conclusive.

EQA programs

No commercially available EQA program for any parasitic disease and methods other than microscopy, except for toxoplasmosis, could be found on the CDC site.

No available EQA program could be found on the EPTIS website either.

Panel needs

R&D / method validation needs

Table 2a shows the needs in terms of panels for initial development /feasibility studies, for both nucleic acid-based and non-nucleic acid-based assays.

Table 2a

Sample type	Quantity per donor (for 1 development panel)	Number of biospecimen donors
Serum/ plasma	0,5-1ml[1]	30 *O. volvulus* positive cases<br>30 *O. volvulus* negative[2]<br>30 *O. volvulus* negative from LF positive cases[2,3]
Urine	2ml	30 *O. volvulus* positive cases<br>30 *O. volvulus* negative[2]<br>30 *O. volvulus* negative from LF positive cases[2,3]
Saliva	1ml	30 *O. volvulus* positive cases<br>30 *O. volvulus* negative[2]<br>30 *O. volvulus* negative from LF positive cases[2,3]
Whole blood or DBS	0,4ml WB or 2 DBS	30 *O. volvulus* positive cases<br>30 *O. volvulus* negative[2]<br>30 *O. volvulus* negative from LF positive cases[2,3]

[1]At least one sample, in 1ml amount

[2]For information, a diagnostic developer working on serological rapid tests, uses 80 negative samples from healthy donors and 100 negative samples from LF cases

[3]Samples from LF cases, collected in areas that are not endemic for onchocerciasis (eg., Asia, Pacific, some Latin America and Caribbean countries)

Table 2b shows the needs in terms of complete validation panels for each indication and for both nucleic acid-based and non-nucleic acid-based assays. Numbers of samples are based on the WHO TPP. Sample sizes have been estimated according to the NM Fenn Buderer statistical approach, for the desired diagnostic sensitivity and specificity levels, using PASS 2021 software at an actual significance level between 0.05 and 0.15 and with 80% power to detect a reduction in sensitivity or specificity of 10%.

For NAAT method validation using skin snips as the matrix, 2mg skin snips (equivalent to 3mm diameter) would be needed. However, this type of sample cannot be made readily available and no experimental animal model of *O. volvulus* infection exists, that could be used to collect skin snip samples as a surrogate. Therefore, for such methods, microfilariae suspensions or DNA samples extracted from them are the only possible sample type to be used. Such samples would be artificially prepared by a reference laboratory and would not be clinical biospecimens, hence they are not included in Table 2b.

Table 2b

**Sample type**	**Quantity per donor (for 1 PQA panel)**	**Number of biospecimen donors**
Serum/ plasma	0,5ml	90(1)/50(2) *O. volvulus* positive cases<br>900(1)/5000(2) *O. volvulus* negative/*W. bancrofti* positive cases<br>Mix of 900(1)/5000(2) cases, *O. volvulus* negative/and positive for any of the following: *Brugia malady, Brugia timori, Loa loa, Mansonella perstans, Mansonella ozzardi, Schistosoma mansoni,* ***Strongyloides stercoralis****, Taenia solium, Toxocara spp., Ascaris lumbricoides, Hymenolepsis nana, Entamoeba spp., Trichuris spp., Giardia intestinalis, Iodamoeba butschii*
Urine	1ml	90(1)/50(2) *O. volvulus* positive cases<br>900(1)/5000(2) *O. volvulus* negative/*W. bancrofti* positive cases<br>Mix of 900(1)/5000(2) cases, *O. volvulus* negative/and positive for any of the following: *Brugia malady, Brugia timori, Loa loa, Mansonella perstans, Mansonella ozzardi, Schistosoma mansoni,* ***Strongyloides stercoralis****, Taenia solium, Toxocara spp., Ascaris lumbricoides, Hymenolepsis nana, Entamoeba spp., Trichuris spp., Giardia intestinalis, Iodamoeba butschii*
Saliva	0,5ml	90(1)/50(2) *O. volvulus* positive cases

		900[1]/5000[2] *O. volvulus* negative/*W. bancrofti* positive cases<br>Mix of 900[1]/5000[2] cases, *O. volvulus* negative/and positive for any of the following: *Brugia malady, Brugia timori, Loa loa, Mansonella perstans, Mansonella ozzardi, Schistosoma mansoni,* ***Strongyloides stercoralis****, Taenia solium, Toxocara spp., Ascaris lumbricoides, Hymenolepsis nana, Entamoeba spp., Trichuris spp., Giardia intestinalis, Iodamoeba butschii*
Whole blood or DBS	0,2ml WB or 1 DBS	90[1]/50[2] *O. volvulus* positive cases<br>900[1]/5000[2] *O. volvulus* negative/*W. bancrofti* positive cases<br>Mix of 900[1]/5000[2] cases, *O. volvulus* negative/and positive for any of the following: *Brugia malayi, Brugia timori, Loa loa, Mansonella perstans, Mansonella ozzardi, Schistosoma mansoni,* ***Strongyloides stercoralis****, Taenia solium, Toxocara spp., Ascaris lumbricoides, Hymenolepsis nana, Entamoeba spp., Trichuris spp., Giardia intestinalis, Iodamoeba butschii*

[1] applicable to a mapping context of use (assumed prevalence 5%, expected sensitivity 60%, expected specificity 99,8%, desired precision 10%)
[2] applicable to an MDA stopping context of use (assumed prevalence 0,5%, expected sensitivity 89%, expected specificity 99,8%, desired precision 10%)

Positivity status is an attribute of the donor.

Positivity for Ov has been traditionally defined by positive skin snip direct parasitological morphological or PCR assay. However, for future diagnostic tests, positivity for Ov should be defined by presence of live, adult *O. volvulus* female worms. This might be achieved by automated microscopy of nodules, female worm-specific mRNA assays or other methods.

Positivity for Wb is defined by a positive blood microscopy assay.

Complete biospecimen sets, including all biospecimen types, from the same donor are preferred. It is critical that for a validation panel, all biospecimens come from the same collection, to avoid preanalytical bias. Preanalytical bias is unavoidable if using biospecimens from different collections with different or undocumented preanalytical specifications.

Possible retrospective sources of specimens can be found in published literature and in completed or ongoing clinical trials (Annex 1). The most suitable sources, especially relative to Ov positivity status based on live female worms, would be **longitudinal** collections, including both pre-treatment and post-treatment (or during MDA) samples.

Although the first priority is the needs for development, then for validation of new methods, in order for a new method to be deployed in the field, an external quality assurance (EQA) program is needed, as well as quality control (QC) materials to be included in the kits.

EQA needs

The needs for EQA panels for non-nucleic acid-based assays are listed below. Table 3 shows the minimum necessary quantities per panel for **one EQA scheme and round** for **40 participating laboratories**, with each laboratory receiving 1 DBS. The quantities correspond to the quantities to be distributed to the participating laboratories, supplemented by the quantities necessary to perform homogeneity and stability testing, and value assignment by the EQA provider.

The current best source of historical samples would be onchocerciasis screening centers in endemic countries.

Table 3

	Sample type	Quantity per item (for 1 EQA round)	Number of items
Non nucleic acid based	DBS	60 spots	4 items *O. volvulus* positive<br>1 item *W. bancrofti* positive<br>1 item *O. volvulus* and *W. bancrofti* negative

Note. Skin snip items for *Onchocerca* nucleic acid-based methods cannot be procured. The only possible item for NAAT EQA would be already extracted DNA (or RNA) samples with different copy number concentrations.

Commercialized kit, QC material needs

For one serology kit using serum or plasma,

- At least 500ml of pooled *O. volvulus* serum/plasma

For antigen detection kits, an antigen solution can be used (no need for biological QC materials).

References consulted for the *Onchocerca* TSP

T Lakwo et al. Onchocerciasis elimination: Progress and Challenges. Res Reports Trop Med 2020;11:81-95.

A Alhassan et al. Expanding the MDx toolbox for filarial diagnosis and surveillance. Trends Parasitol 2015;31:391-400

KR Feeser et al. Characterizing reactivity to Onchocerca volvulus antigens in multiplex bead assays. Am J Trop Med Hyg 2017;97:666-672

TR Unnasch et al. Diagnostics for onchocerciasis in the era of elimination. Int Health 2018;10:i20-i26

Report of the second meeting of the WHO onchocerciasis technical advisory subgroup. WHO, 2018

SD BIOLINE Oncho/LF IgG4 Biplex Technical File, 2016

ALERE Filariases Test Strip Technical Note

C Botto et al. Morphological differences between Venezuelan and African microfilariae of *Onchocerca volvulus.* J Helminthol 1988;62:345-351.

PD Burbelo et al. A four antigen mixture for rapid detection of *Onchocerca volvulus* infection. PLOS Negl Trop Dis 2009;3:e438.

A Golden et al. A Recombinant Positive Control for Serology Diagnostic Tests Supporting Elimination of Onchocerca volvulus. PLOS Negl Trop Dis 2016;10: e0004292.

CL Macfarlane et al. The insufficiency of circulating miRNA and DNA as diagnostic tools or as biomarkers of treatment efficacy for Onchocerca volvulus. Scient Reports 2020;10:6672.

https://www.cdc.gov/dpdx/onchocerciasis/

https://www.cdc.gov/parasites/onchocerciasis/health_professionals/index.html

https://apps.who.int/iris/bitstream/handle/10665/204180/9789241510011_eng.pdf

https://www.scielo.br/j/mioc/a/d5JX3vGT7xdK8XNgSqHfynq/?lang=en

https://path.azureedge.net/media/documents/Q5007_Anti-Ov16_and_Anti-Wb123_IgG4_Training_Panel_instructions_v.8_2017.04.12.pdf

https://www.who.int/publications/i/item/9789240024496

https://www.nibsc.org/products/brm_product_catalogue/who_standards.aspx

https://www.accessdata.fda.gov/scripts/cdrh/devicesatfda/index.cfm

https://www.eptis.org/

NM Fenn Buderer. Statistical methodology : I. Incorporating the prevalence of disease into the sample size calculation for sensitivity and specificity. Acad Emerg Med 1996;3:895-900.

Acronyms

DBS	Dry blood spot
DEC	Diethylcarbamazine
DTAG-NTD	Diagnostic Technical Advisory Group for Neglected Tropical Diseases
ELISA	Enzyme linked immunosorbent assay
EQA	External Quality Assurance
LF	Lymphatic filariasis
MDA	Massive drug administration
MTA	Material transfer agreement
NAAT	Nucleic acid amplification test
Ov	*Onchocerca volvulus*
POC	Point of care
PQA	WHO prequalification assessment
RDT	Rapid diagnostic test
QC	Quality control
Wb	*Wuchereria bancrofti*

Annex 1

Specifications of potential sources of biospecimens from published studies and from completed or ongoing clinical trials. Included are articles published after 2019 and clinical trials with at least 100 participants and completed after 2010.

Type of collection	Reference	Contact
90 children from Kenya<br><br>Plasma samples	doi.org/10.1186/s13071-019-3824-x	Olan Lagatie, Janssen Public Health Belgium, lagatie@its.jnj.com
420 donors from DRC<br><br>Skin snip, whole blood and serum samples	doi:10.3390/pathogens9060435	An Hotterbeekx, University of Antwerp, an.hotterbeekx@uantwerpen.be
330 donors from different countries in Africa and SE Asia<br><br>Skin snips and serum samples	doi: 10.4269/ajtmh.17-0756	Ole Lagatie, Janssen Public Health Belgium, lagatie@its.jnj.com1
65 donors from Cameroun<br><br>Urine samples	doi: 10.1186/s13071-021-04893-1	Peter Dormann, University Hospital Bonn, kenneth.pfarr@ukbonn.de achim.hoerauf@ukbonn.de
150 children from Uganda<br><br>Serum and CSF samples	doi: 10.1002/epi4.12463	Richard Idro, Makerere University, ridro1@gmail.com
1000 donors from Cameroun<br><br>DBS, blood smears on slides, skin samples	doi: 10.1093/cid/ciab255	Maria Gloria Basanez, Imperial College London, UK m.basanez@imperial.ac.uk

760 donors from different countries in Africa<br><br>Serum samples	doi:10.3390/pathogens9100847	Robert Colebunders, University Hospital Antwerp, robert.colebunders@uantwerpen.be
285 donors from DRC<br><br>Skin snips and DBS samples	doi:10.3390/pathogens9060435	An Hotterbeek, University Hospital Antwerp, an.hotterbeekx@uantwerpen.be
134 donors from DRC<br><br>Skin snip and urine samples	doi:10.3390/pathogens9030191	An Hotterbeek, University Hospital Antwerp, an.hotterbeekx@uantwerpen.be
200 donors from Kenya<br><br>Plasma samples	doi: 10.1186/s13071-019-3824-x	Ole Lagatie, Janssen Public Health Belgium, lagatie@its.jnj.com
90 donors from Ghana<br><br>Serum samples	doi: 10.1007/s00436-019-06345-3	Ole Lagatie, Janssen Public Health Belgium, lagatie@its.jnj.com
170 donors from Cameroon<br><br>Skin snip and serum samples	doi: 10.1093/cid/ciz172	Joseph Kamgno, Centre for Research on Filariasis and Other Tropical Diseases (CRFilMT), Cameroon, kamgno@crfilmt.org
1000 children from Mali<br><br>DBS samples	doi: 10.1371/journal.pntd.0007064	Housseini Dolo, Point G, Mali, hdolo@icermali.org
440 participants, skin snips and blood samples, DRC	https://clinicaltrials.gov/ct2/show/record/NCT04913610?recrs=ae&cond=Onchocerciasis&draw=2&rank=5	sspecht@dndi.org
100 participants, skin snip and	https://clinicaltrials.gov/ct2/show/record/NCT03052998?recrs=ae&cond=Onchocerciasis&draw=2&rank=11	Michel Mandro, University Antwerp

blood samples, DRC		
14000 participants, skin snip, serum and stool samples, Ivory Coast	https://clinicaltrials.gov/ct2/show/record/NCT02032043?recrs=ae&cond=Onchocerciasis&draw=2&rank=12	Gary Weil and Peter Fischer, Washington University School of Medicine
200 participants, skin snip and serum samples, DRC	https://clinicaltrials.gov/ct2/show/record/NCT03852303?recrs=ae&cond=Onchocerciasis&draw=2&rank=14	Robert Colebunders, University Antwerp

Human African Trypanosomiasis, TSP Report

Introduction

Human African Trypanosomiasis (HAT) or sleeping sickness is a vector borne disease, caused by the two extracellular trypanosome subspecies *Trypanosoma brucei gambiense (Tbg)* and *Trypanosoma brucei rhodesiense (Tbr)*. This kinetoplastid disease is transmitted by tsetse flies (*Glossina* sp) and is lethal if left untreated. *Trypanosoma brucei* affects the blood, lymph and peripheral organs (stage 1), and at a later stage, the central nervous system (CNS) (stage 2). *Tbg* disease has a chronic (months/years) evolution, while *Tbr* is acute (weeks/months) and causes multi-organ failure.

After long periods with hundreds of thousands of cases per year, the annual incidence is now below 1,000 new cases. The WHO had set a 2020 goal for elimination of *Tbg* as public health problem. This objective has been achieved in a few countries and a new 2030 goal for interruption of transmission, has been set. The objective is now to sustain the elimination efforts in order to achieve the 2030 target of zero cases, and interruption of transmission. The frequencies recommended by WHO for the implementation of active screening are the following:

**Prevalence**	**Screening frequency**
≥1 case/1000 population/year	Once/year
1 case/10 000 population/year - 1 case/1000 population/year	Once every 2 years
<1 case/10 000 population/year	No active screening

Currently available treatment options are not always safe and not easy to deploy logistically. First line drugs at the hemolymphatic stage of disease include pentamidine (for *Tbg*), and suramin (for *Tbr*), and at the CNS stage, melarsoprol (for *Tbr*) and nifurtimox in combination with eflornithine (NECT) (for *Tbg*). Newer drugs are fexinidazole (in use) and acoziborole (in clinical trials).

Most importantly, non-invasive diagnosis of stage 2 sleeping sickness, which is necessary in order to determine the treatment to be used, remains a challenge.

A *Trypanosoma brucei* virtual biobank is expected to support findability and availability of biospecimen panels for (i) EQA programs (or equivalent inter-laboratory exercises), reference and quality control material production (ii) method validation, including evaluation and/or PQA and (iii) R&D other than validation of the analytical and clinical performance characteristics of a diagnostic test. Such R&D includes the identification of novel diagnostic biomarkers and/or their preliminary evaluation.

Scope

This document is intended as a description of the needs of biological materials in terms of HAT panels.

The scope of this document includes the needs of biospecimens for development, then validation of molecular biology (nucleic acid-based) assays, and assays based on detection or measurement of analytes, other than nucleic acids, found in biological fluids (serum, plasma, whole blood or buffy coat, lymph node aspirate, urine, saliva, CSF). Assays intended to be used both in a context of screening and staging of Trypanosoma brucei infections are in the scope. Assays based on host-derived biomarkers and *Tbg*- or *Tbr* –derived biomarkers are also in the scope.

The following are out of the scope of this document: polysomnography, actigraphy and microscopy-based examination assays.

Sources

The content of this report is based on information found in the **References** and consultation with the **DTAG**-NTD on HAT. Periodic revision can be made as information on ongoing and scheduled diagnostic test development projects, funded by various donors becomes available.

An interview with Dr José Ramon Franco (WHO) was also conducted (12th April 2022).

Clinical and biological diagnosis of HAT

Biological diagnosis is critical since clinical symptoms are not specific. Overall, the gold standard for the biological diagnosis of HAT is based on demonstration of parasites by direct parasitological analysis. The context for HAT diagnosis includes (i) screening and confirmation, based on venous or capillary blood and/or lymph node puncture, followed by (ii) staging, for which CSF is needed. Efficient biological diagnosis of HAT at stage 1 would reduce the cases getting to stage 2 of the disease.

In a screening context, serological diagnosis is available for *Tbg*, but not for *Tbr* for which diagnosis relies only on microscopy. Direct parasitological diagnosis of HAT requires microscopic examination, which shows around 80% sensitivity at best. Preanalytical complications of microscopy are linked to the trypanosome fragility and to the need for blood or CSF concentration. Concentration methods include capillary tube centrifugation (CTC), or mini-anion exchange centrifugation technique (mAECT). Two sequential tests, with the second being a microscopical one, offer optimal sensitivity (to avoid HAT related deaths in case undiagnosed cases are left untreated) and specificity (to avoid drug toxicity in false positive cases). Hence, the gold standard for the diagnosis of HAT is positive serology followed by positive direct parasite detection in blood, lymph node aspirate or chancre fluid. Specificity is critical for screening in very low prevalence areas.

In a staging context, a lumbar puncture is required for CSF collection and parasite (motile trypomastigote) detection. A field-compatible polysomnography for detection of PSG, as well as actigraphy with wrist wearables have been suggested as possible non-invasive examinations prior to CSF examination. Elevated white blood cells in the CSF, >20 cells per μl (applicable in Angola; information J. Ndungu) and >5 cells per μl, is a host biomarker indicating stage 2 *Tbg* and *Tbr* HAT respectively. A gold standard for staging diagnosis does not exist.

DNA and/or RNA, extracted from different types of biospecimens mentioned above can be useful for development of direct diagnostic assays.

Important biospecimen annotations

- Necessary annotations for *Trypanosoma brucei*: geographic origin, presence of chancre, presence of enlarged lymph nodes, edema, splenomegaly, hypergammaglobulinemia, headaches, hyperesthesia or other neurological symptoms, microscopy results, CATT or RDT results.
- Useful annotations for *Trypanosoma brucei*: preanalytical data (time to centrifugation/freezing/stabilization, storage temperature), CBC results. Treatment specifications and treatment outcome data are also useful, especially in the validation phase.

Nucleic acid based (molecular biology) assays

PCR, NASBA or LAMP assays can be used for direct detection of the parasite DNA or RNA in blood, urine or saliva, lymph node aspirate, and in CSF at the CNS stage of the disease. However, CSF is not a priority sample type for diagnostic assays readily accessible in the field. Specific targets include the pan-*T. brucei* RIME sequence (LAMP) , or the *Tbr*-specific serum resistance associated (SRA) gene, the *Tbg*-specific glycoprotein coding gene, or the expression site associated genes 6 and 7 (ESAG 6/7), 18S ribosomal DNA, multi-copy satellite DNA or internal transcribed spacer (ITS) targets. More than one sampling may be required since *Tbg* parasitemia shows high day-to-day variability. *Tbr* parasitemia is more consistent and more elevated, which makes NAAT more relevant.

Circulating parasitic small non-coding RNAs might represent early diagnostic biomarkers, preceding the onset of parasitaemia.

Critical preanalytical factors, for all types of fluid samples, include time and temperature between collection and start of processing, time and temperature between end of processing and storage, long term storage temperature, and DNA or RNA extraction kit or method used. Other critical preanalytical factors are the type of anticoagulant for whole blood and blood derivatives (buffy coat, serum, plasma), the use of 3% PSGlu in the blood collection tube (to extend trypanosome viability), the centrifugation conditions and use of stabilizer for urine, the needle gauge, hemoglobin contamination, centrifugation conditions, type of storage container for CSF.

Non nucleic acid based (serological or cellular) assays

Serological diagnosis is only possible for *Tbg*. Card agglutination tests for trypanosomiasis (CATT) are used in the field for screening purposes, and are based on detection of antibodies against the variant surface glycoprotein (VSG) variable antigen types (VAT) LiTat1.3 or LiTat1.5. RDTs also exist for point of care serological testing. Immunofluorescence and ELISA tests exist, but they are generally not used in the field.

Serological assays do not discriminate active from past infection since antibodies may persist for years after treatment.

Trypanolysis (TL) tests, which are antibody-mediated complement lysis tests, can be used as confirmatory tests in serology-positive, but microscopy- or NAAT-negative cases, and are recognized as reference tests by the WHO. An inhibition ELISA assay offers similar diagnostic performance, with higher throughput and less biohazard than TL.

Host-based biomarkers include IFNγ produced by CD8+ lymphocytes, which is due to the trypanosome lymphocyte triggering factor. Host-based biomarkers for staging include WBCs, but also total protein concentration (>370mg/L), IgM or inflammatory cytokines and chemokines in CSF. A staging RDT based on neopterin or CXCL13 has been proposed. Raman spectroscopic analysis of skin has recently been proposed as an innovative diagnostic approach.

For antibody-based assays, critical preanalytical factors include the long-term storage conditions. For protein and/or metabolite-based assays, critical preanalytical factors include the type of anticoagulant, the time and temperature between collection and start of processing or analysis, the centrifugation conditions, the time and temperature between end of processing and cryopreservation, the long-term storage conditions.

Potential cross reactivities (important to inform the needs for validation of specificity, as relevant, depending on the analyte):

Antibodies against microfilariae and *Plasmodium* species.

Table 1 gives the different contexts of use of diagnostic tests and the corresponding most important sample types and method types.

Table 1

Context of use	Sample type and method
Screening of HAT	Serum/plasma, urine, saliva or DBS (IgG/IgM ELISA or other biomarkers)<br>Serum/plasma, urine, saliva or DBS (NAAT)
Staging of HAT	CSF (NAAT or other biomarkers)

Need for samples corresponding to different *Trypanosoma brucei* strains:

The main relevant geographical area for *Tbg* biospecimen collection is western and central Africa, while the most relevant geographical area for *Tbr* biospecimen collection is eastern and southern Africa.

Genetic markers differentiate the two subspecies, with the serum resistance associated (*SRA*) gene being specific to *Tbr*, but also antigenic variation occurs inside each subspecies. Certain strains do not express the LiTat1.3 antigen on which CATT is based. Furthermore, there are two distinct sub-groups of *Tbg* that differ genetically and by human serum resistance phenotypes. For all these reasons, samples from different countries are needed, not only for the validation phase (Table 2b), but also for the development phase (Table 2a).

Reference methods:

- Microscopy on different types of biological fluids

Reference materials

No WHO international standard exists for HAT.

FDA reference panels

No FDA reference panel for HAT could be found.

Validated (FDA approved) methods

No FDA approved diagnostic method could be found.

Other methods that have been described/made available

- HAT Sero-K-SeT (Coris Bioconcept, Belgium)
- rHAT Sero-Strip (Coris Bioconcept, Belgium)
- SD Bioline HAT 1 (Standard Diagnostics, South Korea), both based on native surface glycoproteins LiTat1.3 and LiTat1.5 (as per Buscher 2013)
- SD Bioline HAT 2, based on recombinant antigens.
- An assay based on native and recombinant surface glycoprotein MiTat1.4 (as per Sullivan 2013)
- An RUO inhibition ELISA for *Tbg* (Advanced Practical Diagnostics, Belgium)

- Loopamp *Trypanosoma brucei* kit (Eiken Chemical Co LTD, Japan; available on specific order)
- African Trypanosomiasis Real-Time PCR kit (nzytech, Portugal)
- M18S qPCR and TgsGP qPCR (as per Compaoré 2020)
- An innovative CRISPR-based method has been very recently published, with claimed potential to be further developed into a POC (Sima et al 2022)

No information on the composition of the panels that have been used for method validation by the companies could be found for any of these RDT or PCR tests.

EQA programs

The only available EQA program that could be found on the EPTIS website is a scheme based on digitalized slides (Deutsches Referenzburo fur ringversuche und referenzmaterialien GbH, Germany).

Panel needs

R&D / method validation needs

Table 2a shows the needs in terms of panels for initial development /feasibility studies, for both nucleic acid-based and non-nucleic acid-based assays.

Table 2a

Sample type	Quantity per donor (for 1 development panel)	Number of biospecimen donors
Serum/ plasma	1ml	20 *Tbg* serologically and parasitologically positive cases<br>20 *Tbr* parasitologically positive cases<br>20 *Tbg* serologically and parasitologically negative cases<br>20 *Tbr* parasitologically negative cases
Urine	2ml	20 *Tbg* serologically and parasitologically positive cases<br>20 *Tbr* parasitologically positive cases<br>20 *Tbg* serologically and parasitologically negative cases<br>20 *Tbr* parasitologically negative cases
Saliva	1ml	20 *Tbg* serologically and parasitologically positive cases<br>20 *Tbr* parasitologically positive cases<br>20 *Tbg* serologically and parasitologically negative cases<br>20 *Tbr* parasitologically negative cases

Whole blood or DBS	0,4ml WB or 2 DBS	20 *Tbg* serologically and parasitologically positive cases<br>20 *Tbr* parasitologically positive cases<br>20 *Tbg* serologically and parasitologically negative cases<br>20 *Tbr* parasitologically negative cases
CSF*	0,5ml	20 *Tbg* serologically and parasitologically positive cases<br>20 *Tbr* parasitologically positive cases<br>20 *Tbg* serologically and parasitologically negative cases<br>20 *Tbr* parasitologically negative cases

*not the preferred sample type

Tbg negative cases should ideally be from areas that are not endemic for any form of trypanosomiasis, including livestock forms.

Table 2b shows the needs in terms of complete validation panels for each indication and for both nucleic acid-based and non-nucleic acid-based assays. Numbers of donors are defined based on the WHO TPP.

Table 2b

Sample type	Quantity per donor (for 1 panel)	Number of biospecimen donors
Serum/ plasma	0,5ml	30(1) *Tbg* serologically and parasitologically positive cases<br>30(2) *Tbr* parasitologically positive cases<br>900(1)/900(2) *Tbg* serologically and parasitologically negative, and *Tbr* parasitologically negative cases<br>900(1)/900(2) *Tbg* serologically positive and parasitologically negative, and *Tbr* parasitologically negative cases<br>Mix of 900(1)/900(2) cases, *T. brucei* serologically and parasitologically negative, of which half being positive for *Plasmodium sp*. or microfilariae
Urine	1ml	30(1) *Tbg* serologically and parasitologically positive cases<br>30(2) *Tbr* parasitologically positive cases<br>900(1)/900(2) *Tbg* serologically and parasitologically negative, and *Tbr* parasitologically negative cases<br>900(1)/900(2) *Tbg* serologically positive and parasitologically negative, and *Tbr* parasitologically negative cases<br>Mix of 900(1)/900(2) cases, *T. brucei* serologically and parasitologically negative, of which half being positive for *Plasmodium sp*. or microfilariae
Saliva	0,5ml	30(1) *Tbg* serologically and parasitologically positive cases<br>30(2) *Tbr* parasitologically positive cases<br>900(1)/900(2) *Tbg* serologically and parasitologically negative, and *Tbr* parasitologically negative cases<br>900(1)/900(2) *Tbg* serologically positive and parasitologically negative, and *Tbr* parasitologically negative cases<br>Mix of 900(1)/900(2) cases, *T. brucei* serologically and parasitologically negative, of which half being positive for *Plasmodium sp*. or microfilariae

Whole blood or DBS	0,2ml WB or 1 DBS	30$^{(1)}$ *Tbg* serologically and parasitologically positive cases<br>30$^{(2)}$ *Tbr* parasitologically positive cases<br>900$^{(1)}$/900$^{(2)}$ *Tbg* serologically and parasitologically negative, and *Tbr* parasitologically negative cases<br>900$^{(1)}$/900$^{(2)}$ *Tbg* serologically positive and parasitologically negative, and *Tbr* parasitologically negative cases<br>Mix of 900$^{(1)}$/900$^{(2)}$ cases, *T. brucei* serologically and parasitologically negative, of which half being positive for *Plasmodium sp*. or microfilariae
CSF*	0,1ml	30$^{(1)}$ *Tbg* serologically and parasitologically positive cases<br>30$^{(2)}$ *Tbr* parasitologically positive cases<br>900$^{(1)}$/900$^{(2)}$ *Tbg* serologically and parasitologically negative, and *Tbr* parasitologically negative cases<br>900$^{(1)}$/900$^{(2)}$ *Tbg* serologically positive and parasitologically negative, and *Tbr* parasitologically negative cases<br>Mix of 900$^{(1)}$/900$^{(2)}$ cases, *T. brucei* serologically and parasitologically negative, of which half being positive for *Plasmodium sp*. or microfilariae

[1] applicable to a context of confirmation of suspected and parasitologically unconfirmed *Tbg* (assumed prevalence inside a highly endemic area of 1% positive screening results, expected sensitivity 99%, expected specificity 99%, desired precision 10%)
[2] applicable to a context of screening/detection of *Tbr* (assumed prevalence inside a highly endemic area 1%, expected sensitivity 99%, expected specificity 99%, desired precision 10%)
*not the preferred sample type

Positivity status, as an attribute of the donor/patient, encompasses both serological and parasitological positivity. Positivity for sleeping sickness stage 1 is defined by positive serology and positive microscopy in blood, urine, saliva or other biological fluids. Positivity for sleeping sickness stage 2 is defined by positive serology, and positive microscopy in CSF or more than 5 white cells/ul in CSF.

Sample sizes have been estimated according to the NM Fenn Buderer statistical approach, for the desired diagnostic sensitivity and specificity levels, using PASS 2021 software at an actual significance level between 0.05 and 0.15 and with 80% power to detect a reduction in sensitivity or specificity of 10%.

Complete biospecimen sets, including all biospecimen types, from the same donor are preferred. It is critical that for a validation panel, all biospecimens come from the same

collection, to avoid preanalytical bias. Preanalytical bias is unavoidable if using biospecimens from different collections with different or undocumented preanalytical specifications.

Possible retrospective sources of specimens can be found in published literature and in completed or ongoing clinical trials (Annex 1). The most suitable sources for identification and validation of stage-specific or treatment efficacy biomarkers would be **longitudinal** collections from geographically different endemic areas, with pre-and post-treatment sampling of patients. With the very low global incidence that is currently observed, an approach based on Identification of Villages at Risk (IVR) might be necessary.

WHO has established and is the custodian of a HAT biobank, including blood, serum, plasma, saliva, urine and CSF specimens from “cases” (confirmed presence of trypanosomes), “controls” (negative CATT and parasitology) and “suspects” (positive CAT; negative parasitology), with follow-up sampling. Specimens were collected in Guinea, DRC, Chad, Uganda, Tanzania and Malawi. The web-link to this biobank is http://www.who.int/trypanosomiasis_african/research/en. Another HAT biobank has been established at the ITM in Antwerp, while the Trypa-NO! Partnership, established in 2016, supports National Sleeping Sickness Control programs (NSSCP), based on screening and diagnosis operations amongst others.

Although the first priority is the needs for development, then for validation of new methods, in order for a new method to be deployed in the field, an external quality assurance (EQA) program is needed, as well as quality control (QC) materials to be included in the kits.

EQA needs

The needs for EQA panels for non-nucleic acid-based assays are listed below. Table 3 shows the minimum necessary quantities per panel for **one EQA scheme and round** for **40 participating laboratories**, with each laboratory receiving 1 DBS. The quantities correspond to the quantities to be distributed to the participating laboratories, supplemented by the quantities necessary to perform homogeneity and stability testing, and value assignment by the EQA provider.

The best retrospective source of samples would be HAT screening centers in endemic countries.

Table 3

	Sample type	Quantity per item (for 1 EQA round)	Number of items
Non nucleic acid based or nucleic acid based	DBS	60 spots	3 items *Tbg* and/or *Tbr* positive (for the target assay)<br>1 items *Tbg* and *Tbr* negative (for the target assay)

Commercialized kit, QC material needs

For one kit using serum or plasma,

- At least 500ml of pooled *Tbg* or *Tbr* serum/plasma

References consulted for the *HAT* TSP

JR Franco et al. The Human African Trypanosomiasis specimen biobank: a necessary tool to support research of new diagnostics. PLoS Negl Trop Dis 2012;6:e1571.

PP Simarro et al. Diversity of human African trypanosomiasis epidemiological settings requires fine tuning control strategies to facilitate disease elimination. Res Rep Trop Med 2013;4:1-6.

https://www.who.int/news-room/fact-sheets/detail/trypanosomiasis-human-african-(sleeping-sickness)

https://apps.who.int/iris/rest/bitstreams/1414635/retrieve

https://apps.who.int/iris/rest/bitstreams/1363807/retrieve

https://apps.who.int/iris/bitstream/10665/70809/1/WHO_HTM_NTD_2012.1_eng.pdf

PI Akazue et al. Sustainable elimination (zero cases) of sleeping sickness. How far are we from achieving this goal? Pathogens 2019;8:135.

P Capewell et al. Differences between *Trypanosoma brucei gambiense* Groups 1 and 2 in their resistance to killing by trypanolytic factor 1. PLoS Negl Trop Dis 2011;5:e1287.

N Van Meirvenne et al. Evaluation of variant specific trypanolysis tests for serodiagnosis of human infections with *Trypanosoma brucei gambiense*. Acta Trop 1995;60:189-199.

E Dama et al. Immune trypanolysis test as a potential bioassay to monitor the elimination of gembiense human African trypanosomiasis. Parasite 2019;26:68.

MP Barrett & SL Croft. Management of trypanosomiasis and leishmaniasis. Brit Med Bull 2012;104:175-196.

ZK Njiru et al. Loop mediated isothermal amplification (LAMP) method for rapid detection of *Trypanosoma brucei rhodesiensis*. PLoS Negl Med Dis 2008;2:e147.

CM Mugasa et al. Diagnostic accuracy of molecular amplification tests for human African trypanosomiasis – systematic review. PLoS Negl Trop Dis 2012;6:e1438.

P Mitashi et al. Diagnostic accuracy of loopamp *Trypanosoma brucei* detection kit for diagnosis of human african trypanosomiasis in clinical samples. PLoS Negl Trop Dis 2013;7:2504.

CM Mugasa et al. Comparison of nucleic acid sequence-based amplification and loop-mediated isothermal amplification for diagnosis of human African trypanosomiasis. Diagn Microbiol Infect Dis 2014;78:144-148.

B Bouteille & A Buguet. The detection and treatment of human African trypanosomiasis. Res Reports Trop Med 2012;3:35-45.

P Buscher et al. Rapid diagnostic test for sleeping sickness. New Engl J Med 2013;368:1069-1070.

AK Njamnshi et al. The actigraphy sleep score: a new biomarker for diagnpsis, disease staging, and monitoring in human African trypanosomiasis. Am J Trop Med Hyg 2020;103:2244-2252.

L Sullivan et al. Proteomic selection of immunodiagnostic antigens for human African trypanosomiasis and generation of a prototype lateral flow immunodiagnostic device. PLoS Negl Trop Dis 2013;7:e2087.

JM Sternberg et al. Evaluation of the diagnostic accuracy of prototype rapid tests for human African trypanosomiasis. PLoS Negl Trop Dis 2014;8:e3373.

D Mumba Ngoyi et al. Performance of parasitological and molecular techniques for the diagnosis and surveillance of *gambiense* sleeping sickness. PLoS Negl Trop Dis 2014;8:e2954.

M Geerts et al. *Trypanosoma brucei gambiense* – iELISA : a promising new test for the post-elimination monitoring of human African trypanosomiasis. Clin Inf Dis 2021;73:e2477.

J Bonnet et al. Overview of the diagnostic methods used in the field for Human African Trypanosomiasis; what could change in the next years? Biomed Res Int 2015;583262.

FIND, Developing new diagnostic tests for Human African Trypanosomiasis. Current status and future plans. Foundation for Innovative New Diagnostics; Geneva, 2013, 1-20.

JR Franco et al. The Human African Trypanosomiasis specimen bank: a necessary tool to support research of new diagnostics. PLoS Negl Trop Dis 2012;6:e1571.

P Steinmann et al. Contemporary and emerging strategies for eliminating human African trypanosomiasis due to *Trypanosoma brucei gambiense*: review. Trop Med Int Health 2015;20:707-718.

A Mpanya et al. Direct comparison of the card agglutination test for trypanosome (CATT) and a rapid diagnostic test in a highly endemic district. Trop Med Int Heal 2015;20:325-326.

SM Chiwese et al. Parasite specific 7SL-derived small RNA is an effective target for diagnosis of active trypanosomiasis infection. PLoS Negl Trop Dis 2019;13:e0007189.

A Girard et al. Raman spectroscopic analysis of skin as a diagnostic tool for Human African Trypanosomiasis. PLoS Pathogens 2021;17:e1010060.

J Mathu Ndung'u et al. Trypa-NO! contributes to the elimination of *gambiense* human African trypanosomiasis by combining tsetse control with "screen, diagnose and treat" using innovative tools and strategies. PLoS Negl Trop Dis 2020;14:e0008738.

F Courtin et al. Sleeping sickness in the historical focus of forested Guinea: update using a geographically based method. Parasite 2019;26:61.

CFA Compaoré et al. Analytical sensitivity of loopamp and quantitative real-time PCR on dried blood spots and their potential role in monitoring human African trypanosomiasis elimination. Exp Parasitol 2020;219:108014.

N Sima et al. SHERLOCK4HAT: a CRISPR-based tool kit for diagnosis of Human Africal Trypanosomiasis. medRxiv 2022, doi.org/10.110/2022.03.09.22271543.

https://www.cdc.gov/parasites/sleepingsickness/diagnosis.html

https://www.nibsc.org/

https://www.fda.gov/medical-devices/device-advice-comprehensive-regulatory-assistance/medical-device-databases

https://www.eptis.org/

NM Fenn Buderer. Statistical methodology : I. Incorporating the prevalence of disease into the sample size calculation for sensitivity and specificity. Acad Emerg Med 1996;3:895-900.

Acronyms

CATT	Card Agglutination Test for Trypanosomiasis
CNS	Central nervous system
CRISPR	Clustered regularly interspaced short palindromic repeats
CSF	Cerebrospinal fluid
CTC	Capillary tube centrifugation
DBS	Dry blood spot
DRC	Democratic Republic of the Congo
DTAG-NTD	Diagnostic Technical Advisory Group for Neglected Tropical Diseases
ELISA	Enzyme linked immunosorbent assay
ESAG	Expression site associated genes
EQA	External Quality Assurance
FISH	Fluorescence in situ hybridization
IHC	Immunohistochemical
ITM	Institute for Tropical Medicine
ITS	Internal transcribed spacer
IVR	Identification of villages at risk
LAMP	Loop-mediated isothermal amplification
mAECT	mini-anion exchange centrifugation technique
MTA	Material transfer agreement
NAAT	Nucleic acid amplification test
NASBA	Nucleic acid sequence based amplification

NSSCP	National sleeping sickness control programmes
PBMCs	Peripheral blood mononuclear cells
PCR	Polymerase chain reaction
POC	Point of care
PQA	WHO prequalification assessment
PSG	Polysomnographic syndrome
PSGlu	Phosphate saline glucose
RDT	Rapid diagnostic test
RIME	Repetitive insertion mobile element
SNP	Single nucleotide polymorphism
SRA	Serum resistance associated
Tbg	*Trypanosoma brucei gambiense*
TbGK	*Trypanosoma brucei* glycerol kinase
Tbr	*Trypanosoma brucei rhodesiense*
TL	Trypanolysis
QC	Quality control
VAT	Variable antigen type
VSG	Variant surface glycoprotein

FIND Diagnosis for all

Annex 1

Specifications of potential sources of biospecimens from published studies and from completed or ongoing clinical trials. Included are articles published after 2015 and clinical trials with at least 30 participants and completed after 2010.

Type of collection	Reference	Contact
2015-2021		
231 Tbg patients, 257 controls, 234 CATT positive cases from Guinea and Côte d'Ivoire<br><br>Plasma samples	doi.org/10.1371/journal.pntd.0003480	Veerle Lejon, IRD, veerle.lejon@ird.fr
739 *Tbg* patients, 619 endemic controls from WHO biobank<br><br>Plasma	doi.org/10.1093/cid/ciaa1264	Philippe Büscher, Institute of Tropical Medicine, Antwerp, pbuscher@itg.be
246 *Tbg* patients, 246 negative controls from DRC and Uganda<br><br>Plasma, whole blood	doi.org/10.1371/journal.pntd.0008168	Crispin Lumbala, Global Health Institute, Antwerp, crispinlumbala@gmail.com
169 *Tbg* patients and 1000 controls from Guinea (collected 2010-2014)<br><br>Plasma samples	doi.org/10.1051/parasite/2019066	Vincent Jamonneau, IRD, Vincent.jamonneau@ird.fr
73 stage 1, 77 stage 2, 100 controls from CAR<br><br>CSF samples	doi.org/10.1371/journal.pntd.0007631	Dennis Grab, University of the Health Sciences Bethesda, dennis.grab@usuhs.edu

36 CATT+ children, >2500 controls from Uganda<br><br>Plasma samples	doi.org/10.12688/aasopenres.12851.1	Kenneth Luryama Moi, Faculty of Medicine, Gulu University, Uganda, k.luryamamoi@gu.ac.ug
105 *Tbg* patients from Uganda<br><br>Plasma, CSF, lymph node aspirate samples	doi.org/10.1186/s13071-018-2634-x	Enock Matovu, College of Veterinary Medicine, Makerere, matovue@covab.mak.ac.ug
50 *Tbr* patients from Uganda<br><br>CSF samples	doi.org/10.1093/infdis/jix466	Jeremy Sternberg, University of Aberdeen, jsternberg@abdn.ac.uk
195 *Tbg* stage 1, 41 stage 2 patients from DRC<br><br>Plasma and CSF samples	doi.org/10.1016/S2214-109X(21)00208-4	Antoine Tarral, DNDi, gro.idnd@larrata
630 participants, *Tbg* stage 2, Blood, lymph node fluid or CSF,<br><br>DRC	https://www.clinicaltrials.gov/ct2/show/NCT00906880?recrs=ae&cond=African+Trypanosomiases&draw=2&rank=2	Johannes Blum, Swiss Tropical & Public Health Institute<br><br>Victor Kande, PNLTHA-DRC
230 participants *Tbg*<br><br>Blood, lymph node fluid or CSF,<br><br>DRC	https://www.clinicaltrials.gov/ct2/show/NCT02169557?recrs=ae&cond=African+Trypanosomiases&draw=2&rank=3	DNDi
125 children participants *Tbg*<br><br>Blood samples<br><br>DRC	https://www.clinicaltrials.gov/ct2/show/NCT02184689?recrs=ae&cond=African+Trypanosomiases&draw=2&rank=4	DNDi

400 participants *Tbg* stage 2,<br>CAR, DRC	https://www.clinicaltrials.gov/ct2/show/NCT01685827?recrs=ae&cond=African+Trypanosomiases&draw=2&rank=5	DNDi,<br>Vicror Kande, HAT National Control Program in DRC
10700 participants,<br>Whole blood, DBS samples<br>DRC, Guinea, Côte d'Ivoire	https://www.clinicaltrials.gov/ct2/show/NCT03356665?recrs=ae&cond=African+Trypanosomiases&draw=2&rank=7	Veerle Lejon, IRD
88 participants,<br>Blood and CSF samples<br>DRC	https://www.clinicaltrials.gov/ct2/show/NCT03112655?recrs=ae&cond=African+Trypanosomiases&draw=2&rank=8	Veerle Lejon, IRD
174 *Tbg* participants,<br>whole blood and DBS samples,<br>DRC, Guinea	https://www.clinicaltrials.gov/ct2/show/NCT03025789?recrs=ae&cond=African+Trypanosomiases&draw=2&rank=9	DNDi,<br>Victor Kande Betu Kumeso, Ministère de la Santé
260 *Tbg* participants,<br>Plasma samples,<br>DRC, Guinea	https://www.clinicaltrials.gov/ct2/show/NCT03087955?recrs=ae&cond=African+Trypanosomiases&draw=2&rank=10	DNDi,<br>Victor Kande Betu Kumeso, Ministère de la Santé
13700 participants,<br>DBS samples,<br>Burkina Faso, DRC, Côte d'Ivoire	https://www.clinicaltrials.gov/ct2/show/NCT04099628?recrs=ae&cond=African+Trypanosomiases&draw=3&rank=11	Veerle Lejon, IRD
280 *Tbg* patients,<br>Blood, lymph node aspirate, CSF samples	https://www.clinicaltrials.gov/ct2/show/NCT00146627?recrs=ae&cond=African+Trypanosomiases&draw=2&rank=12	Els Torreele, DNDi

50 *Tbr* participants,<br><br>Malawi, Uganda	https://www.clinicaltrials.gov/ct2/show/NCT03974178?recrs=ae&cond=African+Trypanosomiases&draw=2&rank=13	Deolinda Alves, DNDi, dalves@dndi.org<br><br>Westain T Nyirenda, Rumphi District Hospital, wnyirenda@extern.dndi.org<br><br>Anthony Eriatu, Lwala Hospital, aeriatu@extern.dndi.org
1200 *Tbg* participants,<br><br>Plasma samples,<br><br>DRC, Guinea	https://www.clinicaltrials.gov/ct2/show/NCT05256017?recrs=ae&cond=African+Trypanosomiases&draw=2&rank=16	Victor Kande Betu Ku<br><br>Mesu, Ministry of Public Health, Ministry<br>Kinshasa
1900 participants, different fever conditions including trypanosomiasis and leishmaniasis,<br><br>Whole blood, serum samples<br><br>Cambodia, DRC, Nepal, Sudan	https://www.clinicaltrials.gov/ct2/show/NCT01766830?recrs=ae&cond=African+Trypanosomiases&draw=2&rank=17	François Chappuis, University Hospital Geneva

Leishmaniasis, TSP Report

Introduction

Leishmaniasis is a vector (phlebotomine sandflies) borne disease complex, caused by the intracellular kinetoplastid protozoan parasite *Leishmania sp*., and with two major manifestations, visceral leishmaniasis (VL) and cutaneous leishmaniasis (CL). Other rarer forms include mucocutaneous leishmaniasis (MCL), diffuse cutaneous leishmaniasis (DCL) and post-kala-azar dermal leishmaniasis (PKDL), all of which together with CL are known as dermal leishmaniases. Leishmaniasis is endemic in around a hundred countries in Asia, Africa, South and Central America and southern Europe. VL is caused by *L. donovani*, *L. chagasi* and *L. infantum*, while the major agents of CL are *L. major, L. tropica, L. aethiopica* in Africa, Middle East and Central Asia, and *L. mexicana, L. amazonensis, L. braziliensis, and L. guyanensis* in the Americas. While CL and PKDL symptoms are skin localized, VL-causing species invade phagocytic cells in the liver, spleen and bone marrow and cause systemic disease. Some strains of *L. infantum* and *L. donovani* can cause CL, and in South East Asia and Sudan post-treatment *L. donovani* infection evolves towards PKDL in 5-50% of the cases.

Currently, annual incidence is in the range of 30 – 50 000 cases (information, Isra Cruz). First line drugs for VL are pentavalent antimonials, meglumine antomoniate, amphotericin B and liposomal amphotericin B, miltefosine and paromomycin. CL causes disfiguring lesions. First line drugs for CL are pentavalent antimonials, meglumine antomoniate, amphotericin B, pentamidine and paromomycin. Early diagnosis and effective treatment is critical to reduce the disease burden. The main diagnostic challenge is POC diagnosis of both VL (especially in eastern Africa) and CL. According to the 2021-2030 Road Map for NTDs, required actions include the development of

(i) **For VL**, more effective and user-friendly treatment and diagnostics, especially for East Africa. Devise less invasive and highly specific tests to measure parasite level. Develop less invasive test of cure for PKDL and VL. Design and apply strategies and tools for patient tracking.

(ii) **For CL**, affordable, more sensitive rapid diagnostic tests at species level that can be used at health centres and community levels (especially important in foci where several Leishmania species coexist).

A diagnostic challenge also remains in the diagnosis of VL or CL, in asymptomatic individuals. "Asymptomatic *Leishmania* infections" correspond to subjects, in a *Leishmania*-endemic area, testing positive by a molecular or serological test, with no signs or symptoms of the disease.

A Leishmaniasis (VL and CL) virtual biobank is expected to support findability and availability of biospecimen panels for (i) EQA programs (or equivalent inter-laboratory exercises),

reference and quality control material production (ii) method validation, including evaluation and/or PQA, and (iii) R&D other than validation of the analytical and clinical performance characteristics of a diagnostic test. Such R&D includes the identification of novel diagnostic biomarkers and/or their preliminary evaluation.

Scope

This document is intended as a description of the needs of biological materials in terms of VL and CL panels.

The scope of this document includes the needs of biospecimens for development, then validation of molecular biology (nucleic acid based) assays, and assays based on detection or measurement of analytes, other than nucleic acids, found in biological fluids (serum, plasma, whole blood or buffy coat, lymph node aspirate, urine, saliva). Assays intended to be used both in a context of detection of active localized CL (or other forms of dermal leishmaniases) and of active VL are in the scope.

The following are out of the scope of this document: microscopy-based examination assays, flow cytometry, immunohistochemistry, dermatoscopy.

Sources

The content of this report is based on information found in the **References** and consultation with the **DTAG**-NTD on CL and VL. Periodic revision can be made as information on ongoing and scheduled diagnostic development projects, funded by different donors becomes available.

Interviews with Dr Henck Schallig (University Amsterdam) and Dr Israel Cruz (Instituto Salud Carlos III) were also conducted.

Clinical and biological diagnosis of leishmaniasis

VL evolves towards visceral lesions in 2-8 months, with minimal skin symptoms. Fever, cachexia, pancytopenia, hypergammaglobulinemia, hepatosplenomegaly characterize VL. Biological diagnosis will be important in the future, in a post-elimination context, to identify asymptomatic individuals.

Test of Cure (TOC) for VL is important to evaluate treatment success after primary or relapse VL. For this purpose, when treatment failure is suspected, or in clinical trials, splenic, bone marrow or lymph node aspiration is performed at the end of treatment to assess the parasitological response. A direct (parasitological) TOC should be conducted for all VL relapse cases.

CL manifests as an open sore which leaves a scar, while DCL involves disseminated lepromatous lesions and MCL involves mucosal inflammatory lesions. Biological diagnosis is important to confirm infection before treatment.

The gold standard for the biological diagnosis of leishmaniasis is the microscopic identification of the parasites (amastigotes) in samples from lymph nodes, bone marrow, liver, spleen (for VL), and from skin lesions, such as skin scrapings, slit-skin smears or fine needle aspirates (FNA) (for CL), after staining with Leishman stain or Giemsa stain. Direct diagnosis of VL by qPCR in blood is more sensitive than microscopy.

Important biospecimen annotations

- Necessary annotations for VL: geographical origin, HIV and other co-infections, sex/age, fever, cachexia, hepatosplenomegaly, microscopy and /or culture and/or PCR results and/or serology results.
- Necessary annotations for CL: geographical origin, other co-infections, sex/age, number of lesions, size of lesions, duration of lesions, microscopy and/or culture and/or PCR results.
- Useful annotations for VL: preanalytical data (time to centrifugation/freezing/stabilization, storage temperature), complete blood count (CBC) results, hematocrit, total protein concentration, albumin concentration, HIV, tuberculosis, malaria co-infections. Treatment specifications and treatment outcome data are also useful, especially in the validation phase of TOC.
- Useful annotations for CL: HIV, preanalytical data (type of stabilizer, time to stabilization, storage temperature), presence of enlarged lymph nodes. Treatment specifications and treatment outcome data are also useful, especially in the validation phase of TOC.

Nucleic acid based (molecular biology) assays

The most frequently used targets for either VL or CL have been the 18S rRNA gene and the kDNA minicircles.

For VL, relevant sample types are whole blood (preferable to buffy coat or peripheral blood mononuclear cells (PBMCs), bone marrow aspirates, spleen aspirates, lymph node aspirates.

For CL, relevant sample types are skin scrapings, slit skin, skin biopsies, lesion swabs, fine needle aspirates. Use of skin scrapings on FTA cards prior LAMP has been described. Apart from PCR and LAMP assays, MinION nanopore sequencing has recently been proposed as portable diagnostic method for CL by sequencing (as per Imai 2018).

Critical preanalytical factors, for all types of biofluid samples, include time and temperature between collection and start of processing, time and temperature between end of processing and storage, long term storage temperature, and DNA or RNA extraction kit or method used. Other critical preanalytical factors are the type of anticoagulant and /or stabilizers for whole blood and blood derivatives (buffy coat, serum, plasma), the centrifugation conditions and use of stabilizer for urine, the type of stabilizer or buffer used for skin sample collection, the time between collection and stabilization, and the long term storage temperature.

Non nucleic acid based assays

For VL, serum antibodies is the first category of diagnostic biomarkers. Serological tests by ELISA, direct agglutination (DAT) or immunochromatography (ICT), immunofluorescent antibody test (IFAT), latex agglutination (KATEX), and dip-stick tests are available and mostly based on the rK39 antigen or whole leishmanial antigen. However, their usefulness for detection of active infection is limited, if not used with a strict definition of VL suspected case, since antibodies continue to circulate in the blood for several years after effective treatment. A limitation of RDTs lies in their lower sensitivity in Eastern Africa.

Leishmania extracellular vesicles (LEV) represent a novel source of potential diagnostic biomarkers for either VL or CL. Plasma is the sample type of interest for exosomal and/or metabolite diagnostic biomarkers.

A latex agglutination test (KAtex) based on the detection of a carbohydrate antigen in urine of VL patients has been described. Saliva may also be a sample type of interest, though very limited studies have been conducted.

An intradermal leishmanin skin test (LST) has been traditionally used as a delayed type hypersensitivity test to detect previous exposure to *Leishmania* (VL) in VL endemic areas, or as a CL diagnostic test. In principle, cellular immune response based IGRA assays could be applied in the future as a TOC.

Serology is not relevant for CL, since there is usually no systemic antibody response. Hence, serum and plasma are not relevant sample types, except maybe for MCL. Skin aspirates, collected after flushing the skin lesion and aspirating the flush fluid, represent a potential source of diagnostic biomarkers, including IgA.

For antibody-based assays (VL), critical preanalytical factors include the long-term storage conditions. For protein and/or metabolite-based assays, critical preanalytical factors include the type of anticoagulant, the time and temperature between collection and start of processing or analysis, the centrifugation conditions, the time and temperature between end of processing and cryopreservation, the long-term storage conditions. For potential tests based on LEV biomarkers, critical preanalytical factors also include (on top of the above) the number of freeze thaw cycles and the method of LEV isolation.

Potential cross reactivities (important to inform the needs for validation of specificity, as relevant, depending on the analyte):

Any long term (>2 weeks) fever causing disease, including sleeping sickness, Chagas disease, histoplasmosis, malaria, tuberculosis, paracoccidiomycosis. Specifically for CL/PKDL, leprosy can be considered.

Table 1 gives the different contexts of use of diagnostic tests and the corresponding most important sample types and method types. Bone marrow aspirate and spleen aspirate collection, although relevant in VL, is difficult to deploy in the field.

Table 1

Context of use	Sample type and method
Detection of active VL	Serum/plasma, urine, saliva, DBS (IgG/IgM ELISA or other biomarkers)<br>DBS (capillary blood), bone marrow aspirate, spleen aspirate (NAAT)
Detection of active CL (or PKDL)	Skin scrapings, slit skin, lesion swabs, skin FNA (NAAT)

Need for samples corresponding to different *Leishmania* species and strains:

The main relevant geographical area for VL and PKDL biospecimen collection are central and southern Sudan, northern Kenya, south-east Ethiopia, Uganda, Chad, Yemen, north-east India, Bangladesh, Nepal, Bhutan, China *(L. donovani*); central and western Mediterranean, north Africa, Iran, south west Asia, central and south America (*L. infantum*).

The main relevant geographical areas for CL biospecimen collection are sub-Saharan Africa, north Africa, Middle East, south west Asia, north west India, central Asia, west Africa (*L. major, L. tropica, L. aethiopica*); central and south America, southern USA, Guyanas (*L. amazonensis, L. mexicana, , L. brazilensis, L. guyanensis*).

There is high population-specific genetic diversity of *Leishmania*. Variations in the parasite genome are associated with its geographical distribution and clinical manifestations. For example, frequency and clinical outcome of PKDL differs by geographical area: while only 5% of VL cases evolve to PKDL in India, this frequency reaches 50% in eastern Africa (mainly in Sudan). Also, despite the disease manifestation specificity for each *Leishmania* species, exceptions exist, e.g. a *L. donovani* Sri Lankan variant causing CL. In addition to the role of mutations in parasite diversity, the *Leishmania* genome is highly plastic and constantly rearranges, resulting in variations in gene copy number, clusters of genes, or even whole chromosomes. MLST analyses have also shown high variability. For all these reasons, it is important to collect samples from as diverse geographical areas as possible.

Reference methods:

- Microscopy on different types of biological fluids (VL) or skin samples (CL, PKDL)

Reference materials

A WHO international standard exists for *Leishmania* species (information, Isra Cruz). A reference material for *Leishmania* is currently under development by NIBSC.

FDA reference panels

No FDA reference panel for leishmaniasis could be found.

Validated (FDA approved) methods

No FDA approved diagnostic method could be found.

Other methods that have been described/made available for VL

- DAT (AMC, Netherlads), in the WHO EDL
- IT-LEISH (Bio-Rad, USA and DiaMed, Switzerland), RDT based on recombinant K39 protein, using blood, serum or urine samples
- Kalazar Detect (InBios, USA), RDT, based on rK39, using blood or serum samples
- KAtex (Kalon Biological, UK), LAG test in urine, based on a low MW carbohydrate antigen
- CrystalKA (Span Diagnostics), based on rKE16
- Signal-KA (Span Diagnostics), based on rKE16
- On site Leishmania Ab Rapid Test (CTK Biotech), based on rK39 or rK28 antigen
- An in-house LAG test based on A2 antigen or promastigote lysate proteins (as per Akhoundi 2013)
- Different home-made IFAT and ELISA tests
- Different home-made LAMP assays
- LAMP assay (Eiken, Japan)

Other methods that have been described/made available for CL

- An ELISA based on recombinant Lb8E and Lb6H (as per Massae Sato 2017)
- Different home-made LAMP assays
- LAMP assay (Eiken, Japan)
- CL Detect Rapid Test for Cutaneous Leishmaniasis (InBios, USA)

Information on the composition of the panels that have been used for method validation of the Eiken LAMP assay has been published (ER Adams et al 2018). The panel included 24 positive and 26 negative blood samples.

Information on the composition of the panels that have been used for method validation of the CL RDT has also been published by InBios. The panel included 150 negative samples from

non-endemic population, 149 positive samples from endemic population, cross-reactivity study against 26 different bacterial species, 5 mycobacterial species, 10 fungal species, 6 Trypanosoma species, 3 viral strains.

EQA programs

The only available EQA program that could be found on the EPTIS website is a scheme based on blood microscopy (UK NEQAS).

An inter-laboratory exercise and design for *Leishmania* PCR was performed in 2013 by Cruz et al.

Panel needs

R&D / method validation needs

Table 2a shows the needs in terms of panels for initial development /feasibility studies, for both nucleic acid-based and non-nucleic acid-based assays for VL and PKDL.

The *L. donovani* samples should originate from both East Africa and South East Asia (Bangladesh, Nepal). The *L. infantum* samples should originate from both South Europe and Latin America.

Table 2a

Sample type	Quantity per donor (for 1 development panel)	Number of biospecimen donors
Serum/<br>plasma	1ml	10 *L. donovani* positive cases<br>10 *L. infantum* positive cases<br>20 *Leishmania* negative cases
Urine	2ml	10 *L. donovani* positive cases<br>10 *L. infantum* positive cases<br>20 *Leishmania* negative cases
Saliva	1ml	10 *L. donovani* positive cases<br>10 *L. infantum* positive cases<br>20 *Leishmania* negative cases
Whole blood or DBS	1ml WB or 5 DBS	10 *L. donovani* positive cases<br>10 *L. infantum* positive cases<br>20 *Leishmania* negative cases
Bone marrow aspirate*	0,5ml	10 *L. donovani* positive cases<br>10 *L. infantum* positive cases<br>20 *Leishmania* negative cases

Spleen aspirate*	0,5ml	10 *L. donovani* positive cases<br>10 *L. infantum* positive cases<br>20 *Leishmania* negative cases
Lymph node aspirate*	0,5ml	10 *L. donovani* positive cases<br>10 *L. infantum* positive cases<br>20 *Leishmania* negative cases
Specifically for PKDL,<br>Skin scrapings<br>, slit skin, lesion swabs, Or skin lesion FNA	2 samples<br>Or 0,2ml FNA	10 *L. donovani* positive cases<br>10 *L. infantum* positive cases<br>20 *Leishmania* negative cases

*not the preferred sample types

Table 2b shows the needs in terms of panels for initial development /feasibility studies, for both nucleic acid-based and non-nucleic acid-based assays for CL.

Samples should originate from as many different geographical areas as possible and correspond to different clinical presentations, both localized and disseminated.

Table 2b

Sample type	Quantity per donor (for 1 development panel)	Number of biospecimen donors
Skin scrapings	2 samples	10 *L. major* positive cases<br>10 *L. tropica* positive cases<br>10 *L. aethiopica*, *L. mexicana, L. amazonensis, L. braziliensis, L. panamesis, L. guyanensis* or other *Leishmania* sp. positive cases<br>20 *Leishmania* negative cases
Skin lesion FNA	0,2ml	10 *L. major* positive cases<br>10 *L. tropica* positive cases<br>10 *L. aethiopica*, *L. mexicana, L. amazonensis, L. braziliensis, L. panamesis, L. guyanensis* or other *Leishmania* sp. positive cases<br>20 *Leishmania* negative cases

Table 2c shows the needs in terms of complete validation panels for VL and PKDL and for both nucleic acid-based and non-nucleic acid-based assays. Numbers of donors are defined based on the draft TPP by Dr Koert Ritmeijer and on the TPP by Cruz et al. 2019.

The *L. donovani* samples should originate from both East Africa and India. The *L. infantum* samples should originate from both Mediterranean basin and Latin America.

FIND Diagnosis for all

Table 2c

Sample type	Quantity per donor (for 1 validation panel)	Number of biospecimen donors
Serum/ plasma	0,5ml	20[1] *L. donovani* positive cases, of which 10 HIV positive<br>20[1] *L. infantum* positive cases, of which 10 HIV positive<br>800[1] *Leishmania* negative cases<br>Mix of 800[1] cases, *Leishmania* negative, of which half being positive for *Trypanosoma brucei*, *Trypanosoma cruzi*, *Plasmodium sp.*, *Mycobacterium tuberculosis, Mycobacterium leprae, Histoplasma capsulatum, Paracoccidioides brasiliensis* or any infectious agent causing prolonged fever
Urine	1ml	20[1] *L. donovani* positive cases, of which 10 HIV positive<br>20[1] *L. infantum* positive cases, of which 10 HIV positive<br>800[1] *Leishmania* negative cases<br>Mix of 800[1] cases, *Leishmania* negative, of which half being positive for *Trypanosoma brucei*, *Trypanosoma cruzi*, *Plasmodium sp.*, *Mycobacterium tuberculosis, Mycobacterium leprae, Histoplasma capsulatum, Paracoccidioides brasiliensis* or any infectious agent causing prolonged fever
Saliva	0,5ml	20[1] *L. donovani* positive cases, of which 10 HIV positive<br>20[1] *L. infantum* positive cases, of which 10 HIV positive<br>800[1] *Leishmania* negative cases<br>Mix of 800[1] cases, *Leishmania* negative, of which half being positive for *Trypanosoma brucei*, *Trypanosoma cruzi*, *Plasmodium sp.*, *Mycobacterium tuberculosis, Mycobacterium leprae, Histoplasma capsulatum, Paracoccidioides brasiliensis* or any infectious agent causing prolonged fever

Whole blood or DBS	0,2ml WB or 1 DBS	20(1) *L. donovani* positive cases, of which 10 HIV positive<br>20(1) *L. infantum* positive cases, of which 10 HIV positive<br>800(1) *Leishmania* negative cases<br>Mix of 800(1) cases, *Leishmania* negative, of which half being positive for *Trypanosoma brucei*, *Trypanosoma cruzi*, *Plasmodium sp.*, *Mycobacterium tuberculosis, Mycobacterium leprae, Histoplasma capsulatum, Paracoccidioides brasiliensis* or any infectious agent causing prolonged fever
Bone marrow aspirate*	0,1ml	20(1) *L. donovani* positive cases, of which 10 HIV positive<br>20(1) *L. infantum* positive cases, of which 10 HIV positive<br>800(1) *Leishmania* negative cases<br>Mix of 800(1) cases, *Leishmania* negative, of which half being positive for *Trypanosoma brucei*, *Trypanosoma cruzi*, *Plasmodium sp.*, *Mycobacterium tuberculosis, Mycobacterium leprae, Histoplasma capsulatum, Paracoccidioides brasiliensis* or any infectious agent causing prolonged fever
Spleen aspirate*	0,1ml	20(1) *L. donovani* positive cases, of which 10 HIV positive<br>20(1) *L. infantum* positive cases, of which 10 HIV positive<br>800(1) *Leishmania* negative cases<br>Mix of 800(1) cases, *Leishmania* negative, of which half being positive for *Trypanosoma brucei*, *Trypanosoma cruzi*, *Plasmodium sp.*, *Mycobacterium tuberculosis, Mycobacterium leprae, Histoplasma capsulatum, Paracoccidioides brasiliensis* or any infectious agent causing prolonged fever
Specifically for PKDL,<br>Skin scrapings	1 sample	20(1) *L. donovani* positive cases, of which 10 HIV positive<br>20(1) *L. infantum* positive cases, of which 10 HIV positive<br>800(1) *Leishmania* negative cases<br>Mix of 800(1) cases, *Leishmania* negative, of which half being positive for *Trypanosoma brucei*, *Trypanosoma cruzi*, *Plasmodium sp.*, *Mycobacterium tuberculosis, Mycobacterium leprae, Histoplasma capsulatum,*

		Paracoccidioides brasiliensis or any infectious agent causing prolonged fever
Specifically for PKDL,<br><br>skin lesion FNA	0,1ml	20[(1)] *L. donovani* positive cases, of which 10 HIV positive<br><br>20[(1)] *L. infantum* positive cases, of which 10 HIV positive<br><br>800[(1)] *Leishmania* negative cases<br><br>Mix of 800[(1)] cases, *Leishmania* negative, of which half being positive for *Trypanosoma brucei*, *Trypanosoma cruzi*, *Plasmodium sp.*, *Mycobacterium tuberculosis, Mycobacterium leprae, Histoplasma capsulatum, Paracoccidioides brasiliensis* or any infectious agent causing prolonged fever

[1] applicable to a context of detection of active VL or PKDL (assumed prevalence inside an endemic area of 1%, expected sensitivity 100%, expected specificity 95%, desired precision 10%)
*not the preferred sample types

Table 2d shows the needs in terms of complete validation panels for CL and for both nucleic acid-based and non-nucleic acid-based assays. Numbers of donors are defined based on the TPP by Cruz et al. 2019.

Samples should originate from as many different geographical areas as possible and correspond to different clinical presentations, both topical and disseminated.

FIND Diagnosis for all

Table 2d

Sample type	Quantity per donor (for 1 validation panel)	Number of biospecimen donors
Skin scrapings	1 sample	20[(1)] *L. major* positive cases<br>20[(1)] *L. tropica* positive cases<br>20[(1)] *L. aethiopica*, *L. mexicana, L. amazonensis, L. braziliensis, L. panamesis, L. guyanensis* or other *Leishmania* sp. positive cases<br>800[(1)] *Leishmania* negative cases<br>Mix of 800[(1)] cases, *Leishmania* negative, of which half being positive for *Trypanosoma brucei*, *Mycobacterium tuberculosis, Mycobacterium leprae, Histoplasma capsulatum,* or *Paracoccidioides brasiliensis*
Skin lesion FNA	0,1ml	20[(1)] *L. major* positive cases<br>20[(1)] *L. tropica* positive cases<br>20[(1)] *L. aethiopica*, *L. mexicana, L. amazonensis, L. braziliensis, L. panamesis, L. guyanensis* or other *Leishmania* sp. positive cases<br>800[(1)] *Leishmania* negative cases<br>Mix of 800[(1)] cases, *Leishmania* negative, of which half being positive for *Trypanosoma brucei*, *Mycobacterium tuberculosis, Mycobacterium leprae, Histoplasma capsulatum,* or *Paracoccidioides brasiliensis*

[1] applicable to a context of detection of active CL (assumed prevalence inside a highly endemic area of 1%, expected sensitivity 100%, expected specificity 95%, desired precision 10%)

Positivity status for VL, as an attribute of the donor/patient is defined by a positive parasitological test (microscopic detection of *Leishmania* parasites in lymph node aspirate or other biological fluid) and/or positive NAAT.

Positivity status for CL, as an attribute of the donor/patient is defined by a positive parasitological test (microscopic detection of *Leishmania* parasites in skin scrapings) and/or positive NAAT.

Sample sizes have been estimated according to the NM Fenn Buderer statistical approach, for the desired diagnostic sensitivity and specificity levels, using PASS 2021 software at an actual significance level between 0.05 and 0.15 and with 80% power to detect a reduction in sensitivity or specificity of 10%.

Complete biospecimen sets, including all biospecimen types, from the same donor are preferred. It is critical that for a validation panel, all biospecimens come from the same collection, to avoid preanalytical bias. Preanalytical bias is unavoidable if using biospecimens from different collections with different or undocumented preanalytical specifications.

Possible retrospective sources of specimens can be found in published literature and in completed or ongoing clinical trials (Annex 1). The most suitable sources for identification and validation of stage-specific or treatment efficacy biomarkers would be **longitudinal** collections from geographically different endemic areas, with pre-and post-treatment sampling of patients.

Although the first priority is the needs for development and validation of new methods, in order for a new method to be deployed in the field, an external quality assurance (EQA) program is needed, as well as quality control (QC) materials to be included in the kits.

EQA needs

The needs for EQA panels for non-nucleic acid-based assays are listed below. Tables 3a and 3b show the minimum necessary quantities per VL and CL panel respectively, for **one EQA scheme and round** for **40 participating laboratories**, with each laboratory receiving 1 sample. The quantities correspond to the quantities to be distributed to the participating laboratories, supplemented by the quantities necessary to perform homogeneity and stability testing, and value assignment by the EQA provider.

The best retrospective source of samples would be leishmaniasis screening centers in endemic countries. The samples necessary for CL EQA cannot be produced from native biospecimens due to difficulty to collect and to lack of homogeneity. For this reason, we refer to “skin scraping like” samples that are artificial samples, spiked with *Leishmania* DNA.

Table 3a, EQA for VL

	Sample type	Quantity per item (for 1 EQA round)	Number of items
Non nucleic acid based or nucleic acid based	DBS	60 spots	3 items *L. donovani/L. infantum* positive<br>1 items *Leishmania* negative

Table 3b, EQA for CL

	Sample type	Quantity per item (for 1 EQA round)	Number of items
Nucleic acid based	"skin scraping like" on FTA cards	60 FTA card spots	3 items *L. major/L. tropica* positive<br>1 items *Leishmania* negative

Commercialized kit, QC material needs

For one kit for VL using serum or plasma,

- At least 500ml of pooled *Leishmania sp.* serum/plasma

For a NAAT kit for VL or CL, the QC material can be a solution of *Leishmania* nucleic acid.

References consulted for the VL and CL TSPs

R Reithinger et al. Cutaneous leishmaniasis. Lancet Infect Dis 2007;7:581-596.

F Chappuis et al. Visceral leishmaniasis: what are the needs for diagnosis, treatment and control? Nature Rev Microbiol 2007;5:S7-16.

https://www.who.int/health-topics/leishmaniasis#tab=tab_1

https://apps.who.int/iris/bitstream/10665/70809/1/WHO_HTM_NTD_2012.1_eng.pdf

http://apps.who.int/iris/bitstream/handle/10665/44412/WHO_TRS_949_eng.pdf;jsessionid=CBD76DC5148F50C7EA1AD18092A48C19?sequence=1

MP Barrett & SL Croft. Management of trypanosomiasis and leishmaniasis. Brit Med Bull 2012;104:175-196.

I Cruz et al. Target Product Profile for a point-of-care diagnostic test for dermal leishmaniasis. Parasite Epidemiol and Control 2019;3:e00103.

ND Karunaweera et al. Sri Lankan cutaneous leishmaniasis is caused by Leishmania donovani zymodeme MON-37. Trans R Soc Trop Med Hyg 2003;97:380–1.

B Akhoundi et al. Rapid detection of human and canine visceral leishmaniasis: assessment of a latex agglutination test based on the A2 antigen from amastigote forms of Leishmania infantum. Exp Parasitol 2013;133:307-313.

C Massae Sato et al. Use of Recombinant Antigens for Sensitive Serodiagnosis of American Tegumentary Leishmaniasis Caused by Different Leishmania Species. J Clin Microbiol 2017;55:495-503.

C Avendano et al. Loop-mediated isothermal amplification as point-of-care diagnosis for neglected parasitic infections. Int J Mol Sci 2020;21:7981.

AC Erber et al. Diagnosis of visceral and cutaneous leishmaniasis using loop-mediated isothermal amplification (LAMP) protocols: a systematic review and meta-analysis. Parasites and Vectors 2022;15:34.

AB Ibarra-Meneses et al. Identification of asymptomatic Leishmania infections, a scoping review. Parasites and Vectors 2022;15:5.

D Kumari et al. Advancement in leishmaniasis diagnosis and therapeutics, an update. Eur J Pharmacol 2021;910:174436.

RC Fortes De Brito et al. Recent advances and new strategies in leishmaniasis diagnosis. Appl Microbiol Biotechnol 2020;104:8105-8116.

J Carstens-Kass et al. A review of the leishmanin skin test. A neglected test for a neglected disease. PLoS Negl Trop Dis 2021;15:e0009531.

GS Nunes Bezerra et al. Urine as a promising sample for Leishmania DNA extraction in the diagnosis of visceral leishmaniasis – a review. Braz J Infect Dis 2019;23:111-120.

Y Taslimi et al. A novel non-invasive diagnostic sampling diagnostic for cutaneous leishmaniasis. PLoS Negl trop Dis 2017;11:e0005750.

K Imai et al. Non-invasive diagnosis of cutaneous leishmaniasis by the direct boil loop-mediated isothermal amplification method and MinION nanopore sequencing. Parasitol Int 2018;67:34-37.

Er Adams et al. Development and evaluation of a novel loop-mediated isothermal amplification assay for diagnosis of cutaneous and visceral leishmaniasis. J Clin Microbiol 2018;56:1-8.

CO Nzelu et al. A rapid molecular diagnosis of cutaneous leishmaniasis by colorimetric malachite green-loop-mediated isothermal amplification (LAMP) combined with an FTA card as a direct sampling tool. Acta Tropica 2016;153:116-119.

A Martins Gabriel et al. Leishmania 360° : Guidelines for exosomal research. Microorganisms 2021;9:2081.

N Douanne et al. Unravelling the proteomic signature of extracellular vesicles released by drug resistant *Leishmania infantum* parasites. PLoS Negl Trop Dis 2020;3:e202000742.

MA Ghatei et al. Performance of latex agglutination test (KAtex) in diagnosis of visceral leishmaniasis in Iran. Iran J Immunol 2009;6:202-207.

https://www.cdc.gov/parasites/leishmaniasis/health_professionals/index.html#dx

https://www.cdc.gov/parasites/leishmaniasis/resources/pdf/Leishmaniasis_Guide_Collection_2021.pdf

https://www.nibsc.org/

https://www.fda.gov/medical-devices/device-advice-comprehensive-regulatory-assistance/medical-device-databases

https://www.eptis.org/

I Cruz et al. An approach for interlaboratory comparison of conventional and real-time PCR assays for diagnosis of human leishmaniasis. Exp Parasitol 2013;134:281-289.

ER Adams et al. Development and evaluation of a novel loop-mediated isothermal amplification assay for diagnosis of cutaneous and visceral leishmaniasis. J Clin Microbiol 2018;56:e00386-18.

https://inbios.com/wp-content/uploads/2019/08/LBL-0057-01-900159-IVD-CL-Detect-Rapid-Test-Package-Insert.pdf

NM Fenn Buderer. Statistical methodology : I. Incorporating the prevalence of disease into the sample size calculation for sensitivity and specificity. Acad Emerg Med 1996;3:895-900.

Acronyms

CBC	Complete blood count
CL	Cutaneous leishmaniasis
CSF	Cerebrospinal fluid
DAT	Direct agglutination test
DBS	Dry blood spot
DCL	Diffuse cutaneous leishmaniasis
DRC	Democratic Republic of the Congo
DTAG-NTD	Diagnostic Technical Advisory Group for Neglected Tropical Diseases
EDL	Essential in vitro diagnostics
ELISA	Enzyme linked immunosorbent assay
EQA	External Quality Assurance
FISH	Fluorescence in situ hybridization
FNA	Fine needle aspirate
FTA	Flinders Technology Associates
ICT	Immunochromatographic test
IFAT	Immunofluorescent antibody test
IGRA	Interferon gamma release assay
IHC	Immunohistochemical
kDNA	Kinetoplast DNA minicircles
LAG	Latex agglutination
LAMP	Loop mediated isothermal amplification

LEV	*Leishmania* extracellular vesicles
LST	Leishmanin skin test
MCL	Mucocutaneous leishmaniasis
MLST	Multilocus sequence typing
MTA	Material transfer agreement
MW	Molecular weight
NAAT	Nucleic acid amplification test
NASBA	Nucleic acid sequence based amplification
PBMCs	Peripheral blood mononuclear cells
PCR	Polymerase chain reaction
PKDL	Post kala-azar dermal leishmanisasis
POC	Point of care
PQA	WHO prequalification assessment
RDT	Rapid diagnostic test
TOC	Test of cure
QC	Quality control
VL	Visceral leishmaniasis

Annex 1

Specifications of potential sources of biospecimens from published studies and from completed or ongoing clinical trials. Included are articles published after 2012 and clinical trials with at least 30 participants and completed after 2010.

Type of collection	Reference	Contact
2012-2021		
VL		
42 VL cases, 40 post treatment VL, 6 PKDL, 139 controls from India<br><br>Blood and urine samples	doi.org/10.4103/0022-3859.101378	R P Goswami Department of Tropical Medicine, School of Tropical Medicine, Kolkata, West Bengal India
590 VL patients from Sudan<br><br>Lymph node aspirate samples	doi.org/10.1111/tmi.12603	Atia Mohammed Atia, Médecins Sans Frontières-Switzerland, Sudan Mission, Gedaref, Sudan<br><br>alatiaby@yahoo.com
750 VL patients and 750 controls from India, Brazil and East Africa<br><br>Serum samples	doi.org/10.1093/cid/cis716	Jane Cunningham, WHO, cunninghamj@who.int
128 VL patients, 107 controls from Brazil<br><br>Whole blood, serum, oral fluid samples	doi.org/10.1371/journal.pone.0230610	Maria Carmen Arroyo Sanchez, Instituto de Medicina Tropical da Faculdade de Medicina, Universidade de São Paulo, arroyo@usp.br

179 VL patients and 88 controls from India<br><br>Blood samples	doi.org/10.1371/journal.pntd.0006922	Poonam Salotra, ICMR-National Institute of Pathology, Safdarjung Hospital Campus, New Delhi, poonamsalotra@hotmail.com
55 VL and 62 PKDL patients, 44 VL controls and 24 PKDL controls from India<br><br>Blood and bone marrow aspirate samples (VL)<br><br>Skin biopsy samples (PKDL)	doi.org/10.1016/j.diagmicrobio.2013.01.011	Poonam Salotra, ICMR-National Institute of Pathology, Safdarjung Hospital Campus, New Delhi,<br><br>salotra@vsnl.com
75 VL patients and 100 controls from Bangladesh<br><br>Buffy coat samples	doi.org/ 10.1186/1756-3305-5-280	Dinesh Mondal, International Centre for Diarrhoeal Disease Research, Bangladesh, din63d@icddrb.org
30 VL patients from Iran<br><br>Serum and urine samples	Iran J Immunol 2009;6:202-207	Bahador Sarkari, School of Medicine, Shiraz University of Medical Sciences, Shiraz, Iran, sarkarib@sums.ac.ir
CL		
66 VL, 67 PKDL, 10 CL patients from India<br><br>Blood and bone marrow aspirates (VL); skin biopsy samples (PKDL, CL)	doi.org/10.1186/s12879-017-2318-8	Poonam Salotra, National Institute of Pathology (ICMR), Safdarjung Hospital Campus, New Delhi, salotra@vsnl.com

192 CL patients and 213 controls from Brazil<br><br>Serum samples	doi.org/10.1128/JCM.01904-16	Hiro Goto, Instituto de Medicina Tropical de São Paulo, Universidade de São Paulo hgoto@usp.br
119 CL patients and 54 controls from Iran<br><br>Skin tape samples	doi.org/10.1371/journal.pntd.0005750	Sima Rafati, Pasteur Institute Iran, sima-rafatisy@pasteur.ac.ir
70 CL patients from Peru<br><br>Skin FTA samples	doi.org/10.1016/j.actatropica.2015.10.013	Hirotomo Kato, School of Veterinary Medicine, Hokkaido University, Sapporo, hkato@vetmed.hokudai.ac.jp
70 CL patients from Brazil<br><br>Skin press imprint smear samples	doi.org/10.4269/ajtmh.14-0160	Anastacio Sousa, ospital Sa˜o Jose´ for Infectious Diseases, Fortaleza, Ceara, aqsousa@gmail.com
67 CL patients from multiple geographic regions<br><br>DNA samples	doi.org/10.3390/tropicalmed4040135	Ineka Gow, University of Technology, Sydney, ineka.c.gow@student.uts.edu.au
88 CL patients from Brazil<br><br>DNA from skin scrapping samples	doi.org/10.1371/journal.pntd.0008750	Otacilio Cruz Moreira, Instituto Oswaldo Cruz, Fundacão Oswaldo Cruz, Rio de Janeiro, otacilio@ioc.fiocruz.br

VL		
280 participants<br>PBMC, skin biopsies<br>Sudan, Uganda, Kenya	https://clinicaltrials.gov/ct2/show/NCT04342715?recrs=ae&cond=Visceral+Leishmaniasis&draw=2&rank=4	Paul Kaye, university of York,<br>charles.lacey@hyms.ac.uk
500 participants<br>Blood, aspirates<br>Ethiopia	https://clinicaltrials.gov/ct2/show/NCT04003532?recrs=ae&cond=Visceral+Leishmaniasis&draw=2&rank=5	Amanuel Haile, Mekelle University College of Health Sciences<br>Dawit Wolday, dawwol@gmail.com
430 participants<br>Blood, plasma<br>Ethiopia, Kenya, Uganda, Sudan	https://clinicaltrials.gov/ct2/show/NCT03129646?recrs=ae&cond=Visceral+Leishmaniasis&draw=2&rank=8	Jane Mbui, KEMRI<br>Joseph Olobo, College of Health Sciences, Makerere University<br>Ahmed Musa, Institute of Endemic Diseases, Sudan<br>Rezika Mohammed, University Hospital of Gondar, Ethiopia
30 participants<br>Plasma<br>Kenya, Uganda	https://clinicaltrials.gov/ct2/show/NCT02431143?recrs=ae&cond=Visceral+Leishmaniasis&draw=3&rank=12	Rachid Juma, KEMRI
520 particiants<br>Unspecified sample types<br>Ethiopia	https://clinicaltrials.gov/ct2/show/NCT02148822?recrs=ae&cond=Visceral+Leishmaniasis&draw=2&rank=20	Estafania Custodio, Instituto de Salud Carlos III
74 participants<br>HIV+<br>Blood<br>Ethiopia	https://clinicaltrials.gov/ct2/show/NCT01360762?recrs=ae&cond=Visceral+Leishmaniasis&draw=2&rank=22	Ermias Diro, University of Gondar, Ethiopia

140 participants<br>Plasma<br>Bangladesh, India	https://clinicaltrials.gov/ct2/show/NCT03636659?recrs=ae&cond=Visceral+Leishmaniasis&draw=2&rank=24	Nagesh Meda, Aurobindo Pharma
36 PKDL participants<br>Blood, plasma	https://clinicaltrials.gov/ct2/show/NCT01975051?recrs=ae&cond=Visceral+Leishmaniasis&draw=2&rank=25	Dinesh Mondal, International Centre for Diarrhoeal Disease Research, Bangladesh
600 participants<br>Blood, plasma, parasitological samples<br>Bangladesh	https://clinicaltrials.gov/ct2/show/NCT01122771?recrs=ae&cond=Visceral+Leishmaniasis&draw=2&rank=26	Ridwanur Rahman, Shaheed Surawardy Medical College
60 participants<br>Tissue aspirates<br>Ethiopia	https://clinicaltrials.gov/ct2/show/NCT02011958?recrs=ae&cond=Visceral+Leishmaniasis&draw=2&rank=29	Ermias Diro, University of Gondar
80 PKDL participants<br>Plasma, DNA samples<br>Bangladesh	https://clinicaltrials.gov/ct2/show/NCT02193022?recrs=ae&cond=Visceral+Leishmaniasis&draw=2&rank=30	International Centre for Diarrheal Disease Research, Dhaka, Bangladesh
700 participants<br>Spleen, bone marrow, lymph node aspirates, blood<br>Ethiopia, Kenya	https://clinicaltrials.gov/ct2/show/NCT03646981?recrs=ae&cond=Visceral+Leishmaniasis&draw=2&rank=32	Israel Cruz, FIND
1900 participants<br>Serum, plasma, blood<br>Cambodia, Nepal, Sudan, DRC	https://clinicaltrials.gov/ct2/show/NCT01766830?recrs=ae&cond=Visceral+Leishmaniasis&draw=2&rank=46	François Chappuis, University Hospital Geneva

30 participants<br>PBMC<br>France	https://clinicaltrials.gov/ct2/show/NCT03303898?recrs=ae&cond=Visceral+Leishmaniasis&draw=2&rank=39	CHU de Nice
CL		
200 participants<br>Skin biopsies, skin slits, blood, plasma, PBMC<br>Ethiopia	https://clinicaltrials.gov/ct2/show/NCT05332093?recrs=ae&type=PReg&cond=Cutaneous+Leishmaniasis&draw=2&rank=1	Wim Adriaensen, Institute of Tropical Medicine, Antwerp<br>Annisa Befekadu Tesfaye, University of Gondar
350 participants<br>skin slit smear, dental broach, tape samples<br>Ethiopia	https://clinicaltrials.gov/ct2/show/NCT03837431?recrs=ae&type=Obsr&cond=Cutaneous+Leishmaniasis&draw=2&rank=2	Johan van Griensven, Institute of Tropical Medicine, Antwerp, jvangriensven@itg.be<br>Rezika Mohammed, College of Medicine and Health Sciences, University of Gondar, rezikamohammed@yahoo.com
270 participants<br>Skin samples (dental broach) and DNA<br>Afghanistan	https://clinicaltrials.gov/ct2/show/NCT03435419?recrs=ae&type=Obsr&cond=Cutaneous+Leishmaniasis&draw=2&rank=3	HealthNet, TPO<br>Israel Cruz, FIND
120 participants<br>Skin smears<br>Colombia	https://clinicaltrials.gov/ct2/show/NCT04500873?recrs=ae&type=Obsr&cond=Cutaneous+Leishmaniasis&draw=2&rank=4	Centro Internacional de Entrenamiento e Investigaciones Médicas, University of Texas
110 participants<br>Serum, skin biopsies, skin tape discs, skin slit smear samples	https://clinicaltrials.gov/ct2/show/NCT04699383?recrs=ae&type=Obsr&cond=Cutaneous+Leishmaniasis&draw=2&rank=5	Saskia van Henten, Inst. Of Tropical Medicine, Antwerp, svanhenten@itg.be

FIND Diagnosis for all

Ethiopia		Seid Getahun Abdala, seidgech014@gmail.com
94 participants<br>Skin slit smears,	https://clinicaltrials.gov/ct2/show/NCT04004754?recrs=ae&type=Obsr&cond=Cutaneous+Leishmaniasis&draw=2&rank=6	Johan van Griensven, Institute of Tropical Medicine, Antwerp
1600 participants<br>Skin lesion samples, blood<br>Mali	https://clinicaltrials.gov/ct2/show/NCT00344084?recrs=ae&type=Obsr&cond=Cutaneous+Leishmaniasis&draw=2&rank=7	Rick M Fairhurst, NIAID
200 participants<br>Skin smear samples<br>Morocco	https://clinicaltrials.gov/ct2/show/NCT02979002?recrs=ae&type=Obsr&cond=Cutaneous+Leishmaniasis&draw=2&rank=8	Issam Bennis, National School of Public Health - Rabat
44 participants<br>Skin samples<br>French Guyana	https://clinicaltrials.gov/ct2/show/NCT04888130?recrs=ae&type=Obsr&cond=Cutaneous+Leishmaniasis&draw=2&rank=9	DEMAR PIERRE Magalie, Centre Hospitalier de Cayenne
150 participants<br>Skin scrapings<br>USA	https://clinicaltrials.gov/ct2/show/NCT01865032?recrs=ae&type=Obsr&cond=Cutaneous+Leishmaniasis&draw=2&rank=10	Mark Lebwohl, Icahn School of Medicine at Mount Sinai
170 participants<br>Skin scrapings, skin samples with dental bross<br>Tunisia	https://clinicaltrials.gov/ct2/show/NCT01769612?recrs=ae&type=Obsr&cond=Cutaneous+Leishmaniasis&draw=2&rank=11	Afif Ben Salah, Institut Pasteur de Tunis

FIND Diagnosis for all

LYMPHATIC FILARIASIS, TSP Report

Introduction

The causative agents of lymphatic filariasis (LF) include the mosquito-borne filarial nematodes *Wuchereria bancrofti, Brugia malayi, Brugia timori.* An estimated 90% of LF cases are caused by *W. bancrofti* (bancroftian filariasis). The adult worms live in the human lymphatic system and cause lymphatic dysfunctions and lymphoedemas, though only a third of infected people develop symptoms. Triple therapy treatment is based on diethylcarbamazine (DEC), albendazole and ivermectin, however massive drug administration (MDA) should be done carefully in regions with co-endemic loiasis and/or onchocerciasis, because of dangerous side effects. Although microfilariae may be cleared, live adult worms may still persist. The WHO has launched the Global Program to Eliminate Lymphatic Filariasis (GPELF).

A *Lymphatic Filariasis* virtual biobank is expected to support findability and availability of biospecimen panels for (i) EQA programs (or equivalent inter-laboratory exercises), reference and quality control material production (ii) method validation, including evaluation and /or PQA, and (iii) R&D other than validation of the analytical and clinical performance characteristics of a diagnostic test. Such R&D includes the identification of novel diagnostic biomarkers and/or their preliminary evaluation.

Scope

This document is intended as a description of the needs of biological materials in terms of Lymphatic Filariasis panels.

The scope of this document includes the needs of biospecimens for development, then validation of molecular biology (nucleic acid-based) assays and assays based on detection or measurement of analytes, other than nucleic acids, found in biological fluids (serum, plasma, whole blood). Assays intended to be used both in a context of MDA stopping and in a context of surveillance for recrudescence are in the scope. *Wuchereria bancrofti, Brugia malayi, Brugia timori* are the primary focus of this document.

The following are out of the scope of this document: morphological assays (microscopy based), ultrasound or POCUS protocols.

Sources

The content of this report is based on information found in the **References**. Periodic revision can be made as information on ongoing and scheduled diagnostic development projects, funded by different donors becomes available.

Biological diagnosis of lymphatic filariasis

The standard method for diagnosing active infection is the identification of microfilariae in a blood smear by microscopic examination. The microfilariae that cause lymphatic filariasis circulate in the blood at night (nocturnal periodicity), with the only exception of a "Pacific variant" of *W. bancrofti,* with diurnal periodicity. Blood collection should be done at night to coincide with the appearance of the microfilariae, and a thick smear should be made and stained with Giemsa or hematoxylin and eosin. For increased sensitivity, concentration techniques can be used.

Other direct methods of parasite detection are (i) ultrasonography using a 7.5 or 10 MHz probe that has allowed laboratories to visualise the movements of living adult filarial worms of *W. bancrofti* in the scrotal lymphatics of asymptomatic males with microfilaraemia, (ii) lymphoscintigraphy that has shown that even in the early, clinically asymptomatic stage of the disease, there are lymphatic abnormalities in the affected limbs of people harboring microfilariae.

Whenever possible, specimens should be collected before treatment is initiated. Since the parasitemia may fluctuate, multiple smears might be needed. These can be taken at 8 to 12 hour intervals for 2 to 3 days.

Although microscopy-based assays are not in the scope of this document, traditional microscopy for microfilariae is still considered as a reference method and therefore biospecimen annotation with microscopy data is important.

Important biospecimen annotations

- Necessary annotation for *Wuchereria bancrofti, Brugia malayi, Brugia timori*: blood smear examination-based microfilaria counting results.
- Useful annotation for *Wuchereria bancrofti, Brugia malayi, Brugia timori*: time of collection.

Nucleic acid based (molecular biology) assays

PCR tests are of high specificity and sensitivity, and detect parasite DNA in humans as well as vectors in both bancroftian and brugian filariasis. PCR assays, performed on whole blood/DBS, have species specificity, although intra-species genetic variation exists. Some efforts have been made to use urine as matrix, but so far without success. Specific amplification targets include ITS or larval transcript 2 gene, the *Hha*I repeat region for *B. malayi* or *B. timori*, the Long Dispersed Repeat (LDR1) for *W. bancrofti.* Interestingly, a NINA version of LAMP has been described (Poole 2012).

If we are interested in nucleic acid-based biomarkers, indicative of viable microfilariae or worms, then mRNA could be envisaged. However, in this case, blood would have to be collected in an appropriate RNA stabilizing solution (e.g. PAXgene RNA tube) or the time

between collection and processing or between collection and analysis would have to be shorter than 3 hours.

Critical preanalytical factors include the time of day, the presence of anticoagulant or stabilizer in the blood collection tube, the blood pre-centrifugation time and temperature (time to centrifugation; for biomarkers other than DNA), the serum/plasma freezing temperature, the freeze-thaw cycles (for biomarkers other than DNA) and the nucleic acid extraction kit or method used.

As a sidenote, PCR in blood is generally specific and sensitive enough to be used not only for diagnosis, but also for monitoring response to treatment.

Non nucleic acid based (e.g. serological) assays

Serological assays for antibodies in serum/plasma/DBS cannot differentiate between the three species *Wuchereria bancrofti, Brugia malayi, Brugia timori.* Patients with active filarial infection typically have elevated levels of antifilarial IgG4 in the blood. Anti-filarial antibodies can also be detected in urine. These assays do not unequivocally discriminate between active and past infection, nor do they generally have therapeutic predictive value. They can be used as a marker of exposure.

Examples of kits include:

- SD Bioline LF IgG4 rapid test (Wb123) – (from Standard Diagnostics, now Abbott), specific for *W. bancrofti*
- SD Bioline Oncho/LF IgG4 biplex rapid test (Ov16/Wb123) – (from Standard Diagnostics, now Abbott), specific for *W. bancrofti* and *O. volvulus*
- Filaria Detect anti-Wb123 human IgG4 ELISA (from Inbios International), specific for *W. bancrofti*
- Filariasis CELISA test (BmM14) (from Cellabs, Australia), specific for *B. malayi*
- Brugia Rapid Test (from Reszon Diagnostics), specific for *Brugia malayi, Brugia timori*. The IgG4 detected by this assay might be used as indicator of successful treatment.
- PanLF Rapid Test (from Reszon Diagnostics), specific for *Wuchereria bancrofti, Brugia malayi, Brugia timori*

More recently, a Luminex-based multiplex assay for LF and malaria has been described (Plucinski 2018).

Serological assays for antigens, present in serum/plasma, but mostly whole blood/DBS, have only been developed for *W. bancrofti.* No such test exists for *Brugia* filariasis. Serology testing of peripheral blood for microfilariae detection should consider the periodicity of the microfilariae. New techniques for antigen detection include immunochromatographic tests (ICT), which are highly sensitive and specific, and are available for the diagnosis of *W. bancrofti* infection. With this test, the parasite antigens can be detected in less than 10

minutes, independently of the microfilariae's periodicity, but most of these tests are not commercially available.

Kits include:

- TropBio Og4C3 Ag ELISA test (TropBio, Australia)
- TropBio Filariasis Antigen II ELISA (TropBio, Australia)
- BinaxNow Filariasis ICT Test (Alere, USA), an RDT
- **ALERE Filariasis Test Strip (from Abbott)**, an RDT

ALERE (ABBOTT) FTS assay on DBS samples performance validation was based on a panel of 124 Wb positive, 379 Wb negative, and 132 Wb negative/other filaria positive samples for cross reactivity assessment. This gives an idea of the type of panel that has been used to evaluate a commercial kit.

Potential serologic cross reactivities (important to inform the needs for validation of specificity, as relevant, depending on the analyte):

- When measuring antibodies, cross reactivity exists between the three lymphatic filarial species. Cross reactivity may exist against *O. volvulus, Loa loa, Mansonella perstans, Mansonella ozzardi, Schistosoma mansoni,* ***Strongyloides stercoralis***, *Taenia solium, Toxocara spp., Ascaris lumbricoides, Hymenolepsis nana, Entamoeba spp., Trichuris spp., Giardia intestinalis, Iodamoeba butschii*
- When measuring CFA, cross reactivities are essentially unknown, as these would depend on the specificity of the capture monoclonal antibodies. We do know that the BinaxNOW ICT and the Alere FTS show cross reactivity with *Loa loa* microfilariae in *L. loa* high density backgrounds.

Table 1 gives the different contexts of use of diagnostic tests and corresponding sample types and method types.

Table 1

Context of use	Sample type and method
MDA stopping,<br>*Wuchereria bancrofti, Brugia malayi, Brugia timori*	Anticoagulated or non-anticoagulated whole blood/DBS (immunoenzymatic method for microfilarial/worm antigen or other biomarker)<br>Whole blood/DBS (NAAT for microfilarial/worm mRNA or other biomarker of live parasite)

Surveillance for recrudescence,<br><br>*Wuchereria bancrofti, Brugia malayi, Brugia timori*	Non-anticoagulated whole blood/DBS, serum, plasma (immunoenzymatic method for antibodies)<br><br>Anticoagulated or non-anticoagulated whole blood/DBS (immunoenzymatic method for microfilarial antigen or other biomarker)<br><br>Whole blood/DBS (NAAT for microfilarial DNA)

Need for samples corresponding to different *Wuchereria bancrofti, Brugia malayi, Brugia timori* strains:

Relevant geographical areas for biospecimen collection, for *Wuchereria bancrofti* are tropical and subtropical areas in sub-saharan Africa, Caribbean, Latin America and South and South-East Asia; for *Brugia malayi,* tropical areas in South and South-East Asia, such as China, India (Kerala), Indonesia (South and Central Sulawesi), Korea, Malaysia (Penang) and; for *Brugia timori*, the Timor, Flores and Alor islands.

There is ample evidence of high genetic diversity among *W. bancrofti* and *B. malayi* strains from different geographical areas, based on microsatellite markers, RAPD or mitochondrial genome sequencing, and there remains a need for better characterization of lymphatic microfilariae by sequencing in whole blood samples. Therefore, collection of samples from different geographical areas is desirable.

Reference methods:

- Both the microscopic microfilarial examination and the specific circulating filarial antigen detection or measurement are considered as "gold standard".

Reference materials

No WHO international standard exists for LF.

B. malayi is the only lymphatic filarial parasite that can be maintained in a laboratory and therefore the only one for which laboratory production of a reference material is feasible.

FDA reference panels

No FDA reference panel for LF could be found.

Validated (FDA approved) methods

- No FDA approved method could be found.

Other methods that have been made/are available

Apart from the assays listed above,

- ELISA tests are available from Abcam, MyBioSource and Aviva Systems.

No information on the composition of the panels that have been used for method validation by the companies could be found for any of these ELISA tests.

<u>EQA programs</u>

No commercially available EQA program for any parasitic disease and methods other than microscopy, except for toxoplasmosis, could be found on the CDC site.

On the EPTIS website, no EQA program is listed for LF, and no publication of any academic inter-laboratory exercise could be found.

Panel needs

R&D / method validation needs

No data from any FDA submission that could be used to inform us on number of reference positive samples used for sensitivity assessment, number of reference negative samples for specificity assessment, number and type of samples for cross reactivity assessment, could be found.

Table 2a shows the needs in terms of panels for initial development /feasibility studies, for both nucleic acid-based and non-nucleic acid-based assays.

Table 2a

Sample type	Quantity per donor (for 1 development panel)	Number of biospecimen donors
Serum/ plasma	0,5-1ml[1]	30 *Wuchereria bancrofti* positive cases<br>30 *Brugia malayi* positive cases<br>30 *Brugia timori* positive cases<br>30 *Wuchereria bancrofti/ Brugia malayi/Brugia timori* negative cases[2]

		30 *Wuchereria bancrofti/ Brugia malayi/Brugia timori* negative from *O. volvulus* positive cases[3]
Urine	2ml	30 *Wuchereria bancrofti* positive cases<br>30 *Brugia malayi* positive cases<br>30 *Brugia timori* positive cases<br>30 *Wuchereria bancrofti/ Brugia malayi/Brugia timori* negative cases[2]<br>30 *Wuchereria bancrofti/ Brugia malayi/Brugia timori* negative from *O. volvulus* positive cases[3]
Whole blood or DBS	0,4ml WB or 2 DBS	30 *Wuchereria bancrofti* positive cases<br>30 *Brugia malayi* positive cases<br>30 *Brugia timori* positive cases<br>30 *Wuchereria bancrofti/ Brugia malayi/Brugia timori* negative cases[2]<br>30 *Wuchereria bancrofti/ Brugia malayi/Brugia timori* negative from *O. volvulus* positive cases[3]

[1]At least one sample, in 1ml amount

[2]For information, a diagnostic developer working on serological rapid tests, uses 80 negative samples from healthy donors

[3]Samples from *O. volvulus* cases, collected in areas that are not endemic for LF

Table 2b shows the needs in terms of complete validation panels for each indication and for both nucleic acid-based and non-nucleic acid-based assays.

Numbers of samples based on the WHO TPP. Sample sizes have been estimated according to the NM Fenn Buderer statistical approach, for the desired diagnostic sensitivity and specificity levels, using PASS 2021 software at an actual significance level between 0.05 and 0.15 and with 80% power to detect a reduction in sensitivity or specificity of 10%.

Table 2b

Sample type	Quantity per donor (for 1 validation panel)	Number of biospecimen donors
Serum/ plasma	0,5ml	90(1)/70(2)/20(3) *Wuchereria bancrofti positive cases*<br>90(1)/70(2)/20(3) *Brugia malayi positive cases*<br>90(1)/70(2)/20(3) *Brugia timori positive cases*
		Plus, 4200(1)/3000(2)/250(3) cases, *Wuchereria bancrofti/ Brugia malayi/Brugia timori* negative/and positive for any of the following, *O. volvulus, Loa loa, Mansonella perstans, Mansonella ozzardi, Schistosoma mansoni, **Strongyloides stercoralis**, Taenia solium, Toxocara spp., Ascaris lumbricoides, Hymenolepsis nana, Entamoeba spp., Trichuris spp., Giardia intestinalis, Iodamoeba butschii*
Urine	1ml	90(1)/70(2)/20(3) *Wuchereria bancrofti* positive cases<br>90(1)/70(2)/20(3) *Brugia malayi* positive cases<br>90(1)/70(2)/20(3) *Brugia timori* positive cases
		Plus, 4200(1)/3000(2)/250(3) cases, *Wuchereria bancrofti/ Brugia malayi/Brugia timori* negative/and positive for any of the following, *O. volvulus, Loa loa, Mansonella perstans, Mansonella ozzardi, Schistosoma mansoni, **Strongyloides stercoralis**, Taenia solium, Toxocara spp., Ascaris lumbricoides, Hymenolepsis nana, Entamoeba spp., Trichuris spp., Giardia intestinalis, Iodamoeba butschii*
Whole blood or DBS	0,2ml WB or 1 DBS	90(1)/70(2)/20(3) *Wuchereria bancrofti* positive cases<br>90(1)/70(2)/20(3) *Brugia malayi* positive cases<br>90(1)/70(2)/20(3) *Brugia timori* positive cases
		Plus, 4200(1)/3000(2)/250(3) cases, *Wuchereria bancrofti/ Brugia malayi/Brugia timori* negative/and positive for any of the following, *O. volvulus, Loa loa, Mansonella perstans, Mansonella ozzardi, Schistosoma mansoni, **Strongyloides stercoralis**, Taenia solium, Toxocara spp., Ascaris lumbricoides, Hymenolepsis nana,*

		Entamoeba spp., Trichuris spp., Giardia intestinalis, Iodamoeba butschii

[1] applicable to a "decision making for stopping MDA" context, as a "single test" approach (assumed prevalence 2%, expected sensitivity 60%, expected specificity 99.7%, desired precision 10%). Collection should be from a region under endemic conditions.
[2] applicable to a "decision making for stopping MDA" context, as a "decision confirmatory test" approach (assumed prevalence 2%, expected sensitivity 85%, expected specificity 96%, desired precision 10%). Collection should be from a region under endemic conditions.
[3] applicable to a surveillance context of use (assumed prevalence 5%, expected sensitivity 99%, expected specificity 99.8%, desired precision 10%). Collection should be from a region under elimination conditions.

Positivity status is an attribute of the donor. Positivity has been traditionally defined by positive direct parasitological morphological assays with monomorphic results by a reference method, applied to at least one relevant biospecimen type (whole blood) from the donor. This definition of positivity can be applied to future diagnostic tests in the context of surveillance for recrudescence, where early exposure biomarkers are needed. For future diagnostic tests, in the context of MDA stopping, positivity for *Wuchereria bancrofti/ Brugia malayi/Brugia timori* should be defined by presence of live worms. This might be achieved by worm-specific mRNA assays or other methods.

Complete biospecimen sets, including all biospecimen types, from the same donor are preferred. It is critical that for a validation panel, all biospecimens come from the same collection, to avoid preanalytical bias. Preanalytical bias is unavoidable if using biospecimens from different collections with different or undocumented preanalytical specifications.

Possible retrospective sources of such specimens can be found in published literature and in completed or ongoing clinical trials (Annex 1). The most suitable sources, especially relative to *Wuchereria bancrofti/ Brugia malayi/Brugia timori* positivity status based on live worms, would be **longitudinal** collections, including both pre-treatment and post-treatment (or during MDA) samples.

Although the first priority is the needs for development, then for validation of new methods, in order for a new method to be deployed in the field, an external quality assurance (EQA) program is needed, as well as quality control (QC) materials to be included in the kits.

EQA needs

The needs for EQA panels for nucleic acid based and non-nucleic acid-based assays are listed below. Table 3 shows the minimum necessary quantities per panel for **one EQA scheme and round** for **40 participating laboratories**, with each laboratory receiving 0,1ml serum/plasma,

and/or 1 DBS. The quantities correspond to the quantities to be distributed to the participating laboratories, supplemented by the quantities necessary to perform homogeneity and stability testing, and value assignment by the EQA provider.

The current best source of historical samples would be LF screening centers in endemic countries.

Table 3

	**Sample type**	**Quantity per item (for one EQA round)**	**Number of items**
Non nucleic acid based	Serum (or plasma), antibody-based, or filarial antigen-based	6ml*	3 items *W. bancrofti* positive<br>1 item *B.malayi* or *B. timori* positive<br>2 items LF negative
	DBS, antibody-based, or filarial antigen-based	60 spots	3 items *W. bancrofti* positive<br>1 item *B.malayi* or *B. timori* positive<br>2 items LF negative
Nucleic acid based	DBS	60 spots	3 items *W. bancrofti* positive<br>1 item *B.malayi* or *B. timori* positive<br>2 items LF negative

** can be pooled material*

Commercialized kit, QC material needs

For an antibody detection kit, depending on the kit specifications in terms of target and matrix, one of the following may apply

- At least 500ml of pooled *W. bancrofti* serum/plasma, or
- At least 500ml of pooled *B. timori or B. malayi* serum/plasma

For antigen detection kits, an antigen solution can be used (no need for biological QC materials).

References consulted for the Lymphatic Filariasis TSP

EA Ottesen & J Horton. Setting the stage for a global programme to eliminate lymphatic filariasis: the first 125 years (1875-2000). International Health 2021;13, Suppl 1:S3-S9.

https://globalhealthprogress.org/collaboration/global-alliance-to-eliminate-lymphatic-filariasis-gaelf/

RM Maizels & A Kurniawan-Atmadja. Variation and polymorphism in helminth parasites. Parasitology 2002;125:S25-S37.

ST Small et al. Molecular epidemiology, phylogeny and evolution of the filarial nematode *Wuchereria bancrofti*. Infect Genet Evol 2014;0:33-43.

SN McNulti et al. Inter and intra-specific diversity of parasites that cause lymphatic filariasis. Infect Genet Evol 2013;14:137-146.

V Pandey et al. Antigen detection assay with parasite specific monoclonal antibodies for diagnosis of lymphatic filariasis. Clin Chim Acta 2011;412:1867-1873.

GJ Weil et al. The ICT filariasis test: a rapid format antigen test for diagnosis of bancroftian filariasis. Parasitol Today 1997;13:401-404.

GJ Weil et al. Laboratory and field evaluation of a new rapid test for detecting Wuchereria bancrofti antigen in human blood. Am J Trop Med Hyg 2013;89:11–15.

A Alhassan et al. Expanding the MDx toolbox for filarial diagnosis and surveillance. Trends Parasitol 2015;31:391-400.

K Gaas et al. A multicenter evaluation of diagnostic tools to define endpoints for programs to eliminate bancroftian filariasis. PLoS Negl Trop Dis 2012;6:e1479.

CB Poole et al. Diagnosis of brugian filariasis by loop-mediated isothermal amplification. PLoS Negl Trop Dis 2012;6:e1948.

MM Plucinski et al. Multiplex serology for impact evaluation of bed net distribution on burden of lymphatic filariasis and four species of human malaria in northern Mozambique. PLoS Negl Trop Dis 2018;12:1-19.

SD Pion et al. Positivity of antigen tests used for diagnosis of lymphatic filariasis in individuals without *Wuchereria bancrofti* infection but with high *Loa loa* microfilaremia. Am J Trop Med Hyg 2016;95:1417-1423.

R Noordin et al. Multicentre evaluations of two new rapid IgG4 tests (WB rapid and panLF rapid) for detection of lymphatic filariasis. Filaria Journal 2007;6:9.

MA Rahman et al. A surveillance system for lymphatic filariasis after its elimination in Sri Lanka. Parasitol Int 2019;68:73-78.

SD BIOLINE Oncho/LF IgG4 Biplex Technical File, 2016

ALERE Filariases Test Strip Technical Note

https://www.ntdsupport.org/sites/default/files/uploads/docs/resources/ICT_FINAL_04March2014.pdf

https://www.who.int/publications/i/item/9789240018624

https://www.who.int/publications/i/item/9789240018648

https://www.ntdsupport.org/cor-ntd/ntd-connector/evaluation-rapid-diagnostic-test-prototypes-detecting-wb123-antibodies

https://www.cdc.gov/parasites/lymphaticfilariasis/

https://www.nibsc.org/products/brm_product_catalogue/who_standards.aspx

https://www.accessdata.fda.gov/scripts/cdrhdevicesatfda/index.cfm

https://www.biocompare.com/pfu/110627/soids/350163/ELISA_Kit/Schistosoma

https://www.eptis.org/

https://www.who.int/publications/i/item/9789240018648

https://www.who.int/publications/i/item/9789240018624

NM Fenn Buderer. Statistical methodology : I. Incorporating the prevalence of disease into the sample size calculation for sensitivity and specificity. Acad Emerg Med 1996;3:895-900.

Acronyms

CFA	Circulating filarial antigen
DBS	Dry blood spot
DEC	Diethylcarbamazine
DTAG-NTD	Diagnostic Technical Advisory Group for Neglected Tropical Diseases
ELISA	Enzyme linked immunosorbent assay
EQA	External Quality Assurance
FTS	Filariasis test strip
GPELF	Global Program to Eliminate Lymphatic Filariasis
ICT	Immunochromatographic card test
LAMP	Loop mediated isothermal amplification
LF	Lymphatic filariasis
MDA	Massive drug administration
MTA	Material transfer agreement
NAAT	Nucleic acid amplification test
NINA	Non instrumented nucleic acid amplification
POC	Point of care
POCUS	Point of care ultrasound
PQA	WHO prequalification assessment
RAPD	Random amplified polymorphic DNA
RDT	Rapid diagnostic test

Annex 1

Specifications of potential sources of biospecimens from published studies and from completed or ongoing clinical trials. Included are articles published after 2015 and clinical trials with at least 100 participants and completed after 2010. In certain clinical trials, specimen types that are irrelevant to LF may be reported; this is because certain clinical trials had a multiple scope, combining LF and another NTD, such as onchocerciasis.

Type of collection	Reference	Contact
250 donors from India<br><br>Serum samples	doi: 10.1016/j.intimp.2020.106431	Hoti Sugerappa Laxmanappa, Indian Council of Medical Research<br><br>sjhoti@yahoo.com
110 donors from Brasil<br><br>Serum samples	doi: 10.1590/0074-02760170435	Andre Filipe Pastor, Fundaçao Oswaldo Cruz-Fiocruz, Instituto Aggeu Magalhaes,<br><br>Andrefilipe.pastor@gmail.com
100 donors from Thailand<br><br>Serum samples	doi: 10.1017/S0022149X14000522	S. Wongkamchai, Dpt of Parasitology, Faculty of Medicine, Siriraj Hospital, Bangkok, sirichit.won@mahidol.ac.th
2300 children from Sri Lanka<br><br>Urine samples	doi: 10.1016/j.parint.2018.10.003	Makoto Itoh, Dpt of Parasitology, Faculty of Medicine, University of Jaffna, Sri Lanka, macitoh@aichi-med-u.ac.jp
14400 donors from Cameroon<br><br>DBS samples	doi: 10.1186/s12879-020-05009-3	Kebede Deribe, Centre for global health research, Brighton and Sussex Medical School, UK, kebededeka@yahoo.com

1800 donors from Cameroon<br><br>DBS samples	doi: 10.4269/ajtmh.16-0547	Sebastien Pion, IRD, Montpellier, sebastien.pion@ird.fr
250 donoers from Brasil<br><br>Serum samples	doi: 10.26633/RPSP.2021.87	Paula Fernanda Melo, Universidade Federal de Pemambuco, pfalcantara@cpqam.fiocruz.br
400 donors from Camerron<br><br>Plasma samples	doi: 10.1186/s12879-015-1317-x	Raceline Gounoue-Kamkumo, Centre for Research on Filariasis and other Tropical Diseases, Yaounde, gounoue@crfilmt.org
33000 donors from Togo<br><br>DBS samples (only 20 positive!)	10.1186/s13071-018-2843-3	Monique Ameyo Dorkenoo, Université de Lomé, monicadork@yahoo.fr
2600 children from Togo<br><br>DBS samples (and some urine and stool samples)	10.1186/s13071-020-04535-y	Ameyo Monique Dorkenoo, Faculté des Sciences de la Santé Université de Lomé, monicadork@yahoo.fr
5000 donors from Cameroon<br><br>DBS samples	doi: 10.1371/journal.pntd.0007192	Samuel Wanji, Dptment of Microbiology and Parasitology, University of Buea, swanji@yahoo.fr
190 participants, probably serum and DBS samples, Cote d'Ivoire	https://clinicaltrials.gov/ct2/show/study/NCT02974049?recrs=ae&cond=lymphatic+filariasis&draw=2&rank=1	Christopher King, Case Western Reserve University
20000 participants, DBS samples, Fiji, Haiti, India,	https://clinicaltrials.gov/ct2/show/NCT03352206?recrs=ae&cond=lymphatic+filariasis&draw=2&rank=3	Gary Weil, Georgetown University School of Medicine gary.j.weil@wustl.edu

Indonesia, Papua New Guinea		
17000 participants, DBS and stool samples, Indonesia	https://clinicaltrials.gov/ct2/show/NCT01905423?recrs=ae&cond=lymphatic+filariasis&draw=2&rank=7	Peter Fischer and Gary Weil, Geargetown University School of Medicine gary.j.weil@wustl.edu
3200 participants, DBS and stool samples, Papua New Guinea	https://clinicaltrials.gov/ct2/show/NCT03268252?recrs=ae&cond=lymphatic+filariasis&draw=2&rank=8	Christopher King, University Hospitals Cleveland Medical Center
14000 participants, DBS, stool and skin snip samples, Cote d'Ivoire	https://clinicaltrials.gov/ct2/show/NCT02032043?recrs=ae&cond=lymphatic+filariasis&draw=2&rank=13	Gary Weil, Georgetown University School of Medicine gary.j.weil@wustl.edu
180 participants, probably DBS samples, Papua New Guinea	https://clinicaltrials.gov/ct2/show/NCT01975441?recrs=ae&cond=lymphatic+filariasis&draw=2&rank=15	Peter Siba, Papua New Guinea Institution for Medical Research
4700 particpants, DBS samples, Fiji	https://clinicaltrials.gov/ct2/show/NCT03177993?recrs=ae&cond=lymphatic+filariasis&draw=2&rank=16	Andrew Steer, Murdoch Children's Research Institute; Gary Weil, Georgetown University School of Medicine
3700 participants, DBS samples, Ghana	https://clinicaltrials.gov/ct2/show/NCT03131401?recrs=ae&cond=lymphatic+filariasis&draw=2&rank=25	Daniel Boakye, Noguchi Memorial Institute for Medical Research
10000 participants, DBS samples, Papua New Guinea	https://clinicaltrials.gov/ct2/show/NCT04124250?recrs=ae&cond=lymphatic+filariasis&draw=2&rank=30	Christopher King, University Hospitals Cleveland Medical Center cxk21@case.edu
4000 participants, serum and stool samples, India	https://clinicaltrials.gov/ct2/show/NCT01547884?recrs=ae&cond=lymphatic+filariasis&draw=2&rank=38	Thomas Nutman, NIAID tnutman@mail.nih.gov

500 participants, serum, BAL, PBMC, skin samples, USA	https://clinicaltrials.gov/ct2/show/NCT00001230?recrs=ae&cond=lymphatic+filariasis&draw=2&rank=39	Thomas Nutman, NIAID tnutman@mail.nih.gov
360 participants, serum samples, Mali	https://clinicaltrials.gov/ct2/show/NCT00341666?recrs=ae&cond=lymphatic+filariasis&draw=2&rank=42	NIAID and University of Bamako

SOIL-TRANSMITTED HELMINTHIASIS, TSP Report

Introduction

Soil-transmitted Helminthiasis (STH) is caused by soil-transmitted helminths. Soil-transmitted helminths refer to the intestinal worms infecting humans that are transmitted through contaminated soil: *Trichuris trichiura, Ancylostoma duodenale, Necator americanus*, and *Ascaris lumbricoides*. Other species, such as *Ancylostoma ceylanicum* or *Oesophagostomum bifurcum*, can occasionally be found in specific geographical areas.

Ancylostoma duodenale and *Necator americanus* are referred to as hookworms. Larvae are carried in blood vessels and eggs are shed in the stool. *Trichuris trichiura* is referred to as a whipworm. The life cycle of *Trichuris trichiura* includes eggs, larvae and worms in the colon and cecum. The life cycle of *Ascaris lumbricoides* includes larvae in the circulation, the lungs, bronchial tree and throat, and worms and eggs in the stool.

WHO has set 2030 target goals related to STH, which include establishment of an efficient STH control program in adolescent, pregnant and lactating women. STH can be treated with albendazole, mebendazole or ivermectin.

A *Soil-transmitted Helminthiasis* virtual biobank is expected to support findability and availability of biospecimen panels for (i) EQA programs (or equivalent inter-laboratory exercises), reference and quality control material production (ii) method validation, including evaluation and/or PQA, and (iii) R&D other than validation of the analytical and clinical performance characteristics of a diagnostic test. Such R&D includes the identification of novel diagnostic biomarkers and/or their preliminary evaluation.

Scope

This document is intended as a description of the needs of biological materials in terms of *Soil-transmitted Helminthiasis* panels.

The scope of this document includes the needs of biospecimens for development, then validation of molecular biology (nucleic acid-based) assays and assays based on detection or measurement of analytes, other than nucleic acids, found in biological fluids: whole blood, serum/plasma, urine, stool. Assays intended to be used both in a context of monitoring and evaluation are in the scope. *Trichuris trichiura, Ancylostoma duodenale, Necator americanus*, and *Ascaris lumbricoides* are the primary focus of this document.

The following are out of the scope of this document: morphological assays (microscopy based), endoscopic/proctoscopic examinations.

NOTE. Although samples (stool, urine and blood/serum) from experimentally infected animals (pigs, dogs, mouse) are interesting, allowing us to get insights into the dynamics and

performance of the diagnostic biomarkers over the life cycle of the parasites, these are not in the scope of the report.

Sources

The content of this report is based on information found in the **References,** and consultation with the **DTAG**-NTD on STH. Periodic revision can be made as information on ongoing and scheduled diagnostic development projects, funded by different donors becomes available.

An interview with Dr Lieven Stuyver (J&J) was also conducted.

Biological diagnosis of Soil-transmitted Helminthiasis

Stool samples can be examined microscopically for parasite eggs. The Kato–Katz thick smear technique is widely used for STH epidemiological field surveys and is recommended by the WHO for surveillance and monitoring of STH control programs. Though the specificity is high, the sensitivity of Kato–Katz thick smear in single stool sample examination is limited by day-to-day variation in egg excretion leading to measurement error in estimating the presence of infection. Alternative direct parasitological assays include (i) centrifugation-based stool concentration techniques, such as Formol Ether Concentration (FEC) and Parasep, and (ii) flotation-based stool concentration techniques, such as the FLOTAC, mini-FLOTAC, FEKPAK^{G2} and McMaster techniques, all combined with microscopy. Though a true 'gold standard' test with 100% accuracy does not exist, Kato–Katz thick smears is so far commonly and widely used as the basic and 'default' technique for helminth epidemiology.

For *Ancylostoma duodenale* and *Necator americanus*, the culture-based Koga agar plate culture (APC) method is used, while the microscopy-based Baermann-Moraes spontaneous sedimentation technique (SST) or a formol ether concentration step (FECT) constitute alternatives.

Preanalytical challenges that can reduce sensitivity in stool microscopic diagnosis of helminthiases are related to delays in preparation, time and temperature of incubation (Harada Mori technique) and to the stool clarification step.

Although microscopy-based assays are not in the scope of this document, traditional microscopy is still considered as the reference method and therefore biospecimen annotation with microscopy data is important. Technological perspectives for the future include digital microscopy, potentially in combination with microfluidics.

Important biospecimen annotations

- Necessary annotation: egg counting results based on Kato-Katz thick smear.
- Useful annotations: measurements of hemoglobin, ferritin, eosinophilia in blood; detection of occult blood in stool

Nucleic acid based (molecular biology) assays

PCR assays targeting helminth-specific sequences, in DNA extracted from stool, but potentially also from urine, can be used for mass screening. DNA degradation is avoided when using stabilized stool, however formalin must not be used when DNA analyses are intended. Instead, commercially available nucleic acid stabilizing solutions can be used. The following stool preservative solutions, with corresponding fitness-for-purpose specifications, can be used, in a 1:3 stool:preservative ratio, or as recommended by the manufacturer:

Sodium acetate-acetic acid-formalin	Morphology, microscopy, immunoassays
Schaudinn's Fixative<br>polyvinyl-alcohol	Morphology, microscopy, trichrome staining
Non-formalin-containing, commercially available stabilizers for nucleic acids	Nucleic acid analysis

Other critical preanalytical variables include the DNA extraction method/kit, the composition of lysis buffer and the stool homogenization method used (tissue homogenizer, bead beating, heating, type of beads), as the parasitic ova are not easy to lyse. The most difficult to lyse eggs are those of *Trichuris trichiura*. If using urine, critical preanalytical variables are the processing times and temperature, the centrifugation conditions, and the cryopreservation temperature.

qPCR may detect one single egg in 200mg of stool, under optimal pre-analytical and analytical conditions. The amplification gene target is usually the cytochrome oxidase I gene, the β tubulin isotype 1 gene (*A. lumbricoides, T. trichiura, N. americanus*) or an ITS-1 (*A. lumbricoides*) or ITS-2 (*T. trichiura, N. americanus*) target (in tests that have been developed to date).

PCR based assays in serum/plasma can target specific helminth ccfRNA molecules. In this context, critical preanalytical factors include the presence of stabilizer in the blood collection tube, the blood pre-centrifugation time and temperature (time to centrifugation), the serum/plasma freezing temperature, the freeze-thaw cycles and the nucleic acid extraction kit or method used.

As a sidenote, PCR in feces, is generally specific and sensitive enough, to be used not only for diagnosis, but also for monitoring response to treatment.

Non nucleic acid based (e.g. serological) assays

Antibody-based assays

Serological assays for anti-helminthic antibodies – in serum/plasma/dried blood spot samples – are used for screening programs and individual diagnosis of chronic infections (together with direct parasitological assays in stool). To date, such assays have been developed for *Ascaris* infections. Serological assays may not be sufficiently sensitive for detection of acute infection, and specific antibodies do not appear before the sixth week of infection. A decrease in antibody titer over sequential specimens may also be an indicator of successful therapy. Most commercially available ELISA, IFAT, immunoblot and LIPS assays are based on helminthic larvae crude lysates.

The IgG subclass may have relevance in detection of acute (IgG1) or chronic (IgG4) infections, while other subclasses (IgM, IgA, IgE) might be indicative of different stages of infection. Specific IgA may be detectable, not only in blood, but also in saliva.

Non-antibody based assays

Assays for helminthic copro-antigen detection could be used as POC. For the moment, an ELISA for detection of the *Ascaris lumbricoides* BA-1 antigen has been developed, and another copro-antigen-based assay for detection of *Ascaris, Trichuris* and *Ancylostoma* has been developed, but only for veterinary use.

Metabolite based biomarkers in plasma or urine could be of interest in the context of *Ascaris lumbricoides* (Dr Lieven Stuyver).

Potential serologic cross reactivities (important to inform the needs for validation of specificity, as relevant, depending on the analyte):

- When measuring antibodies, cross reactivity may exist between
    - different soil-transmitted helminths, including *Strongyloides stercoralis*
    - soil-transmitted helminths and filarial parasites or schistosomes
    - soil-transmitted helminths and *Echinococcus* or *Toxocara*

Table 1 gives the different contexts of use of diagnostic tests and corresponding sample types and method types.

Table 1

Context of use	Sample type and method
Monitoring and evaluation *Trichuris trichiura, Ancylostoma duodenale, Necator americanus*, and *Ascaris lumbricoides*	Stool (NAAT, antigen or other biomarker),<br>Urine (NAAT, metabolites),<br>DBS (immunoenzymatic method for specific antibody subclasse(s) or other biomarkers, such as ccfRNA or metabolites)<br>Serum/plasma (immunoenzymatic method for specific antibodies subclasse(s) or other biomarkers, such as ccfRNA or metabolites)

Need for samples corresponding to different helminth strains:

Both *Necator americanus* and *Ancylostoma duodenale* are found in Africa, Asia, Australia and the Americas. Only *N. americanus* is found in south India and predominates in the Americas, while only *A. duodenale* is found in the Middle East, North and Eastern Africa, and northern India.

Trichuris trichiura and *Ascaris lumbricoides* are found worldwide, but are most common in the tropics, subtropics, and in warm temperate regions.

No impact on diagnostic performance has been reported based on geographically different strains of the same helminthic species (for currently available tests).

Reference methods:

- For *Trichuris trichiura, Ancylostoma duodenale, Necator americanus*, and *Ascaris lumbricoides*: Kato-Katz thick smear (or equivalent direct parasitological) fecal examination-based egg counting results.

Reference materials

No WHO international standard exists for STH.

FDA reference panels

No FDA reference panel for STH could be found.

Validated (FDA approved) methods

No validated FDA approved methods for STH could be found.

Other methods that have been made/are available

- *Ascaris lumbricoides/Ascaris suum* Techne qPCR test (Techne)
- AllPlex GI helminth(I) assay (Seegene) (*Ancylostoma* sp, *Ascaris* sp, *Necator americanus*, *Strongyloides* sp, *Trichuris trichiuria*)
- LightMix modular *Ascaris lumbricoides* PCR (TIB MolBiol)
- LightMix modular *Ancylostoma duodenale* PCR (TIB MolBiol)
- *Ascaris lumbricoides* IgG ELISA (MyBioSource)
- Human anti-*Ascaris lumbricoides* IgG ELISA kit (Abcam)
- *Ascaris lumbricoides* IgG assay kit (Demeditec)
- *Ascaris lumbricoides* IgG ELISA (Immuno-Biological Laboratories – America)
- *Ascaris lumbricoides* IgG ELISA kit (Creative Diagnostics)

No information on the composition of the panels that have been used by the companies for method evaluation could be found for any of these tests.

EQA programs

No commercially available EQA program for any parasitic disease and methods other than microscopy, except for toxoplasmosis, could be found on the CDC site.

On the EPTIS website, a general parasitology scheme for helminths is listed as being provided by RCPAQAP (Australia), and a digital scheme for helminth (*Ascaris, Trichuris, Ancylostoma, Taenia*) eggs in water, biosolids and sand is provided by Hydrolab Microbiologia (Spain).

A Helminth External Molecular Quality Assessment Scheme (HEMQAS) has been provided by the Dutch Foundation for Quality Assessment in Medical Laboratories (Cools 2020).

The results from a recently organized academic EQA scheme (organized by the National Institute of Parasitic Diseases (NIPD) of Chinese Center for Disease Control and Prevention (CDC), see Lu et al., with 10 Kato Katz thick smear items and 2 serum items) have recently been published. This gives an idea of current EQA panels.

Panel needs

R&D / method validation needs

No data from any FDA submission that could be used to inform us on number of reference positive samples used for sensitivity assessment, number of reference negative samples for specificity assessment, number and type of samples for cross reactivity assessment, could be found.

Table 2a shows the needs in terms of panels for initial development /feasibility studies, for both nucleic acid-based and non-nucleic acid-based assays.

Table 2a

Sample type	Quantity per donor (for 1 development panel)	Number of biospecimen donors
Serum/ plasma	0,5ml	30 *Trichuris trichiura* positive cases<br>30 *Ancylostoma duodenale* positive cases<br>30 *Necator americanus* positive cases<br>30 *Ascaris lumbricoides* positive cases<br>30 *Trichuris trichiura, Ancylostoma duodenale, Necator americanus, Ascaris lumbricoides* negative cases
Whole blood or DBS	0,4ml WB or 2 DBS	30 *Trichuris trichiura* positive cases<br>30 *Ancylostoma duodenale* positive cases<br>30 *Necator americanus* positive cases<br>30 *Ascaris lumbricoides* positive cases<br>30 *Trichuris trichiura, Ancylostoma duodenale, Necator americanus, Ascaris lumbricoides* negative cases
Urine	2ml	30 *Trichuris trichiura* positive cases<br>30 *Ancylostoma duodenale* positive cases<br>30 *Necator americanus* positive cases<br>30 *Ascaris lumbricoides* positive cases<br>30 *Trichuris trichiura, Ancylostoma duodenale, Necator americanus, Ascaris lumbricoides* negative cases
Stool	4g	30 *Trichuris trichiura* positive cases

		30 *Ancylostoma duodenale* positive cases<br>30 *Necator americanus* positive cases<br>30 *Ascaris lumbricoides* positive cases<br>30 *Trichuris trichiura, Ancylostoma duodenale, Necator americanus, Ascaris lumbricoides* negative cases

Table 2b shows the needs in terms of complete validation panels for each indication and for either nucleic acid-based or non-nucleic acid-based assays. Numbers of donors are defined based on the published WHO TPP.

Table 2b

Sample type	Quantity per donor (for 1 validation panel)	Number of biospecimen donors
Serum/ plasma	0,5ml	90(1) *Trichuris trichiura* positive cases<br>90(1) *Ancylostoma duodenale* positive cases<br>90(1) *Necator americanus* positive cases<br>90(1) *Ascaris lumbricoides* positive cases<br>90(1) *Strongyloides stercoralis* positive cases<br>90(1) *Enterobius vermicularis* positive cases<br>90(1) negative cases for all above
Whole blood or DBS	0,2ml WB or 1 DBS	90(1) *Trichuris trichiura* positive cases<br>90(1) *Ancylostoma duodenale* positive cases<br>90(1) *Necator americanus* positive cases<br>90(1) *Ascaris lumbricoides* positive cases<br>90(1) *Strongyloides stercoralis* positive cases<br>90(1) *Enterobius vermicularis* positive cases<br>90(1) negative cases for all above
Urine	1ml	90(1) *Trichuris trichiura* positive cases<br>90(1) *Ancylostoma duodenale* positive cases<br>90(1) *Necator americanus* positive cases

		90(1) *Ascaris lumbricoides* positive cases<br>90(1) *Strongyloides stercoralis* positive cases<br>90(1) *Enterobius vermicularis* positive cases<br>90(1) negative cases for all above
Stool	200mg	90(1) *Trichuris trichiura* positive cases<br>90(1) *Ancylostoma duodenale* positive cases<br>90(1) *Necator americanus* positive cases<br>90(1) *Ascaris lumbricoides* positive cases<br>90(1) *Strongyloides stercoralis* positive cases<br>90(1) *Enterobius vermicularis* positive cases<br>90(1) negative cases for all above

[1] applicable to a monitoring/evaluation context of use with high prevalence (assumed prevalence 30%, expected sensitivity 60%, expected specificity 99%, desired precision 10%). Collection should be from a region under endemic conditions.

Positivity status is an attribute of the donor. Positivity is defined by positive direct parasitological morphological assays with monomorphic results by a reference method, applied to at least one relevant biospecimen type from the donor.

Sample sizes have been estimated according to the NM Fenn Buderer statistical approach, for the desired diagnostic sensitivity and specificity levels, using PASS 2021 software at an actual significance level between 0.05 and 0.15 and with 80% power for a diagnostic evaluation to detect a reduction in sensitivity or specificity of 10%.

Complete biospecimen sets, including all biospecimen types, from the same donor are preferred. It is critical that for a validation panel, all biospecimens come from the same collection, to avoid preanalytical bias. Preanalytical bias is unavoidable if using biospecimens from different collections with different or undocumented preanalytical specifications. It would be interesting if results from multiple end users / developers would be collected in a common database.

Possible retrospective sources of such specimens can be found in published literature and in completed or ongoing clinical trials (Annex 1). The most suitable sources, especially relative to STH positivity status based on active worms, would be **longitudinal** collections, including both pre-treatment and post-treatment samples.

Although the first priority is the needs for development, then for validation of new methods, in order for a new method to be deployed in the field, an external quality assurance (EQA) program is needed, as well as quality control (QC) materials to be included in the kits.

EQA needs

The needs for EQA panels for nucleic acid based and non-nucleic acid-based assays are listed below. Table 3 shows the minimum necessary quantities per panel for **one EQA scheme and round** for **40 participating laboratories**, with each laboratory receiving 0,1ml serum/plasma, 200mg stabilized stool, and/or 1 DBS. The quantities correspond to the quantities to be distributed to the participating laboratories, supplemented by the quantities necessary to perform homogeneity and stability testing, and value assignment by the EQA provider.

The current best source of historical samples would be STH screening centers in endemic countries.

Table 3

	**Sample type**	**Quantity per item (for one EQA round)**	**Number of items**
Non nucleic acid based	Serum (or plasma), antibody-based	6ml*	1 item *T. trichiura* positive<br>1 item *A. lumbricoides* positive<br>2 items negative for the above
Nucleic acid based	Stool	10g*	1 item *T. trichiura* positive<br>1 item *A. lumbricoides* positive<br>1 item *Necator americanus* positive<br>1 item *Ancylostoma duodenale* positive<br>2 items negative for all above

** can be pooled material*

Note. It is preferred that stool items for STH nucleic acid-based methods be native fecal specimens to allow EQA assessment of the performance of **both** the DNA extraction phase and the PCR amplification phase.

Commercialized kit, QC material needs

For one serology-based kit, depending on the kit specifications in terms of target and matrix, one of the following may apply

- At least 500ml of pooled *A. lumbricoides* serum/plasma

For PCR based kits, QC materials would be isolated parasitic DNA samples.

For antigen detection kits, an antigen solution can be used (no need for biological QC materials).

References consulted for the Soil-transmitted Helminthiasis TSP

EM O'Connell, TB Nutman. Molecular diagnostics for soil-transmitted helminthes. Am J Trop Hyg 2016;95:508-513.

S Khurana et al. Diagnostic techniques for soil-transmitted helminths. Recent advances. Res Rep Trop Med 2021;12:181-196.

SP van Mens et al. Comparison of real-time PCR and Kato smear microscopy for the detection of hookworm infections in three consecutive faecal samples from schoolchildren in Ghana. Trans R Soc Trop Med Hyg 2013;107:269–271.

EI Odongo-Aginya et al. Substitution of malachite green with nigrosin–eosin yellow stain in the Kato-Katz method: microscopical appearance of the helminth eggs. Afr J Health Sci 2007;7:33–36.

https://www.who.int/news-room/fact-sheets/detail/soil-transmitted-helminth-infections

M Ayana et al. Comparison of four DNA extraction and three preservation protocols for the molecular detection and quantification of soil-transmitted helminths in stool PLOS Negl Trop Dis 2019;13:e0007778.

P Cools et al. Diagnostic performance of a single and duplicate Kato-Katz, Mini-FLOTAC, FECPAK^{G2} and qPCR for the detection and quantification of soil-transmitted helminths in three endemic countries. PLOS Negl Trop Dis 2019;13:e0007446.

H Wilke & LJ Robertson. Preservation of Giardia cysts in stool samples for subsequent PCR analysis. J Microbiol Methods 2009;78:292–296.

S Kuk et al. Stool sample storage conditions for the preservation of Giardia intestinalis DNA. Mem Inst Oswaldo Cruz 2012;107:965–968.

SA Repetto et al. An improved DNA isolation technique for PCR detection of Strongyloides stercoralis in stool samples. Acta Trop 2013;126:110–114.

D Leles et al. Molecular diagnosis of ascariasis from human feces and description of a new Ascaris sp. genotype in Brazil. Vet Parasitol 2009;163:167–170.

AV Easton et al. Multi-parallel qPCR provides increased sensitivity and diagnostic breadth for gastrointestinal parasites of humans: field-based inferences on the impact of mass deworming. Parasit Vectors 2016;9:38.

JJ Verweij et al. Simultaneous detection and quantification of Ancylostoma duodenale, Necator americanus, and Oesophagostomum bifurcum in fecal samples using multiplex real-time PCR. Am J Trop Med Hyg 2007;77:685–690.

RJ Traub et al. PCR-based coprodiagnostic tools reveal dogs as reservoirs of zoonotic ancylostomiasis caused by Ancylostoma ceylanicum in temple communities in Bangkok. Vet Parasitol 2008;155:67–73.

JX Wang et al. Application of a real-time PCR method for detecting and monitoring hookworm Necator americanus infections in southern China. Asian Pac J Trop Biomed 2012;2:925–929.

R Tello et al. Highly effective and inexpensive parasitological technique for diagnosis of intestinal parasites in developing countries: spontaneous sedimentation technique in tube. Int J Infect Dis 2012;16:e414–416.

MH Anna et al. Parasite derived microRNA in host serum as novel biomarkers of helminth infection. PLoS Negl Trop Dis 2014;8:e2701.

N Lodh et al. Diagnosis of Strongyloides stercoralis: detection of parasite-derived DNA in urine. Acta Trop 2016;163:9-13.

MLS Mationg et al. Status of soil-transmitted helminth infections in schoolchildren in Laguna Province, the Philippines: Determined by parasitological and molecular diagnostic techniques.

M Papaiakovou et al. How qPCR complements the WHO roadmap (2021-2030) for soil-transmitted helminths. Trends in Parasitology 2021;37:698-708.

P Cools et al. Quantitative PCR in soil-transmitted helminth epidemiology and control programs: toward a universal standard. PLOS Negl Trop Dis 2021;15:e0009134.

LR Bosqui et al. Detection of parasite-specific IgG and IgA in paired serum and saliva samples for diagnosis of human strongyloidiasis in northern Parana state, Brazil. Acta Trop 2015;150:190-195.

B Levecke et al. Efficacy of mebendazole in school children in six countries where soil-transmitted helminths are endemic. PLoS Negl Trop Dis 2014;8:e3204.

T Kolle et al. Comparison of commercial and in-house real-time PCR platforms for 15 parasites and microsporidia in human stool samples without a gold standard. Acta Tropica 2020;

https://www.techion.com/FECPAKG2

M Ayana et al. Modification and optimization of the FECPAKG2 and the Lato-Katz method for analyzing soil-transmitted helminth eggs in human stool. PLoS Negl trop Dis 2018;12:e0006655.207:105516.

O Lagatie et al. Methyl-pentanoyl-varnitine (2-MPC): a urine biomarker for patent Ascaris lumbricoides infection. Sci Rep 2020;10:15780.

B Jimenez et al. Identification and quantification of pethogenic helminth eggs using a digital image system. Exp Parasitol 2016;166:164-172.

https://www.cdc.gov/parasites/sth/index.html

https://www.cdc.gov/parasites/strongyloides/

https://www.who.int/publications/i/item/9789240031227

https://www.nibsc.org/products/brm_product_catalogue/who_standards.aspx

https://www.accessdata.fda.gov/scripts/cdrh/devicesatfda/index.cfm

https://www.biocompare.com/Search-ELISA-Kits/?search=ELISA+Ascaris

https://www.eptis.org/

FD Halstead et al. Universal extraction method for gastrointestinal pathogens. J Med Microbiol 2013;62:1535-1539.

P Cools et al. First international external quality assessment scheme of nucleic acid amplification tests for the detection of Schistosoma and soil-transmitted helminths, including Strongyloides: A pilot study. PLOS Negl Trop Dis 2020;14:e0008231.

Y Lu et al. Urgent needs in fostering neglected tropical diseases (NTDs) laboratory capacity in WHO Western Pacific Region: results from the external quality assessment on NTDs diagnosis in 2012-2015. Infect Dis Poverty 2017;6:106.

NM Fenn Buderer. Statistical methodology : I. Incorporating the prevalence of disease into the sample size calculation for sensitivity and specificity. Acad Emerg Med 1996;3:895-900.

Acronyms

ccfRNA	Circulating cell free RNA
DBS	Dry blood spot
DTAG-NTD	Diagnostic Technical Advisory Group for Neglected Tropical Diseases
ELISA	Enzyme linked immunosorbent assay
EQA	External Quality Assurance
FECT	Formol ether concentration technique
HEMQAS	Helminth External Molecular Quality Assessment Scheme
IFAT	Indirect antibody fluorescent test
ITS	Internal transcribed spacer
LAMP	Loop mediated isothermal amplification
LIPS	Luciferase immunoprecipitation system
LOD	Limit of detection
MTA	Material transfer agreement
NAAT	Nucleic acid amplification test
POC	Point of care
PQA	WHO prequalification assessment
RDT	Rapid diagnostic test
SST	Spontaneous sedimentation technique

FIND Diagnosis for all

Annex 1

Specifications of potential sources of biospecimens from published studies and from completed or ongoing clinical trials. Included are articles published after 2015 and clinical trials with at least 100 participants and completed after 2010.

Type of collection	Reference	Contact
325 children from Philippines<br><br>DNA from stool	doi: 10.1371/journal.pntd.0006022	Darren Gray, Research School of Population Health, The Australian National University, Canberra, Australia darren.gray@anu.edu.au
650 children from Myanmar<br><br>Stool and stool DNA samples	doi: 10.1186/s13071-020-04197-w	Julia Dunn, Imperial College, London, ulia.dunn@imperial.ac.uk1
2800 children from Bangladesh<br><br>Stool and stool DNA samples	doi: 10.1371/journal.pntd.0008087	Jade Benjamin Chung, University of California, Berkeley, jadebc@berkeley.edu
500 donors from Tanzania<br><br>Stool and stool DNA samples	doi: 10.1186/s13071-020-04401-x	Jennifer Keiser, Swiss Tropical and Public Health Institute, jennifer.keiser@swisstph.ch
320 donors from Tanzania<br><br>Stool and stool DNA samples	doi: 10.1186/s12866-020-01963-9	Jennifer Keiser, Swiss Tropical and Public Health Institute, jennifer.keiser@swisstph.ch
300 donors from Ethiopia<br><br>Stool and serum samples	doi: 10.1186/s13071-019-3308-z	Ole Lagatie, Jehnssen Diagnostics Belgium, olagatie@its.jnj.com
Xxx donors from Indonesia	doi: 10.1186/s12879-015-0873-4	Taniawati Supali, department of Parasitology, Faculty of

FIND Diagnosis for all

Stool, stool DNA, serum, plasma, PAXgene blood samples		Medicine, Universitas Indonesia,<br>Jakarta, Indonesia,<br>taniawati@yahoo.com
680 participants, two follow up stool samples, Ethiopia	https://clinicaltrials.gov/ct2/show/NCT02420574?recrs=ae&cond=soil+transmitted+helminth&draw=2&rank=21	Jozef Vercruysse, University Ghent
620 participants, serum samples, Tanzania	https://clinicaltrials.gov/ct2/show/NCT00347113?recrs=ae&cond=soil+transmitted+helminth&draw=2&rank=17	Safari M Kinung'hi, ational Insitute for Medical Research, Mwanza, Tanzania
200 participants, stool samples, Sri Lanka	https://clinicaltrials.gov/ct2/show/NCT01350271?recrs=ae&cond=soil+transmitted+helminth&draw=2&rank=12	Nilanthi R de Silva, Faculty of Medicine, University of Kelaniya, Sri Lanka
250 participants, stool samples, Argentina, Australia, Brazil, Cambodia, Vietnam, Cameroon, Ethiopia, Tanzania	https://clinicaltrials.gov/ct2/show/NCT01379326?recrs=ae&cond=soil+transmitted+helminth&draw=2&rank=11	Bruno Levecke, University Ghent,<br>bruno.levecke@ugent.be
20000 participants, stool and stool DNA samples, Kenya	https://clinicaltrials.gov/ct2/show/NCT02397772?recrs=ae&cond=soil+transmitted+helminth&draw=2&rank=9	Rachel L Pullan, London School of Hygiene and Tropical Medicine
250 participants, stool and stool DNA samples, Gabon	https://clinicaltrials.gov/ct2/show/NCT04326868?recrs=ae&cond=soil+transmitted+helminth&draw=2&rank=7	Ayôla Akim ADEGNIKA, Centre de Recherche Médicale de Lambaréné

1000 participants, Ethiopia, Tanzania, Lao PDR, Brazil	https://clinicaltrials.gov/ct2/show/NCT03465488?recrs=ae&cond=soil+transmitted+helminth&draw=2&rank=6	Bruno Levecke, University Ghent, bruno.levecke@ugent.be
4700 participants, stool, stool DNA, DBS samples, Fiji	https://clinicaltrials.gov/ct2/show/NCT03177993?recrs=ae&cond=soil+transmitted+helminth&draw=2&rank=3	Andrew Steer, Murdoch Children Research Institute
8000 participants, stool and stool DNA samples, Cambodia, Bangladesh, Lao PDR, Ghana, Senegal, Rwanda	https://clinicaltrials.gov/ct2/show/NCT04177654?recrs=ae&cond=soil+transmitted+helminth&draw=2&rank=1	Bruno Levecke, University Ghent, bruno.levecke@ugent.be
Paired serum/stool samples	https://www.starworms.org/samples	Bruno Levecke, University Ghent, bruno.levecke@ugent.be

TRACHOMA, TSP Report

Introduction

Trachoma is caused by the trachoma biovar of the obligate intracellular bacterium *Chlamydia trachomatis* that infects mucosal epithelial cells. Trachoma represents around 15% of all the cases of blindness, associated with corneal opacification. The fly *Musca sorbens* is believed to be the vector of the disease. The bacterium has two metabolically and phenotypically distinct forms, the infective extracellular elementary bodies and the intracellular reticulate bodies. *C. trachomatis* wall mainly consists of lipopolysaccharide (LPS) and the Major Outer Membrane Protein (MOMP), both containing immunogenic epitopes.

Although diagnostics is not named as a pillar of the WHO Global Elimination of Trachoma (GET) SAFE strategy, reliable diagnostics are necessary for reliable estimation of prevalence. Estimation of prevalence is what guides implementation of programs of antibiotherapy and environmental improvements, both parts of the SAFE strategy. The SAFE strategy includes **S**urgery for trichiasis, **A**ntibiotic (azithromycin) distribution, **F**acial cleanliness, **E**nvironmental improvements. WHO recommends MDA in communities where follicular trachoma is >10% in children.

A *Chlamydia trachomatis* (biovar trachoma) virtual biobank is expected to support findability and availability of biospecimen panels for (i) EQA programs (or equivalent inter-laboratory exercises), reference and quality control material production (ii) method validation, including evaluation and/or PQA, and (iii) R&D other than validation of the analytical and clinical performance characteristics of a diagnostic test. Such R&D includes the identification of novel diagnostic biomarkers and/or their preliminary evaluation.

Scope

This document is intended as a description of the needs of biological materials in terms of *Chlamydia trachomatis* (biovar trachoma) panels.

The scope of this document includes the needs of biospecimens for development, then validation of assays based on detection or measurement of analytes, other than nucleic acids, found in biological fluids (serum, plasma, dried blood spot, dried serum spot). Assays intended to be used, in a context of mapping, stopping massive drug administration (MDA), post-elimination surveillance, or longitudinal immunological studies, are in the scope.

The following are **out of the scope** of this document: **nucleic acid-based assays**, microscopic detection of chlamydial inclusions or antigens, based on conjunctival scrapings or swabs, as well as cell culture of infectious *Chlamydiae*. *In vivo* confocal microscopy is also out of scope. Nucleic acid based assays are out of the scope because high performance diagnostic tools are already on the market.

Sources

The content of this report is based on information found in the **References,** and consultation with the **DTAG**-NTD on trachoma. Periodic revision can be made as information on ongoing and scheduled diagnostic development projects, funded by different donors becomes available.

Clinical and biological diagnosis of trachoma

The gold standard for the diagnosis of active trachoma is clinical and based on presence of follicles or intense trachomatous inflammation. Clinical signs include papillary reaction and thickening of the tarsal conjunctiva, with follicles, obscuration of the deep tarsal vessels, occasional eyelid edema and corneal inflammation. The latter is assessed based on superficial punctate keratitis (following fluorescein instillation) and superficial infiltrates or pannus. Trichiasis and/or entropion are also distinctive clinical signs. Diagnosis of repeated or past trachoma is also clinical and based on presence of scarring of the subepithelial conjunctiva (Herbert's pits).

Apart from the clinical symptom-based diagnosis, direct biological diagnosis of trachoma is based on microscopical examination of (i) Giemsa/Iodine stained conjunctival scrapings, for detection of inclusions, or (ii) fluorescein antibody stained conjunctival swabs, for detection of a species-specific MOMP epitope. The gold standard for biological diagnosis has been considered to be cell culture, however this has very stringent requirements relative to the cold chain maintenance and complex operating procedure. Nowadays, NAAT is considered as gold standard, although NAAT based on DNA targets does not assess the presence of viable microorganisms.

There is a poor correlation, especially in low prevalence settings, between laboratory diagnosis of infection and clinical symptoms, due to an initial long latent phase (with presence of bacteria but no clinical symptoms) and a long recovery phase (with clinical symptoms but no detectable bacteria).

Biospecimen annotations

- Potentially interesting annotation for *Chlamydia trachomatis* (trachoma biovar): standardized clinical examination data, based on the WHO simplified system (Thylefors 1987), including presence or absence of
  - TF, trachomatous inflammation, follicular, presence of 5 or more follicles of at least 0,5mm diameter in the central part of the upper tarsal conjunctiva
  - TI, trachomatous inflammation, intense, pronounced inflammatory thickening of the upper tarsal conjunctiva obscuring more than half the normal deep tarsal vessels
  -

- TS, trachomatous conjunctival scarring, presence of visible scars in the tarsal conjunctiva
    - TT, trachomatous trichiasis, at least one eyelash rubs on the eyeball or evidence of recent removal of in-turned eyelashes
    - CO, corneal opacity visible over the pupil, so dense that at least part of the pupil margin is blurred when viewed through the opacity
- Useful annotation for *Chlamydia trachomatis*: epithelial cell count in the context of direct IF test.
- Important annotation for *Chlamydia trachomatis*: information on history of urogenital *Chlamydia trachomatis* infection.

NOTE. In this report, we take the hypothesis that there is not significant immunological cross reactivity between *C. trachomatis* and *C. pneumoniae*.

Non nucleic acid based (serological) assays

The microimmunofluorescence (MIF) technique is a historical, indirect fluorescent antibody assay, of poor specificity, sensitivity and reproducibility, using whole elementary bodies. Serological diagnosis of trachoma, using serum/plasma, tears or DBS, is based on IgM, IgA or IgG antibodies against LPS, MOMP, Pgp3 or other antigens. Serological assays do not easily discriminate between acute and chronic infections. Particularly, in case of reinfection with the same serovar, no IgM is produced. However, the titers of sequential samples may be useful in determining seroconversion and hence active infection.

Currently developed (non-commercial) LFA tests work with serum, but not all work with DBS samples.

For antibody-based assays, critical preanalytical factors include the long-term storage conditions.

Potential cross reactivities (important to inform the needs for validation of specificity, as relevant, depending on the analyte):

When performing serology for anti-*C. trachomatis* antibodies, specificity can be established against *C. pneumoniae*. Indeed, most chlamydial proteins or peptides present cross reactivities between chlamydial species. This is a problem because of the high seroprevalence of up to 80% of *C. pneumoniae*. In case of LPS-based antigen, specificity can be established against the frequent conjunctivitis etiological bacteria ***Staphylococcus aureus****, Streptococcus pneumoniae*, *Haemophilus* species, *Moraxella catarrhalis,* ***Moraxella lacunata****,* and the less frequent *Neisseria gonorrhoeae* (gonococcal conjunctivitis), *Pseudomonas aeruginosa, Streptococcus viridans*, and *Proteus mirabilis*.

Table 1 gives the different contexts of use of diagnostic tests and corresponding sample types and method types.

Table 1

Context of use	Sample type and method
Mapping	Serum/plasma or DBS (IgG/IgM ELISA or other biomarkers)
Stopping MDA	Serum/plasma or DBS (IgG ELISA or other biomarkers)
Post elimination surveillance	Serum/plasma or DBS (IgG/IgM ELISA or other biomarkers)
Longitudinal	DBS (IgG ELISA)

Need for samples corresponding to different *Chlamydia trachomatis* strains:

The main relevant geographical area for *Chlamydia trachomatis* biospecimen collection is Africa, the Middle East, Central and South America, Asia, Australia and the Pacific islands. India is a major endemic site.

There are 15 serovars of the trachoma biovar of *C. trachomatis*. These are the A, B, Ba, C (most usual ones in trachoma), D, Da, E, F, G, Ga, H, I, Ia, J, and K. The MOMP contains serovar-specific epitopes.

Therefore, collection of samples of different serovars is important if the analytical target is serovar specific. Collection of samples should be representative of trachoma endemic or previously endemic regions.

In the context of mapping, it is important to have access to sample sets from multiple collections in baseline/pre-intervention settings. In the context of stopping MDA, it is important to have access to sample sets from multiple collections of impact surveys. In the context of post-elimination surveillance, it is important to have access to samples sets from multiple collections in post-elimination settings.

Reference methods:

- Clinical diagnosis, accompanied by conjunctival scraping or swab microscopy and/or cell culture

Reference materials

No WHO international standard exists for trachoma.

Quality Control materials are made available by some EQA providers. For example, the Reference Institute for Bioanalytics (Germany) provides *C. trachomatis* DNA.

FDA reference panels

No FDA reference panel for trachoma could be found.

Validated (FDA approved) methods

All FDA approved methods are NAAT.

Other methods that have been described/made available

- Syva MicroTrak (Syva), based on IF
- Dako IDEIA Chlamydia (Dako), based on a monoclonal anti-LPS antibody
- MIF (Focus Diagnostics)
- LFA kits have been made available by the CDC in specific projects
- Luminex bead based assays have been implemented in some laboratories (home-made)

POC tests:

- Clearview Chlamydia MF (Clearview)
- Chlamydia Rapid Test (CRT) (DRW)
- Chlamydia test card (Ultimed Products)
- Biorapid Chlamydia Ag test (Quidel Corporation)
- ACON Chlamydia Rapid Test Device (ACON)
- CT Duo test combo (ACON)
- Cortez Onestep Chlamydia RapicardTM insta test (Cortez Diagnostics)
- BioStar Chlamydia TRF lit (Medisensor)
- Atlas Genetics io platform (Atlas Genetics)

EQA programs

Different EQA programs are available on the EPTIS website, of which the target property is related to *C. trachomatis*, though not specifically in the scope of trachoma. INSTAND (Germany) offers EQA schemes for anti-*C. trachomatis* antibody detection, for *C. trachomatis*

direct detection on slides and in microplates (ELISA). Other EQA providers include Controllab (Brazil), RCPAQAP (Australia).

Panel needs

R&D / method validation needs

Table 2a shows the needs in terms of panels for initial development /feasibility studies, for non-nucleic acid-based assays.

Table 2a

Sample type	Quantity per donor (for 1 development panel)	Number of biospecimen donors
Serum/ plasma	1ml	100 *C. trachomatis* positive cases<br>100 *C. trachomatis* negative cases
DBS	10ul (Trop Bio filter paper single extension)	100 *C. trachomatis* positive cases<br>100 *C. trachomatis* negative cases

Table 2b shows the needs in terms of complete validation panels with numbers of donors being defined based on a prevalence of 5%, corresponding to the WHO target of <5% prevalence of follicular trachoma. This, according to Pinsent 2018 corresponds to a seroprevalence threshold of 7%. Sample sizes have been estimated according to the NM Fenn Buderer statistical approach, for the desired diagnostic sensitivity and specificity levels, using PASS 2021 software at an actual significance level between 0.05 and 0.15 and with 80% power to detect a reduction in sensitivity or specificity of 10%. Serological cross reactivity with *C. pneumoniae* is considered.

Table 2b

**Sample type**	**Quantity per donor (for 1 validation panel)**	**Number of biospecimen donors**
Serum/ plasma	0,5ml	50[(1)(2)] *C. trachomatis* active acute trachoma / *C. pneumoniae* seronegative cases<br>50[(1)(2)] *C. trachomatis* active chronic trachoma / *C. pneumoniae* seronegative cases<br>50[(1)(2)] *C. trachomatis* past trachoma / *C. pneumoniae* seronegative cases<br>50[(1)(2)] *C. pneumoniae* seropositive / *C. trachomatis* seronegative cases<br>600[(1)(2)] *C. trachomatis/C. pneumoniae* seronegative cases<br>Mix of 100[(1)(2)] inflammatory conjunctivitis cases, due to *Staphylococcus aureus, Streptococcus pneumoniae*, *Haemophilus influenzae*, *Moraxella catarrhalis,* or *Moraxella lacunata*
Tears	1 cellulose or PVA mini sponge strip	50[(2)] *C. trachomatis* symptomatic active trachoma cases<br>900[(2)] *C. trachomatis* negative, non-infected cases<br>Mix of 100[(2)] inflammatory conjunctivitis cases, due to *Staphylococcus aureus, Streptococcus pneumoniae*, *Haemophilus influenzae*, *Moraxella catarrhalis,* or *Moraxella lacunata*
Conjunctival swab medium	0,5ml	50[(2)] *C. trachomatis* symptomatic active trachoma cases<br>900[(2)] *C. trachomatis* negative, non-infected cases<br>Mix of 100[(2)] inflammatory conjunctivitis cases, due to *Staphylococcus aureus, Streptococcus pneumoniae*, *Haemophilus influenzae*, *Moraxella catarrhalis,* or *Moraxella lacunata*

Whole blood or DBS	0,2ml WB or 1 DBS	50(1)(2) *C. trachomatis* active acute trachoma / *C. pneumoniae* seronegative cases<br>50(1)(2) *C. trachomatis* active chronic trachoma / *C. pneumoniae* seronegative cases<br>50(1)(2) *C. trachomatis* past trachoma / *C. pneumoniae* seronegative cases<br>50(1)(2) *C. pneumoniae* seropositive / *C. trachomatis* seronegative cases<br>600(1)(2) *C. trachomatis/C. pneumoniae* seronegative cases<br>Mix of 100(1)(2) inflammatory conjunctivitis cases, due to *Staphylococcus aureus, Streptococcus pneumoniae*, *Haemophilus influenzae*, *Moraxella catarrhalis,* or *Moraxella lacunata*

[1] applicable to a context of laboratory diagnosis of serological history of (past or chronic) trachoma (assumed prevalence inside an endemic area 7%, expected sensitivity 95%, expected specificity 95%, desired precision 10%)
[2] applicable to a context of laboratory diagnosis of active, acute or chronic, trachoma (assumed prevalence inside an endemic area 5%, expected sensitivity 95%, expected specificity 99%, desired precision 10%)

Positivity status is an attribute of the donor.

Positivity for active acute trachoma has been traditionally defined by clinical symptoms (follicles, intense conjunctival inflammation), and positive chlamydial antigen detection in conjuctival smear. Positivity for the presence of viable *Chlamydiae* can be assessed on the basis of positive cell culture or positive *C. trachomatis*-specific mRNA based test.

Positivity for active chronic trachoma can be defined by scarring, trichiasis, and positive NAAT.

Positivity for past trachoma can be defined by scarring, trichiasis, historical positive NAAT and recent negative NAAT.

Possible retrospective sources of specimens can be found in published literature and in completed or ongoing clinical trials (Annex 2). The most suitable sources for identification and validation of acute versus chronic disease-specific biomarkers would be **longitudinal** collections from trachoma patients.

Although the first priority is the needs for development, then for validation of new methods, in order for a new method to be deployed in the field, an external quality assurance (EQA) program is needed, as well as quality control (QC) materials to be included in the kits.

EQA needs

The needs for EQA panels for non-nucleic acid-based assays are listed below. Table 3 shows the minimum necessary quantities per participant for **one EQA scheme and round**. The quantities correspond to the quantities to be distributed to the participating laboratories, and do not include the quantities necessary to perform homogeneity and stability testing, and value assignment by the EQA provider.

The current best source of historical samples would be trachoma screening centers in endemic countries.

Table 3

Sample type	Quantity per item per participant (for one EQA round)
Serum (or plasma)	20ul
DBS (or DSS)	30 x 10ul Trop Bio filter paper wheels

Commercialized kit, QC material needs

For one kit, depending on the kit specifications in terms of target and matrix, one of the following may apply

Sample type	Quantity per item (for one EQA round)
Serum (or plasma)	10ul per test plate
DBS (or DSS)	10 x 10ul Trop Bio filter paper wheels per test plate

References consulted for the *Chlamydia trachomatis* TSP

World Health Organization. Report of the 3rd Global Scientific Meeting on Trachoma. Johns Hopkins University, Baltimore, MA, 19–20 July 2010; Geneva:World Health Organization.

World Health Organization Strategic and Technical Advisory Group on Neglected Tropical Diseases. Design parameters for population-based trachoma prevalence surveys. 2018;WHO/HTM/NTD/PCT/2018.07.

https://www.who.int/publications/i/item/WHO-PBD-GET-00.8

L Senyonjo et al. Lessons learned for surveillance strategies for trachoma elimination as a public health problem, from the evaluation of approaches utilised by Guinea worm and onchocerciasis programmes: a literature survey. PLoS Negl Trop Dis 2021;15:e0009082.

G Satpathy et al. Chlamydial eye infections: current perspectives. Indian Journal of Ophthalmology 2017;65 :97-102.

RL Fernandes De Souza Meneghim et al. Flies as possible vectors of inflammatory trachoma transmission in a Brazilian municipality. Revista do Instituto de Medicina Tropical de Sao Paulo 2021;63:e66.

AW Solomon et al. Diagnosis and assessment of trachoma. Clin Microbiol Rev 2004;17:982-1011.

SJ Bhosai et al. Trachoma: an update on prevention, diagnosis, and treatment. CurrO pin Ophthalmol 2012;23:288-295.

B Thylefors et al. A simple system for the assessment of trachoma and its complications. Bull WHO 1987;65:477-483.

AW Solomon et al. A diagnostic instrument to help field graders evaluate active trachoma. Ophthalmic Epidemiol 2018;25:399-402.

EM Harding-Esch et al. Diagnostic accuracy of a prototype point-of-care test for ocular Chlamydia trachomatis under firld conditions in the Gambia and Senegal. PLoS Negl Trop Dis 2011;5:e1234.

VH Hu et al. In vivo confocal microscopy in scarring trachoma. Ophthalmology 2011;118:2138-2146.

S Gwyn et al. Comparison of platforms for testing antibodies to Chlamydia trachomatis antigens in the Democratic Republic of the Congo and Togo. Clinical Trial Sci Rep 2021;11:7225.

TR Derrick et al. DjinniChip : evaluation of a novel molecular rapid diagnostic device for the detection of Chlamydia trachomatis in trachoma-endemic areas. Parasit Vectors 2020;13:533.

KS O'Brien et al. Precision of the Abbott RealTime Assay in the detection of ocular Chlamydia trachomatis in a trachoma-endemic area of Ethiopia. Am J Trop Med Hyg 2020;103:234-237.

S Gwyn et al. Optimization of a rapid test for antibodies to the Chlamydia trachomatis antigen Pgp3. Diagn Microbiol Infect Dis 2019;93:293-298.

JS Kim et al. Community-level chlamydial serology for assessing trachoma elimination in trachoma-endemic Niger. PLoS Negl Trop Dis 2019;13:e0007127.

JT Grayston. Background and current knowledge of Chlamydia pneumoniae and atherosclerosis. J Infect Dis 2000;181:402-410.

A Pinsent et al. The utility of serology for elimination surveillance of trachoma. Nat Commun 2018;9:5444.

T Derrick et al. miRNAs that associate with conjunctival inflammation and ocular Chlamydia *trachomatis* infection do not predict progressive disease. Pathogens and Disease 2017;75:ftx016.

AI Zambrano et al. The World Health Organization Recommendations for Trachoma Surveillance, Experience in Nepal and added benefit of testing for antibodies to chlamydia trachomatis pgp3 protein: NESTS study. PLoS Negl Trop Dis 2016;10:e0005003.

EB Goodhew et al. CT694 and pgp3 as serological tools for monitoring trachoma programs. PLoS Negl Trop Dis 20212;6:e1873.

CH Roberts et al. Development and evaluation of a next-generation digital PCR diagnostic assay for ocular Chlamydia trachomatis infections. J Clin Microbiol 2013;51:2195–203.

E Harding-Esch et al. Costs of testing for ocular Chlamydia trachomatis infection compared to mass drug administration for trachoma in the Gambia: application of results from the PRET study. PLoS Negl Trop Dis 2015;9:e0003670.

B Van der Pol & CA Gaydos. A profile of the binx health io® molecular point-of-care test for chlamydia and gonorrhea in women and men. Expert Rev Mol Diagn 2021;21:861-868.

Y Zhou et al. Performance of point-of-care tests for the detection of chlamydia trachomatis infections: A systematic review and meta-analysis. EClinicalMedicine 2021;37:100961.

http://www.chlamydiabiobank.co.uk/

https://www.fda.gov/media/73988/download

https://www.fda.gov/media/74033/download

https://www.cdc.gov/healthywater/hygiene/disease/trachoma.html

https://www.trachoma.org/

https://www.who.int/teams/control-of-neglected-tropical-diseases/trachoma/diagnosis

https://www.nibsc.org/

https://www.eptis.org/

NM Fenn Buderer. Statistical methodology : I. Incorporating the prevalence of disease into the sample size calculation for sensitivity and specificity. Acad Emerg Med 1996;3:895-900.

Acronyms

DBS	Dry blood spot
DSS	Dry serum spot
DTAG-NTD	Diagnostic Technical Advisory Group for Neglected Tropical Diseases
ELISA	Enzyme linked immunosorbent assay
EQA	External Quality Assurance
GET	Global elimination of trachoma
IF	Immunofluorescence
LFA	Lateral flow assay
LPS	Lipopolysaccharide
MDA	Massive drug administration
MIF	Microimmunofluorescence
MOMP	Major outer membrane protein
MTA	Material transfer agreement
NAAT	Nucleic acid amplification test
PBMCs	Peripheral blood mononuclear cells
PCR	Polymerase chain reaction
POC	Point of care
PQA	WHO prequalification assessment
PVA	Polyvinyl-acetal
RDT	Rapid diagnostic test

SNP	Single nucleotide polymorphism
TMA	Transcription mediated amplification
QC	Quality control

Annex 1

Specifications of potential sources of biospecimens from published studies and from completed or ongoing clinical trials. Included are articles published after 2013 and clinical trials with at least 100 participants and completed after 2010.

Type of collection	Reference	Contact
2013-2021		
430 donors from Nepal<br>Serum and DBS samples	doi.org/10.1016/j.jim.2016.05.008	Diana Martin, CDC, hzx3@cdc.gov
500 children from Tanzania<br>Ocular swabs, serum and DBS samples	doi.org/10.1016/j.diagmicrobio.2018.11.001	Diana Martin, CDC, hzx3@cdc.gov
160 children from Guinea Bissau, DNA and RNA samples from conjunctival swabs	doi.org/10.1186/s13071-016-1308-9	Tamsyn Derrick, London School of Hygiene and Tropical Medicine, tamsyn.derrick@lshtm.ac.uk
1000 donors from Guinea Bissau, DNA samples from conjunctival swabs	doi.org/10.1186/s13071-017-2566-x	Anna Last, London School of Hygiene and Tropical Medicine, anna.last@lshtm.ac.uk
1100 children from the Gambia and Senegal, conjunctival swabs	doi.org/10.1186/s13071-019-3743-x	Emma Harding Esch, London School of Hygiene and Tropical Medicine, esch@lshtm.ac.uk
1800 donors from the Gambia,<br>DBS samples	doi.org/10.1038/s41598-017-15056-7	Stephanie Migchelsen, London School of Hygiene and Tropical Medicine, stephanie.migchelsen@lshtm.ac.uk

1900 children from Tanzania, conjunctival swabs and DBS samples	doi.org/10.1038/s41598-020-71833-x	Sheila West, Dana Center for Preventive Ophthalmology, Wilmer Eye Institute, John Hopkins School of Medicine, shwest@jhmi.edu
500 children from Tanzania, with follow-up timepoints, DNA and RNA samples from conjunctival swabs	doi.org/10.1093/femspd/ftx016	Tamsyn Derrick, London School of Hygiene and Tropical Medicine, tamsyn.derrick@lshtm.ac.uk
730 donors from Guinea Bissau, DNA samples from conjunctival swabs	doi.org/10.1093/femspd/ftx050	Anna Last, London School of Hygiene and Tropical Medicine, anna.last@lshtm.ac.uk
140 children from Tanzania, DNA samples from conjunctival swabs	doi.org/10.1371/journal.pntd.0002265	Sheila West, Dana Center for Preventive Ophthalmology, Wilmer Eye Institute, John Hopkins School of Medicine, shwest@jhmi.edu
1160 donors from Ethiopia and Tanzania, with follow-up timepoints, DNA and RNA samples from conjunctival swabs	doi.org/10.1371/journal.pntd.0003763	Matthew Burton, London School of Hygiene and Tropical Medicine, matthew.burton@lshtm.ac.uk
50 children from Tanzania, RNA from conjunctival swabs and DBS samples	doi.org/10.1371/journal.pntd.0004352	Sheila West, Dana Center for Preventive Ophthalmology, Wilmer Eye Institute, John Hopkins School of Medicine, shwest@jhmi.edu

1300 donors from Ghana, conjunctival swabs and DBS samples	doi.org/10.1371/journal.pntd.0007027	Laura Senyonjo, Sightsavers UK, lsenyonjo@sightsavers.org
1000 donors from Niger, conjunctival swabs and DBS samples	doi.org/10.1371/journal.pntd.0007127	Diana Martin, CDC, hzx3@cdc.gov
450 children from Tanzania, with follow-up timepoints, conjunctival swab samples	doi.org/10.1371/journal.pntd.0007638	Athumani Ranadhani, London School of Hygiene and Tropical Medicine, althumani.ramadhani@lshtm.ac.uk
2900 children from Ghana, conjunctival swabs and DBS samples	doi.org/10.1371/journal.pntd.0009744	Laura Senyonjo, Sightsavers UK, lsenyonjo@sightsavers.org
2200 children from Tanzania, conjunctival swabs and DBS samples	doi.org/10.1038/srep18532	Diana Martin, CDC, hzx3@cdc.gov
420 donors from Nepal, serum samples	doi.org/10.4269/ajtmh.17-0292	Diana Martin, CDC, hzx3@cdc.gov
200 children from Ethiopia, conjunctival swab samples	doi.org/10.4269/ajtmh.19-0695	Jeremy Keenan, Francis Proctor Foundation, University of California, Jeremy.keenan@ucsf.edu
300 children from Tanzania, conjunctival swab samples	doi.org/10.1128/JCM.00519-13	Laura Dize, John Hopkins University, ldize2@jhmi.edu

FIND Diagnosis for all

29 000 participants,<br>Conjunctival swabs/DNA,<br>Ethiopia	https://clinicaltrials.gov/ct2/show/study/NCT01202331?recrs=ae&cond=Trachoma&draw=2&rank=11	Thomas Lietman, University of California, San Francisco, Tom.Lietman@ucsf.edu
20 000 participants,<br>Conjunctival swabs/DNA,<br>Ethiopia	https://clinicaltrials.gov/ct2/show/NCT00221364?recrs=ae&cond=Trachoma&draw=2&rank=10	Thomas Lietman, University of California, San Francisco, Tom.Lietman@ucsf.edu
1 100 participants,<br>Conjunctival swabs/DNA,<br>Niger	https://clinicaltrials.gov/ct2/show/NCT00618449?recrs=ae&cond=Trachoma&draw=2&rank=9	Julius Schachter, University of California, San Francisco (deceased); Abdou Amza, Programme National de Lutte Contre la Cécité, amzaabdou@gmail.com<br>dr.amzaabdou@gmail.com
120 participants,<br>Conjunctival swabs/DNA,<br>Niger, Tanzania, The Gambia	https://clinicaltrials.gov/ct2/show/NCT00792922?recrs=ae&cond=Trachoma&draw=2&rank=4	Sheila West, John Hopkins University, shwest@jhmi.edu
220 000 participants, with 7 follow-up timepoints,<br>Conjunctival swabs/DNA,<br>Ethiopia	https://clinicaltrials.gov/ct2/show/NCT02754583?recrs=ae&cond=Trachoma&draw=2&rank=2	Dionna Wittberg and Jeremy Keenan, University of California, San Francisco<br>dionna.wittberg@ucsf.edu<br>jeremy.keenan@ucsf.edu
100 000 participants,<br>Conjunctival swabs/DNA,	https://clinicaltrials.gov/ct2/show/NCT04185402?recrs=ae&cond=Trachoma&draw=2&rank=1	Catherine Oldenburg and Ariana Austin, University of California, San Francisco catherine.oldenburg@ucsf.edu

Niger		ariana.austin@ucsf.edu

LEPROSY, TSP Report

Introduction

Leprosy is caused by the intracellular and non-cultivable bacterium *Mycobacterium leprae* that infects the Schwann cells of the peripheral nervous system. *M. leprae*'s cell wall mainly consists of glycolipids, of which the most abundant one is the species-specific PGL-1. Leprosy can be incubated for up to 11 years, have different clinical presentations and be pauci-bacillary (PB) (≤5 lesions) or multi-bacillary (MB) (>5 lesions). A classification based on objective immunological criteria (Ridley-Jopling criteria) distinguishes between (i) tuberculoid (TT), borderline tuberculoid (BT), in a PB context, and (ii) mid-borderline (BB), borderline lepromatous (BL) and lepromatous (LL) leprosy, in a MB context. PB leprosy can also be indeterminate (I) or pure neuritic (PN). *Mycobacterium lepromatosis* is a different species, causing diffuse lepromatous leprosy (DLL).

The WHO recommended treatment consists of rifampicin, clofazimine, dapsone, minocycline and ofloxacin, varying between single dose and yearly treatments, depending on the clinical presentation. The lack of new diagnostic tools is highlighted in the WHO Global Leprosy Strategy 2016-2020. Most importantly, detection of early signs of leprosy, which is a strategy to stop the transmission, remains a challenge and there is no diagnostic test to detect/predict leprosy before the onset of clinical symptoms.

A *Mycobacterium leprae* virtual biobank is expected to support findability and availability of biospecimen panels for (i) EQA programs (or equivalent inter-laboratory exercises), reference and quality control material production (ii) method validation, including evaluation and/or PQA, and (iii) R&D other than validation of the analytical and clinical performance characteristics of a diagnostic test. Such R&D includes the identification of novel diagnostic biomarkers and/or their preliminary evaluation.

Scope

This document is intended as a description of the needs of biological materials in terms of *Mycobacterium leprae* panels.

The scope of this document includes the needs of biospecimens for development, then validation of molecular biology (nucleic acid based) assays, and assays based on detection or measurement of analytes, other than nucleic acids, found in biological fluids (serum, plasma, whole blood, urine, swab medium). Assays intended to be used both in a context of detection of subclinical *M. leprae* infections and in a context of confirming clinical leprosy are in the scope. Assays based on host-derived biomarkers and *Mycobacterium leprae* –derived biomarkers are also in the scope.

The following are out of the scope of this document: High-Resolution Ultrasonography, electro-neuromyography, skin bacilloscopy and histopathological examination assays. Skin tests for delayed type hypersensitivity reactions are also out of the scope.

Sources

The content of this report is based on information found in the **References,** and consultation with the **DTAG** on leprosy. Periodic revision can be made as information on ongoing and scheduled diagnostic development projects, funded by different donors becomes available.

Clinical and biological diagnosis of leprosy

The gold standard for the diagnosis of leprosy is clinical and based on presence of (i) hypo-pigmented or erythematous anesthetic patch of skin, (ii) thickened and/or tender peripheral or cutaneous nerve supplying the affected area, (iii) AFB in skin smear.

Apart from the clinical symptom-based diagnosis, and the lepromin skin tests (Fernandez reaction, Mitsuda reaction), direct biological diagnosis of leprosy is based on histopathological examination of slit skin tissue smears, IHC staining, FISH or NAAT. The antigen for lepromin skin tests (e.g. Armadillo-derived *M. leprae*, *M. leprae* cell wall antigen for Fernandez reaction / Mitsuda reaction), is not readily available. Slit skin smears is the WHO-recommended type of sample and slit skin smears from 4 sites are usually recommended. However, slit skin smears require 10^4 bacilli per gram tissue for reliable detection.

The current status of indirect diagnosis, based on serological or T cell assays, is described below. In the future, combinatorial diagnostics, including both host-based biomarkers (e.g. pro- or anti-inflammatory cytokines and/or acute phase proteins in plasma, lipid metabolites in serum/plasma or urine, gene expression signatures in skin tissue, whole blood or PBMCs, T-cell responsiveness to specific antigens) and *M. leprae*-based biomarkers (eg. *M. leprae* gene transcripts, *M. leprae* DNA) can possibly be developed and validated for the diagnosis of asymptomatic carriers.

Important biospecimen annotations

- Necessary annotation for *Mycobacterium leprae*: standardized dermatological examination data (e.g. hypo/hyper-pigmentation; results of thermal, tactile and pain sensitivity tests); standardized neurological examination data (e.g. evaluation of the eyes, nose, hands and feet, palpation of peripheral nerve trunks, muscle strength assessment, evaluation of sensation in eyes and upper and lower limbs); Ziehl-Neelsen stain results for presence of AFB in skin smears; MB/PB classification
-

- Useful annotation for *Mycobacterium leprae*: standardized histopathological examination results of skin biopsy; Ridley-Jopling classification; bacteriological index, Mitsuda reaction result.

Nucleic acid based (molecular biology) assays

End point or quantitative PCR of DNA extracted from slit skin smears, blood, sputum, oral or nasal or ocular swab medium, urine or other types of specimens, allows laboratories to diagnose *M. leprae,* enabling early identification. However, sensitivity is still lower in PB than in MB patients. Quantitative reverse transcriptase PCR of RNA may allow laboratories to diagnose viable *M. leprae*. Targets that have been used to date include the 16SrRNA, sodA, 36-kDa, Ag85B, 18-kDa, 65-kDa antigen coding genes or the *RLEP* repetitive sequences. Quantitative PCR could be used in the future to determine a Molecular Bacteriological Index (MBI).

Critical preanalytical factors for slit skin smears include the type of solution that is used to collect the specimen (e.g. 70% ethanol or other stabilizer) and the DNA or RNA extraction kit or method used. Critical preanalytical factors for fluid biospecimens include time and temperature between collection and start of processing, time and temperature between end of processing and storage, long term storage temperature, and DNA or RNA extraction kit or method used.

Non nucleic acid based (serological or cellular) assays

Serological diagnosis of leprosy, using serum/plasma or DBS, can be based on IgM anti-PGL-1 antibodies. Serological assays for these antibodies do not discriminate between symptomatic and asymptomatic individuals and are not very sensitive in identifying latent/early leprosy. However, the titers may be useful in predicting higher risk of developing leprosy or monitoring treatment efficacy. Other antigens that are commonly used are LID-1 and the ND-O-BSA. Presence of anti-LID-1 antibodies allows diagnosis 6-8 months before the onset of clinical symptoms. Presence of specific anti-ML0405 and anti-ML2331 antibodies may be indicative of pre-symptomatic leprosy. Other candidate antigens for serological assays include recombinant proteins Ag85B, ML2038, ML0286, ML2055, 46f and 92f, fusion proteins LID-1, NDO-LID or peptides. Testing for anti-LAM sIgA in saliva has recently been reported (Nahas 2018). ELISA, LFA and UCP-LFA are being used, mostly home-made.

Host-based candidate biomarkers include, not only serum proteins, but also serum or urinary metabolites, such as polyunsaturated fatty acids.

For antibody-based assays, critical preanalytical factors include the long-term storage conditions. For protein and/or metabolite-based assays, critical preanalytical factors include the type of anticoagulant, the time and temperature between collection and start of processing or analysis, the centrifugation conditions, the time and temperature between end of processing and cryopreservation, the long-term storage conditions.

T cell-based assays, using whole blood and IFNγ secretion as a read-out, have been developed against proteins, such as ESAT-6 (ML0049), ML0276, ML0840, ML1623, ML2044, ML0410, ML1053, ML1989, ML2283, 46f.

For T cell-based assays, critical preanalytical factors include the type of anticoagulant and type of blood collection tube, the time and temperature between collection and start of processing or analysis, the cryopreservation protocol, the cryopreservation solution.

Potential cross reactivities (important to inform the needs for validation of specificity, as relevant, depending on the analyte):

When performing serology for anti-*M. leprae* antibodies, or T cell-based assays with *M. leprae* antigens, which have homologues in other mycobacterial species, specificity should be established against *M. tuberculosis, M. paratuberculosis, M. intracellulare, M. avium,* and BCG.

Table 1 gives the different contexts of use of diagnostic tests and corresponding sample types and method types.

Table 1

Context of use	Sample type and method
Confirmation of clinical leprosy	Slit skin smear, skin biopsy, nasal swabs, whole blood, urine, saliva (NAAT)<br>Serum/plasma, urine or DBS (IgG/IgM ELISA or other biomarkers)<br>Saliva (sIgA ELISA)
Detection of subclinical leprosy	Slit skin smear, skin biopsy, nasal swabs, whole blood, urine, saliva (NAAT)<br>Serum/plasma, urine or DBS (IgG/IgM ELISA or other biomarkers)<br>Saliva (sIgA ELISA)

Need for samples corresponding to different *Mycobacterium leprae* strains:

The main relevant geographical area for *Mycobacterium leprae* biospecimen collection is Southeast Asia (India, Indonesia, Bangladesh), while low and focal prevalence is observed in

South America (Brazil) and Africa (DRC, Madagascar, Mozambique, Ethiopia, Nigeria, Tanzania). *M. lepromatosis* is mainly found in Mexico and the Caribbean region.

VNTR and SNP polymorphisms exist between different *M. leprae* strains, and when used as PCR targets, they can differentiate between strains. Therefore, collection of samples from different geographical areas is important only if the analytical target is one or more of these polymorphic genetic loci.

Reference methods:

- Clinical diagnosis with slit skin microscopy

Reference materials

No WHO international standard exists for leprosy.

A recombinant IgG4 antibody has been proposed as a quality control material by PATH (Golden et al. 2016).

BEI Resources offer M. *leprae* nucleic acids, antigen preparations, and polyclonal antiserum materials, for research purposes, https://www.beiresources.org/Catalog.aspx?f_instockflag=In+Stock%23~%23Temporarily+Out+of+Stock&q=leprae

FDA reference panels

No FDA reference panel for leprosy could be found.

Validated (FDA approved) methods

No FDA approved diagnostic method could be found.

Other methods that have been described/made available

- ML Flow (lateral flow) (OmegaTeknika Limited, Ireland) (as per Bührer-Sekula 2003), based on a semi-synthetic trisaccharide of the PGL-1
- ML Flow test (Kit Biomedical, The Netherlands)
- ML ICA (Standards Diagnostics Inc, Republic of Korea), based on a semi-synthetic disaccharide of the PGL-1
- NDO-LID Rapid Test (Orange Life, Brazil; bought by ChemBio), based on a semi-synthetic PGL-1 mimic, conjugated with fusion proteins ML0304 and ML0331.

- OnSite Leprosy Ab Rapid test (CTK Biotech, San Diego, CA)
- Human *Mycobacterium leprae* heat shock protein 65kDa (HSP65) ELISA kit (MyBioSource)

- *Mycobacterium leprae* and *Mycobacterium lepromatosis*, PCR kit (MyBioSource), discontinued
- HELINI *Mycobacterium leprae* real-time PCR kit
- Leprosy RNA polymerase beta subunit (rpoB) gene Genesig standard kit (Genesig)
- *Mycobacterium leprae* (one step) PCR (Genekam Biotechnology AG)

No information on the composition of the panels that have been used for method validation by the companies could be found for any of these RDT or PCR tests.

EQA programs

The only available EQA program that could be found on the EPTIS website is a scheme based on digitalized cases of smears, fixed and stained by the cold Ziehl-Neelsen method, prepared from intradermal scrapes from 4 different collection sites (ControlLab Controle de Qualidade para Laboratórios Ltda, Brazil).

Panel needs

R&D / method validation needs

Table 2a shows the needs in terms of panels for initial development /feasibility studies, for both nucleic acid-based and non-nucleic acid-based assays.

Whole blood for cellular immunity tests can only be used fresh, in less than 3 hours after collection, and at least 5-8ml whole blood is needed. Therefore, it is not possible to use historical whole blood samples for purposes of method development or validation.

Table 2a

Sample type	Quantity per donor (for 1 development panel)	Number of biospecimen donors
Serum/ plasma	1ml	30 *M. leprae* symptomatic positive cases<br>30 *M. leprae* asymptomatic positive cases<br>30 *M. leprae* negative cases
Urine	2ml	30 *M. leprae* symptomatic positive cases<br>30 *M. leprae* asymptomatic positive cases<br>30 *M. leprae* negative cases
Nasal swab medium	2ml	30 *M. leprae* symptomatic positive cases<br>30 *M. leprae* asymptomatic positive cases<br>30 *M. leprae* negative cases
Saliva	1ml	30 *M. leprae* symptomatic positive cases<br>30 *M. leprae* asymptomatic positive cases<br>30 *M. leprae* negative cases
Whole blood or DBS	0,4ml WB or 2 DBS	30 *M. leprae* symptomatic positive cases<br>30 *M. leprae* asymptomatic positive cases<br>30 *M. leprae* negative cases

Slit skin samples, frozen, stabilized or fixed (from earlobes, elbows and/or knees)	2 samples	30 *M. leprae* symptomatic positive cases<br>30 *M. leprae* negative cases

Table 2b shows the needs in terms of complete validation panels for each indication and for both nucleic acid-based and non-nucleic acid-based assays. Numbers of donors are defined based on the WHO TPP minimum sensitivity and specificity requirements. The numbers provided correspond to the needs for clinical validation. It is expected that part of these samples will be used, either individually, or pooled (according to the analytical validation plan) for analytical validation purposes too.

For NAAT method validation using skin snips as the matrix, 2mg skin snips (equivalent to 3mm diameter) would be needed.

Table 2b

Sample type	Quantity per donor (for 1 validation panel)	Number of biospecimen donors
Serum/ plasma	0,5ml	600[(1)] *M. leprae* symptomatic positive cases<br>600[(2)] *M. leprae* asymptomatic positive cases<br>Mix of 14820 [(1)]/6990[(2)] cases, *M. leprae* negative, of which half being positive for any of the following: *M. tuberculosis, M. paratuberculosis, M. intracellulare, M. avium,* PKDL
Urine	1ml	600[(1)] *M. leprae* symptomatic positive cases<br>600[(2)] *M. leprae* asymptomatic positive cases<br>Mix of 14820 [(1)]/6990cases, *M. leprae* negative, of which half being positive for any of the following: *M. tuberculosis, M. paratuberculosis, M. intracellulare, M. avium,* PKDL
Nasal swab medium	0,5ml	600[(1)] *M. leprae* symptomatic positive cases<br>600[(2)] *M. leprae* asymptomatic positive cases<br>Mix of 14820 [(1)]/6990cases, *M. leprae* negative, of which half being positive for any of the following: *M. tuberculosis, M. paratuberculosis, M. intracellulare, M. avium,* PKDL

Saliva	0,5ml	600[(1)] *M. leprae* symptomatic positive cases<br>600 [(2)] *M. leprae* asymptomatic positive cases<br>Mix of 14820 [(1)]/6990cases, *M. leprae* negative, of which half being positive for any of the following: *M. tuberculosis, M. paratuberculosis, M. intracellulare, M. avium,* PKDL
Whole blood or DBS	0,2ml WB or 1 DBS	600[(1)] *M. leprae* symptomatic positive cases<br>600[(2)] *M. leprae* asymptomatic positive cases<br>Mix of 14820 [(1)]/6990cases, *M. leprae* negative, of which half being positive for any of the following: *M. tuberculosis, M. paratuberculosis, M. intracellulare, M. avium,* PKDL
Slit skin samples, frozen, stabilized or fixed (from earlobes, elbows and/or knees)	1 sample	600[(1)] *M. leprae* symptomatic positive cases<br>600[(2)] *M. leprae* asymptomatic positive cases<br>Mix of 14820 [(1)]/6990cases, *M. leprae* negative, of which half being positive for any of the following: *M. tuberculosis, M. paratuberculosis, M. intracellulare, M. avium,* PKDL

[1] applicable to a context of confirmation of clinical leprosy (assumed prevalence in the target population 2.5%, expected sensitivity 90% and expected specificity 99% (null values), alternative values 93% and 99.2% respectively, desired precision 10%). True value of assay performance is unknown and chosen alternative values might be challenging to attain. Performance closer to null value would require larger sample sizes.
[2] applicable to a context of detection of subclinical leprosy (assumed prevalence in the target population 2.5%, expected sensitivity 81%, expected specificity 99,5% (null values), alternative values 85% and 99.7% respectively, desired precision 10%). True value of assay performance is unknown and chosen alternative values might be challenging to attain. Performance closer to null value would require larger sample sizes.

Positivity status is an attribute of the donor.

Positivity for symptomatic leprosy has been traditionally defined by clinical symptoms (hypo-pigmented or erythematous anesthetic patch of skin and/or thickened and/or tender peripheral or cutaneous nerve supplying the affected area), and positive AFB in skin smear.

Positivity for asymptomatic leprosy may be defined by contact tracing and positive NAAT. This definition may lead to much higher prevalence in endemic regions than the assumed 2.5%.

Required number of positive/negative samples have been estimated using a two-sided binomial test, for the desired diagnostic sensitivity and specificity levels, using Nquery 8.4 software at an target significance level 0.05 or 0.1 and with 80% power to detect a change in

sensitivity from 81% to 85% or a change in specificity from 99.5% to 99.7% for detection of subclinical infection, and a change in sensitivity from 90% to 93% or a change in specificity from 99% to 99.2% for confirmation of clinical infection.

Complete biospecimen sets, including all biospecimen types, from the same donor are preferred. It is critical that for a validation panel, all biospecimens come from the same collection, to avoid preanalytical bias. Preanalytical bias is unavoidable if using biospecimens from different collections with different or undocumented preanalytical specifications.

Possible retrospective sources of specimens can be found in published literature and in completed or ongoing clinical trials (Annex 1). The most suitable sources for identification and validation of colonization stage-specific and/or early disease-specific biomarkers would be **longitudinal** collections, with sampling of household contacts of lepromatous patients.

Although the first priority is the needs for development, then for validation of new methods, in order for a new method to be deployed in the field, an external quality assurance (EQA) program is needed, as well as quality control (QC) materials to be included in the kits.

EQA needs

The needs for EQA panels for non-nucleic acid-based assays are listed below. Table 3 shows the minimum necessary quantities per panel for **one EQA scheme and round** for **40 participating laboratories**, with each laboratory receiving 1 DBS. The quantities correspond to the quantities to be distributed to the participating laboratories, supplemented by the quantities necessary to perform homogeneity and stability testing, and value assignment by the EQA provider.

The current best source of historical samples would be leprosy screening centers in endemic countries.

Table 3

	Sample type	Quantity per item (for 1 EQA round)	Number of items
Non nucleic acid based	DBS	60 spots	3 items *M. leprae* positive<br>1 items *M. leprae* and *M. lepromatosis* negative

Note. Slit skin items for nucleic acid-based methods cannot be procured. The only possible item for NAAT EQA would be already extracted DNA (or RNA) samples with different copy number concentrations.

Commercialized kit, QC material needs

For one kit using serum or plasma,

- At least 500ml of pooled *M. leprae* serum/plasma

References consulted for the *Mycobacterium leprae* TSP

https://apps.who.int/iris/bitstream/handle/10665/208824/9789290225096_en.pdf?sequence=14&isAllowed=y

https://www.leprosy-ila.org/do.php/Home

http://www.turingfoundation.org/lepra07_uk.html

https://ilepfederation.org/wp-content/uploads/2020/02/LG3.pdf

MR Mungroo et al. *Mycobacterium leprae*: pathogenesis, diagnosis, and treatment options. Microb Pathogenesis 2020;149:104475.

J Gaschignard et al. Pauci- and multibacillary leprosy: two distinct, genetically neglected diseases. PLoS Ngl Trop Dis 2016;10:e0004345.

P Steinmann et al. Innovative tools and approaches to end the transmission of Mycobacterium leprae. Lancet Infect Dis 2017;17:e298-e305.

MS Duthie et al. Use of protein antigens for early serological diagnosis of leprosy. Clin Vaccine Immunol 2007;14:1400-1408.

MS Duthie et al. Antigen specific T cell responses of leprosy patients. Clin Vaccine Immunol 2008;15:1659-1665.

G Sarode et al. Epidemiological aspects of leprosy. Disease-a-Month 2019;15:14.

DS Ridley & WH Jopling. Classification of leprosy according to immunity. A five group system, Int J Lepr Other Mycobact Dis 1966;34:255-273.

I Nath et al. Immunology of leprosy and diagnostic challenges. Clinics Dermatol 2015;33:90-98.

P Qiong-Hua et al. Early revelation of leprosy in China by sequential antibody analyses with LID-1 and PGL-1. J Trop Med 2013;2013:352689.

JS Spencer et al. Identification of serological biomarkers of infection, disease progression and treatment efficacy for leprosy. Mem Inst Oswaldo Cruz 2012;107:79-89.

EM Hungria et al. Seroreactivity to new *Mycobacterium leprae* protein antigens in different leprosy endemic regions in Brazil. Mem Inst Oswaldo Cruz 2012;107:104-111.

A da Conceicao Oliveira Coelho Fabri et al. Integrative literature review of the reported uses of serological tests in leprosy management. Revista da Sociedade Brasileira de Medicina Tropical 2016;49:158-164.

S Bührer Sekula et al. Simple and fast lateral flow test for classification of leprosy patients and identification of contacts with high risk of developing leprosy. J Clin Microbiol 2003;41:1991-1995.

Y Luo et al. Host-related laboratory parameters for leprosy reactions. Front Med 2021;8:694376.

R Silva Gama et al. Prospects for new leprosy diagnostic tools, a narrative review considering ELISA and PCR assays. J Brazilian Soc Trop Med 2020;53:e20200197.

AN Martinez et al. Molecular determination of *Mycobacterium leprae* viability by use of real-time PCR. J Clin Microbiol 2009;47:2124-2130.

OA Espinoza et al. Accuracy of enzyme-linked immunosorbent assays (ELISA) in detecting antibodies against Mycobacterium leprae in leprosy patients: a systematic review and meta-analysis. Can J Med Microbiol Inf Dis 2018:9828023.

A Kumar et al. Analysis of antigens of *Mycobacterium leprae* by interaction to sera IgG, IgM, and IgA response to improve diagnosis of leprosy. Biomed Res Int 2014;:283278.

JHM van Dijk et al. Synthetic phenolic glycolipids for application in diagnostic tests for leprosy. ChemBioChem 2021;22:1487-1493.

K Bobosha et al. Field evaluation of a new lateral flow assay for detection of cellular and humoral immunity against *Mycobacterium leprae*. PLoS Negl Trop Trop Dis 2014;8:e2845.

AA Nahas et al. Anti.lipoarabinomannan-specific salivary IgA as prognostic marker for leprosy reactions in patients and cellular immunity in contacts. Front Immunol 2018;9:1205.

A van Hooij & A Geluk. In search of biomarkers for leprosy by unraveling the host immune response to Mycobacterium leprae. Immunol Rev 2021;301:175-192.

S Gautam et al. Insights into Mycobacterium leprae proteomics and biomarkers-An overview. Proteomes 2021;9:7.

OA Mayboroda et al. Exploratory urinary metabolomics of type 1 leprosy reactions. Int J Infect Dis 2016;45:46-52.

A van Hooij et al. Application of new host biomarker profiles in quantitative point-of-care tests facilitates leprosy diagnosis in the field. EBioMedicine 2019;47:301-308.

A Nobrega Martinez et al. PCR-based techniques for leprosy diagnosis: from the laboratory to the clinic. PLoS Negl Trop Dis 2014;8:e2655.

S Tatipally et al. Polymerase chain reaction (PCR) as a potential point of care laboratory test for leprosy diagnosis-A systematic review. Trop Med Inf Dis 2018;3:107.

https://cdn.mybiosource.com/tds/protocol_manuals/000000-799999/MBS486367_Advanced.pdf

https://www.helini.in/uploads/3/0/9/5/30951267/helini_mycobacterium_leprae_real-time_pcr_kit.pdf

https://www.cdc.gov/leprosy/health-care-workers/laboratory-diagnostics.html

https://apps.who.int/iris/bitstream/handle/10665/274127/9789290226383-eng.pdf

https://www.nibsc.org/

https://www.fda.gov/medical-devices/device-advice-comprehensive-regulatory-assistance/medical-device-databases

https://www.eptis.org/

NM Fenn Buderer. Statistical methodology : I. Incorporating the prevalence of disease into the sample size calculation for sensitivity and specificity. Acad Emerg Med 1996;3:895-900.

Acronyms

AFB	Acid fast bacilli
BB	Mid-borderline
BCG	Bacillus Calmette-Guérin vaccine
BL	Borderline lepromatous
BSA	Bovine serum albumine
BT	Borderline tuberculoid
DBS	Dry blood spot
DLL	Diffuse lepromatous leprosy
DRC	Democratic Republic of Congo
DTAG-NTD	Diagnostic Technical Advisory Group for Neglected Tropical Diseases
ELISA	Enzyme linked immunosorbent assay
ENL	Erythema nodosum leprosum
ESAT	Early secretory antigen target
EQA	External Quality Assurance
FISH	Fluorescence in situ hybridization
I	Indeterminate
IHC	Immunohistochemical
LAM	Lipoarabinomannan
LFA	Lateral flow assay
LL	Lepromatous

LID-1	Lipid droplet protein 1
MB	Multibacillary
MBI	Molecular bacteriological index
MTA	Material transfer agreement
NAAT	Nucleic acid amplification test
NDO	Natural disaccharide octyl
PB	Paucibacillary
PBMCs	Peripheral blood mononuclear cells
PCR	Polymerase chain reaction
PGL-1	Phenolic glycolipid 1
PKDL	Post kalaazar dermal leishmaniasis
PN	Pure neuritic
POC	Point of care
PQA	WHO prequalification assessment
RDT	Rapid diagnostic test
SNP	Single nucleotide polymorphism
TT	Tuberculoid
QC	Quality control
UCP	Up-converting particles (or up-converting phosphor conjugates)
VNTR	Variable nucleotide tandem repeats

Annex 1

Specifications of potential sources of biospecimens from published studies and from completed or ongoing clinical trials. Included are articles published after 2013 and clinical trials with at least 100 participants and completed after 2010.

Type of collection	Reference	Contact
2013-2018		
240 donors from Brazil<br>DBS samples	doi.org/10.1371/journal.pntd.0006083	Annemieke Geluk, Leiden University Medical Center, a.geluk@lumc.nl
400 donors from Brazil<br>Serum samples	doi.org/10.1590/0074-02760160505	Ana Paula Mendes Carvalho, Universidade Federal de Minas Gerais, anapaulamcarvalho@yahoo.com.br
540 donors from Colombia<br>Slit skin smears, nasal swabs, serum samples, bacterial DNA	doi.org/10.1371/journal.pntd.0005325	Nora Cardona-Castro, ncardona@ces.edu.co
2500 donors from Brazil<br>Slit skin smears, biopsies, serum samples	doi.org/10.1093/trstmh/tru093	Eduardo Netto, Federal University of Bahia, enetto@ufba.br
330 donors from Philippines and Bangladesh<br>Serum samples	doi.org/10.1038/s41598-017-07803-7	Annemieke Geluk, Leiden University Medical Center, a.geluk@lumc.nl
800 donors from Brazil<br>Serum samples, bacterial DNA	doi.org/10.1111/1469.12349	S. Araujo, Federal University of Uberlandia, sergiooxwide@hotmail.com

FIND Diagnosis for all

2019-2021		
460 donors from Brazil<br>Slit skin smear, serum samples	doi.org/10.1371/journal.pone.0251631	John Spencer, Colorado State University, john.spencer@colostate.edu
780 donors from Brazil<br>Whole blood and serum samples	doi.org/10.1186/s12879-018-3653-0	André Luiz Leturiondo, Universidade do Estado do Amazonas, Brazil, andre_leturiondo@yahoo.com.br
180 donors from Bangladesh<br>Plasma samples	doi.org/10.1016/j.ebiom.2019.08.009	Annemieke Geluk, Leiden University Medical Center, a.geluk@lumc.nl
23 donors from Brazil<br>Skin scrapings, skin biopsies, whole blood, DBS, oral swabs, nasal swabs, skin lesion swabs, body hair.	doi.org/10.1371/journal.pntd.0008325 M	Milton Moraes, Instituto Oswaldo Cruz, Fiocruz, Rio de Janeiro, milton.moraes@fiocruz.br
189 donors from Brazil<br>Whole blood samples	doi.org/10.1111/jam.14592	Rachel Caligiorne, Hospital Santa Casa de Belo Horizonte, rachelbc@santacasabh.org.br
300 donors from Bangladesh<br>Slit skin smears, nasal swab samples, bacterial DNA	doi.org/10.3389/fmicb.2020.01220	Annemieke Geluk, Leiden University Medical Center, a.geluk@lumc.nl

6 cases *M. leprae/M. tuberculosis* coinfection from Sri Lanka	doi.org/10.1186/s13256-020-02413-w	National Hospital of Sri Lanka,<br>Tharukaherath11@gmail.com
80 donors from India<br>Slit skin samples, bacterial DNA	doi.org/10.22099/mbrc.2020.35658.1464	Partha Sarathi Mohanty, National JALMA Institute for Leprosy and Other Mycobacterial Diseases, Agra, mohanty.ps@icmr.gov.in m.sarathipartha@gmail.com
900 participants, Skin biopsy (non facial), Nasal swabs, Fingerstick blood and Slit skin smears (non facial and only for multibacillary patients), Comoros	https://clinicaltrials.gov/ct2/show/NCT03526718?recrs=ae&cond=Leprosy&draw=2&rank=2	Bouk de Jong,<br>Inst. Tropical Medicine, Belgium
300 participants, blood, skin scrapings, nasal swabs and biopsies, bacterial DNA and mRNA,<br>Philippines	https://clinicaltrials.gov/ct2/show/NCT00315809?recrs=ae&cond=Leprosy&draw=2&rank=3	Varalakshmi Vissa, Colorado State University,<br>Maria Felicio-Balagon, Leonard Wood Memorial, Cebu, Phillippines
370 participants,<br>skin biopsy or dermal smear or nasal swab, bacterial DNA<br>French Guiana	https://clinicaltrials.gov/ct2/show/NCT05031091?recrs=ae&cond=Leprosy&draw=3&rank=11	Pierre Couppie, Centre Hospitalier de Cayenne, mathieu.nacher@ch-cayenne.fr, roxane.schaub@ch-cayenne.fr

1500 participants, blood, nasal swabs, slit skin smears, and biopsies, bacterial DNA and mRNA,<br><br>Colombia	https://clinicaltrials.gov/ct2/show/NCT00138437?recrs=ae&cond=Leprosy&draw=2&rank=12	Varalakshmi Vissa, Colorado State University, and Nora Cardona-Castro, Instituto Colombiano de Medicina Tropical - Universidad CES

Lassa virus, TSP Report

Introduction

The Lassa virus (LASV) is a hemorrhagic, single-stranded RNA arenavirus, endemic in West Africa. The first reported outbreak was in Nigeria in 1969. The rat *Mastomys natalensis* is the natural host of the virus. The annual incidence is around 300 000 cases and mortality rates are around 2%. Infection occurs by ingestion or inhalation of LASV-contaminated rat excreta. Person to person transmission via body secretions also occurs. LASV is a BSL-4 and one of the most pathogenic viruses. Symptoms appear two weeks after infection and are non-specific, with fever, headache, myalgia, chest pain, vomiting diarrhea. Roughly 20% of the patients require hospitalization.

The only currently available treatment option is ribavirin, which is effective at the onset of infection and may also be used for post-exposure prophylaxis.

Early diagnosis is critical to benefit from this antiviral therapy.

A Lassa virus virtual biobank is expected to support findability and availability of biospecimen panels for (i) EQA programs (or equivalent inter-laboratory exercises), reference and quality control material production (ii) method validation, including evaluation and/or PQA, and (iii) R&D other than validation of the analytical and clinical performance characteristics of a diagnostic test. Such R&D includes the identification of novel diagnostic biomarkers and/or their preliminary evaluation.

Scope

This document is intended as a description of the needs of biological materials in terms of Lassa virus panels.

The scope of this document includes the needs of biospecimens for development, then validation of

- molecular biology assays, based on detection or measurement of nucleic acids, found in swabs (e.g. throat, vaginal) or biological fluids (blood, urine, pleural fluid, semen, sputum, oral fluid, CSF)
- assays based on detection or measurement of analytes, other than nucleic acids, found in biological fluids (serum, plasma, whole blood).

Assays intended to be used both in a context of surveillance and diagnosis of Lassa virus infections are in the scope. Assays based on host-derived biomarkers are also in the scope.

The following are out of the scope of this document: immunohistochemistry on tissue samples, virus isolation, electron microscopy and serum neutralization tests with native LASV.

Sources

The content of this report is based on information found in the **References**.

Clinical and biological diagnosis of Lassa virus

Biological diagnosis is critical since clinical symptoms are not specific. Overall, the gold standard for the biological diagnosis of LASV is based on direct detection of the virus, its antigens or its RNA, or on serum neutralization as serological confirmatory test. The context for LASV diagnosis can be either surveillance or diagnosis of acute infection.

Because virus isolation and serum neutralization tests require BSL-4 facilities, these are very difficult to deploy. Virus inactivation by sample heating (60°C 60min) with or without chemical denaturation, e.g. with guanidine salts, has been documented (Blow, 2004; Haddock, 2016; Smither, 2015; Mitchell, 1984).

In a surveillance context, serum plasma or whole blood can be used for IgG detection. In a diagnostic context, a combination of direct antigen detection in any biological fluid or swab, and anti-LASV IgM antibodies in blood, can offer optimized sensitivity (orthogonal testing).

RNA, extracted from different types of biospecimens mentioned above can be useful for development of direct diagnostic assays.

Important biospecimen annotations

- Necessary annotations for Lassa virus: geographical origin, visible bleeding, headaches, fever, malaise, sore throat, myalgia, cough, chest pain, abdominal pain, nausea, vomiting, diarrhea, facial swelling, low blood pressure, bruising, fluid in the lungs.
- Useful annotations for Lassa virus: preanalytical data (time to centrifugation/freezing/stabilization, storage temperature), CBC, transaminases, alkaline phosphatase and blood urea nitrogen levels.

Nucleic acid based (molecular biology) assays

PCR, NASBA or LAMP assays can be used for direct detection of the viral RNA in blood, urine, pleural fluid, semen, sputum, CSF or throat swabs. Direct detection of LASV in urine or CSF can be more sensitive than in blood. Specific targets are the NP and GPC coding regions, but can also be the RNA polymerase (L) and the matrix protein (Z) RNA coding segments.

Critical preanalytical factors, for all types of fluid samples, include time and temperature between collection and start of processing, time and temperature between end of processing and storage, long term storage temperature, and RNA extraction kit or method used. Other critical preanalytical factors are the type of anticoagulant for whole blood and blood derivatives (buffy coat, serum, plasma), the centrifugation conditions and use of stabilizer for urine.

Non nucleic acid based (serological or cellular) assays

LASV immunogenic proteins include the nucleoprotein, the glycoprotein and the Z protein. LASV nucleoprotein antigen, a relatively conserved protein, is detectable the first week of illness, then its levels decrease while anti-LASV antibodies appear. LASV antigen levels quickly decrease in the course of infection, even if viremia persists.

Antibody detection is performed by ELISA, having replaced IFAs. Indirect ELISAs using viral antigens for serological testing are preferably based on recombinant rather than native proteins, to avoid the BSL-4 limitation.

Serological assays for IgM in serum can be used for diagnosis. In this context, blood samples can best be collected in the second week after the onset of symptoms for testing for IgM. IgM antibodies persist for many months or even years, making IgM seropositivity alone insufficient to diagnose acute infection.

Serological assays for IgG do not discriminate active from past infection either, since antibodies may persist for decades after infection. These can scarcely be used for surveillance purposes since in endemic areas, almost half of the healthy individuals have Lassa IgG antibodies. IgG antibodies generally appear three weeks after the onset of symptoms. Some fatal cases have been reported without appearance of antibodies at all.

For antibody-based assays, critical preanalytical factors include the long-term storage conditions. For antigen-based assays, critical preanalytical factors include the type of anticoagulant, the time and temperature between collection and start of processing or analysis, the centrifugation conditions, the time and temperature between end of processing and cryopreservation, the long-term storage conditions.

Potential cross reactivities (important to inform the needs for validation of specificity, as relevant, depending on the analyte).

Cross reactions against different LASV lineages often occur. This is not a problem as pan-Lassa diagnostic kits are a better option than lineage-specific ones.

Cross reactions may occur against New World arenaviruses (e.g. Machupo, Junin).

Assessment of cross reactivity against other hemorrhagic viruses (ebolavirus, marburgvirus, Crimean Congo hemorrhagic fever virus, Rift Valley fever virus, Dengue virus, and Yellow fever virus) and fever-associated viruses would be useful in the context of differential diagnosis.

Table 1 gives the different contexts of use of diagnostic tests and the corresponding most important sample types and method types.

Table 1

Context of use	Sample type and method
Surveillance for Lassa virus	Serum/plasma, DBS (IgG/IgM ELISA or other biomarkers)
Confirmation/diagnosis of Lassa virus	Blood, urine, oral fluid(LASV antigen capture test, NAAT or other biomarkers)

Need for samples corresponding to different Lassa virus strains:

The main relevant geographical area for Lassa virus biospecimen collection is Western African countries, e.g. Nigeria, Sierra Leone, Liberia, Guinea, Benin, Ghana, Mali. There are at least four different lineages: lineage I from Eastern Nigeria, lineage II from Southern Central Nigeria, lineage III from Northern Central Nigeria, lineage IV from Guinea, Liberia and Sierra Leone. Two other recently identified lineages originate from Mali and Togo, while sublineages have also been identified. Strain variation at the nucleotide level is as high as 25%, while variation at the amino acid level is up to 12%. It has been shown that the majority of positive serum samples have strain specific antibodies. Since LASV diversity clusters with geographic locations, collection of strains from different endemic regions is important for validation of the specificity of both nucleic acid-based and non-nucleic acid-based assays.

Reference methods:

- LASV isolation on Vero E6 cell line, preferably from blood or CSF samples, followed by sequencing or RT-PCR confirmation.

FIND Diagnosis for all

Reference materials

Two WHO international standards exist for Lassa virus

- a lyophilized, inactivated Josiah isolate suspension (https://nibsc.org/documents/ifu/21-112.pdf)
- a lyophilized suspension of lentivirus carrying synthetic LASV sequences (https://nibsc.org/documents/ifu/22-108.pdf)

FDA reference panels

No FDA reference panel for Lassa virus could be found.

Validated (FDA approved) methods

No FDA approved diagnostic method could be found.

Other methods that have been described/made available

RDT
- Corgenix, ReLASV antigen rapid test (CE IVD)
- Zalgen, ReLASV Pan-Lassa antigen rapid test (RUO). This test uses a mixture of polyclonal antibodies against recombinant nucleoproteins from different LASV strains.

ELISA
- Zalgen, ReLASV Pan-Lassa antigen ELISA test kit (RUO)
- Zalgen, ReLASV Pan-Lassa NP IgG/IgM ELISA test kit (RUO), specific to either GP or NP antigens
- MyBiosource (Clinisciences), Qualitative Human Lassa Virus IgG/IgM ELISA kits (RUO)
- An antibody capture immunoassay for detection of LASV nucleoprotein-specific antibodies has been developed (Gabriel, 2018).

NAAT
- Aldatu Biosciences, PANDAA qDx LASV (RUO)
- Altona Diagnostics, RealStar Lassa virus RT PCR kit 2.0 (RUO, CE IVD)
- LifeRiver (ZJ Bio-Tech), Lassa Virus (LV) Real Time RT-PCR Kit (CE-IVD)
- Bioperfectus, Lassa Fever virus real time PCR kit (RUO)
- Genesig, Lassa virus Josiah GP gene advanced kit (RUO)
- Mabsky, Lassa Fever virus detection kit
- A laboratory-developed one step RT-PCR has been described (Dedkov, 2019)

No information on the composition of the panels that have been used for method validation by the companies could be found for any of these RDT, ELISA or PCR tests.

MULTIPLEX TESTS

- Biofire, Biofire Global Fever Special Pathogens panel
- A laboratory-developed multiplex NAAT detects different viruses that cause hemorrhagic fever symptoms, including LASV (Das, 2015)
- A multiplex MAGPIX assay has been developed for detection of IgG against different hemorrhagic fever viruses (Satterly, 2017)

EQA programs

No EQA program for LAV could be found on the EPTIS website.

An EQA for molecular diagnosis of LAV was organized by the ENIVD using samples spiked with different inactivated LV strains (Nikinsis, 2015).

Panel needs

R&D / method validation needs

Table 2a shows the needs in terms of panels for initial development /feasibility studies, for both nucleic acid-based and non-nucleic acid-based assays.

Table 2a

Sample type	Quantity per donor (for 1 development panel)	Number of biospecimen donors
Serum/<br>plasma	1ml	20 Lassa virus serologically and NAAT positive cases<br>20 Lassa virus serologically and NAAT negative cases
Urine	2ml	20 Lassa virus serologically and NAAT positive cases<br>20 Lassa virus serologically and NAAT negative cases
Oral fluid	1ml	20 Lassa virus serologically and NAAT positive cases<br>20 Lassa virus serologically and NAAT negative cases

Whole blood or DBS	0,4ml WB or 2 DBS	20 Lassa virus serologically and NAAT positive cases<br>20 Lassa virus serologically and NAAT negative cases

Table 2b shows the needs in terms of complete validation panels for each indication and for both nucleic acid-based and non-nucleic acid-based assays. Numbers of donors are indicative as no TPP has been published for LASV.

Table 2b

Sample type	Quantity per donor (for 1 validation panel)	Number of biospecimen donors
Serum/ plasma	0,5ml	62(1) / 150(2) Lassa virus serologically and NAAT positive cases (including different LASV lineages)<br>62(1) / 280(2) Lassa virus serologicall and NAAT negative cases, including cased serologically and/or NAAT positive for any of the following: ebolavirus, marburgvirus, Crimean Congo hemorrhagic fever virus, Rift Valley fever virus, Dengue virus, and Yellow fever virus, or West Nile virus, RSV, measles, mumps, rubella, cytomegalovirus, Epstein Barr virus
Urine	1ml	62(1) / 150(2) Lassa virus serologically and NAAT positive cases (including different LASV lineages)<br>62(1) / 280(2) Lassa virus serologicall and NAAT negative cases, including cased serologically and/or NAAT positive for any of the following: ebolavirus, marburgvirus, Crimean Congo hemorrhagic fever virus, Rift Valley fever virus, Dengue virus, and Yellow fever virus, or West Nile virus, RSV, measles, mumps, rubella, cytomegalovirus, Epstein Barr virus
Oral fluid	0,5ml	62(1) / 150(2) Lassa virus serologically and NAAT positive cases (including different LASV lineages)<br>62(1) / 280(2) Lassa virus serologicall and NAAT negative cases, including cased serologically and/or NAAT positive for

		any of the following: ebolavirus, marburgvirus, Crimean Congo hemorrhagic fever virus, Rift Valley fever virus, Dengue virus, and Yellow fever virus, or West Nile virus, RSV, measles, mumps, rubella, cytomegalovirus, Epstein Barr virus
Whole blood or DBS	0,2ml WB or 1 DBS	62[(1)] / 150[(2)] Lassa virus serologically and NAAT positive cases (including different LASV lineages)<br>62[(1)] / 280[(2)] Lassa virus serologicall and NAAT negative cases, including cased serologically and/or NAAT positive for any of the following: ebolavirus, marburgvirus, Crimean Congo hemorrhagic fever virus, Rift Valley fever virus, Dengue virus, and Yellow fever virus, or West Nile virus, RSV, measles, mumps, rubella, cytomegalovirus, Epstein Barr virus

[1] applicable to a context of confirmation of suspected and NAAT unconfirmed Nipah (assumed prevalence inside a highly endemic area of 1% positive screening results, expected sensitivity 98%, expected specificity 98%, desired full width of 95% confidence interval 10%)
[2] applicable to a context of screening/detection of Nipah virus (assumed prevalence inside a highly endemic area 5%, expected sensitivity 95%, expected specificity 90%, desired full width of 95% confidence interval 10%)

Positivity status, as an attribute of the donor/patient, encompasses both serological and NAAT positivity. Positivity for **LASV infection** is defined by positive RT-PCR, or positive nucleoprotein antigen test, or increasing IgM titers, or IgM positive with IgG seroconversion. Positivity for **acute LASV infection** is defined by LASV viremia, assessed by antigen capture immunoassay, nucleic acid detection or virus isolation.

Sample sizes have been estimated according to the Zhou et al. statistical approach, for the diagnostic sensitivity and specificity levels, indicated in the Table 2b footnote, using https://finddx.shinyapps.io/SampleSize at an significance level α=0.05 and for a full width of the 95% confidence interval of the evaluated sensitivity and specificity of 10%.

Complete biospecimen sets, including all biospecimen types, from the same donor are preferred. It is critical that for a validation panel, all biospecimens come from the same collection, to avoid preanalytical bias. Preanalytical bias is unavoidable if using biospecimens from different collections with different or undocumented preanalytical specifications.

Possible retrospective sources of specimens can be found in published literature and in completed or ongoing clinical trials (Annex 1). The most suitable sources for identification and validation of stage-specific or treatment efficacy biomarkers would be **longitudinal** collections from geographically different endemic areas, with pre-and post-treatment sampling of patients.

Although the first priority is the needs for development, then for validation of new methods, in order for a new method to be deployed in the field, an external quality assurance (EQA) program is needed, as well as quality control (QC) materials to be included in the kits.

EQA needs

The needs for EQA panels for nucleic acid-based assays are listed below. Table 3 shows the minimum necessary quantities per panel for **one EQA scheme and round** for **40 participating laboratories**, with each laboratory receiving 1 DBS. The quantities correspond to the quantities to be distributed to the participating laboratories, supplemented by the quantities necessary to perform homogeneity and stability testing, and value assignment by the EQA provider.

Concerning non-nucleic acid-based assays (i.e. serological assays), it appears unrealistic for an EQA provider to be able to supply sufficient quantities of antibody positive serum.

Table 3

	**Sample type**	**Quantity per item (for 1 EQA round)**	**Number of items**
Nucleic acid based	DBS spiked with heat treated (1hr 60°C) and gamma irradiated (25kGy) LASV or with synthetic LASV RNA	60 spots	4 items LASV positive (for the target assay), including the following strains: Josiah, CSF, AV, Lib-1580/121<br>2 items LASV negative (for the target assay)

References consulted for the *Lassa virus* TSP

https://www.who.int/publications/i/item/how-to-safely-collect-blood-samples-by-phlebotomy-from-patients-suspected-to-be-infected-with-lassa-fever

https://cdn.who.int/media/docs/default-source/documents/emergencies/health-topics---lassa-fever/shipment-of-blood-samples-lassa.pdf?sfvrsn=8b374f30_2&download=true

MD Bowen et al. Genetic diversity among Lassa virus strains. J Virol 2000;74:6992-7004.

DU Ehichioya et al. Phylogeography of Lassa virus in Nigeria. J Virol 2019;93:e00929-19.

P Emmerich et al. Strain-specific antibody response to Lassa virus in the local population in West Africa. J Clin Virol 2008;42:40-44.

V Raabe & J Koehler. Laboratory diagnosis of Lassa Fever. J Clin Microbiol 2017;55:1629-1637.

S Das et al. A multiplex PCR/LDR assay for the simultaneous identification of category A infectious pathogens: agents of viral hemorrhagic fever and variola virus. PLoS One 2015;10:e0138484.

NG Satterly et al. Comparison of MagPix assays and enzyme linked immunosorbent assay for the dection of hemorrhagic fever viruses. J Clin Microbiol 2017;55:68-78.

M Gabriel et al. Development and evaluation of antibody-capture immunoassays for detection of Lassa virus nucleoprotein-specific immunoglobulin M and G. PLoS Negl Trop Dias 2018;12:e0006361.

LT Mazzola & C Kelly-Cirino. Diagnostics for Lassa fever virus: a genetically diverse pathogen found in low resource settings. BMJ Global Health 2019;4:e001116.

JA Blow et al. Virus inactivation by nucleic acid extraction reagents. J Virol Methods 2004;119:195-198.

E Haddock et al. Effective chemical inactivation of Ebola virus. Emerg Infect Dis 2016;22:1292-1294.

SJ Smither et al. Buffer AVL alone does not inactivate Ebola virus in a representative clinical sample type. J Clin Microbiol 2015;53:3148-3154.

SW Mitchell et al. Physicochemical inactivation of Lassa, Ebola, and Marburg viruses and effect on clinical laboratory analyses. J Clin Microbiol 1984;20:486-489.

ML Boisen et al. Field validation of recombinant antigen immunoassays for diagnosis of Lassa fever. Sci Rep 2018;8:5939.

ML Boisen et al. Field evaluation of a Pan-Lassa rapid diagnostic test during the 2018 Nigerian Lassa fever outbreak. Scientific Reports 2020;10:8724.

VG Dedkov et al. Development and evaluation of a one-step quantitative RT-PCR assay for detection of Lassa virus. J Virol Meth 2019;271:113674.

AN Happi et al. Lassa Fever diagnostics: past, present and future. Curr Opin Virol 2019;37:132-138.

XL Luo et al. Comparative evaluation of standard RT-PCR assays and commercial real-time RT-PCR kits for detection of Lassa virus. Microbiology Spectrum 2023;11: e0501122.

S Nikisins et al. International External Quality Assessment study for molecular detection of Lassa virus. PLoS Negl Trop Dis 2015;9:e0003793.

First WHO International Standard for Lassa virus RNA 21/112, https://nibsc.org/documents/ifu/21-112.pdf

First WHO International Reference Panel for Lassa virus RNA 22/108, https://nibsc.org/documents/ifu/22-108.pdf

Collaborative Study for the Establishment of a WHO International Standardand Reference Panel for Lassa virus RNA, https://cdn.who.int/media/docs/default-source/biologicals/bs-documents-(ecbs)/2022-documents/who_bs_2022.2419_lassa_rna.pdf?sfvrsn=8a790e1e_3&download=true

https://www.fda.gov/medical-devices/device-advice-comprehensive-regulatory-assistance/medical-device-databases

https://www.eptis.org/

Acronyms

CSF	Cerebrospinal fluid
DBS	Dry blood spot
ELISA	Enzyme linked immunosorbent assay
ENIVD	European Network for Diagnostics of “Imported” Viral Diseases
EQA	External Quality Assurance
GP	Glycoprotein
GPC	Glycoprotein precursor
IFA	Immunofluorescence assay
LAMP	Loop mediated isothermal amplification
LASV	Lassa virus
MTA	Material transfer agreement
NAAT	Nucleic acid amplification test
NASBA	Nucleic acid sequence based amplification
NP	Nucleoprotein
PBMCs	Peripheral blood mononuclear cells
PCR	Polymerase chain reaction
POC	Point of care
PQA	WHO prequalification assessment
RDT	Rapid diagnostic test
RUO	Research use only
SNP	Single nucleotide polymorphism

QC	Quality control
VHF	Viral hemorrhagic fever

Annex 1

Specifications of potential sources of biospecimens from published studies and from completed or ongoing clinical trials. Included are articles published after 2019 and clinical trials with at least 30 participants and completed after 2010.

Type of collection	Reference	Contact
2019-2023 PUBLICATIONS		
Blood, vaginal swabs, seminal fluid; 57 suspected cases; 29 confirmed cases; Nigeria	OB Salu et al. Monitoring of Lassa virus infection in suspected and confirmed cases in Ondo State, Nigeria. PanAfrican Medical Journal 2020;36:253.	Dr Olumuyiwa Babalola Salu, obsalu@yahoo.com
Diagnostic remnant samples from 434 patients, Nigeria	ML Boisen et al. Field evaluation of a Pan-Lassa rapid diagnostic test during the 2018 Nigerian Lassa fever outbreak. Scientific Reports 2020;10:8724.	Dr Robert Garry, rfgarry@tulane.edu
Serum; 55 positive cases, 37 negative cases; Guinea	VG Dedkov et al. Development and evaluation of a one-step quantitative RT-PCR assay for detection of Lassa virus. J Virol Meth 2019;271:113674.	Dr Vladimir Dedkov, vgdedkov@yandex.ru
Plasma, oral fluid ; 70 positive cases, 70 negative cases ; Sierra Leone	O Akpogheneta et al. Boosting understanding of Lassa Fever virus epidemiology: field testing a novel assay to identify past Lassa Fever virus infection in blood and oral fluids of survivors and unexposed controls in Sierra Leone. PLoS Negl Trop Dis 2021;15:e0009255.	Dr Hilary Bowen, hilary.bowen@lshtm.ac.uk
Plasma; 22 positive cases, 109 contact cases; Nigeria	JO Shaibu et al. Immunological screening of Lassa virus among health workers and contacts of patients of Lassa fever in Ondo state. Immunobiology 2021;226:152076.	Dr Joseph Ojonugwa Shaibu, jesephshaibu2013@gmail.com

FIND Diagnosis for all

Serum/plasma with follow-up samples; 1000 participants from each of the following countries: Benin, Guinea, Liberia, Nigeria (3 sites), Sierra Leone		Dr Suzanne Penfold, Suzanne.penfold@p-95.com
CLINICAL TRIALS		
2000 participants, blood samples, annual follow up for 3 years, Mali	https://clinicaltrials.gov/study/NCT03783143?cond=Lassa%20Virus%20Infection&rank=1	Heinrich U Feldmann, NIAID feldmannh@mail.nih.gov
42 participants, blood, plasma, Nigeria	https://clinicaltrials.gov/study/NCT06222723?cond=Lassa%20Virus%20Infection&rank=7	Mirjam Groger, Bernhard Nocht Institute for Tropical Medicine groger@bnitm.de
150 participants, PAXgene blood, DBS, Nigeria	https://clinicaltrials.gov/study/NCT04285034?cond=Lassa%20Virus%20Infection&rank=8	University of Oxford
1200 participants, leftover blood, urine, milk and any other bodily fluid samples, Nigeria	https://clinicaltrials.gov/study/NCT03655561?cond=Lassa%20Virus%20Infection&page=2&rank=14	Alexandre Duvignaud , Alliance for International Medical Action alex.duvignaud@gmail.com

Nipah virus, TSP Report

Introduction

Nipah virus (NiV) is a bat-borne, negative-stranded RNA Henipavirus, endemic in tropical and sub-tropical areas in Asia, East Africa, Australia and some oceanic islands. The first reported outbreak was in the Sungai Nipah village in Malaysia, and other outbreaks have been reported in Bangladesh, India, Thailand, Vietnam, Madagascar, Cambodia, Singapore, Indonesia, East Timor, Papua New Guinea, South Africa, Ghana since 1999. Fruit bats (*Pteropus* sp) are the natural host of the virus. Infection occurs by ingestion of NiV-contaminated fruits, vegetables, water or raw date palm sap, or by contact with infected pigs, goats, cattle or dogs. Person to person transmission via droplets also occurs. NiV is a BSL-4 and one of the most pathogenic viruses. It targets the lungs, spleen, kidneys and brain, and causes vasculitis, encephalitis and atypical pneumonia, with fatality rates between 40% and 75%. Asymptomatic infections also occur.

Currently there are no available antiviral treatment or vaccine options for NiV disease and the symptoms are rather not specific. Rapid diagnosis, especially point of care tests, is essential for surveillance and cluster investigation to support implementation of measures to contain epidemics.

A Nipah virus virtual biobank is expected to support findability and availability of biospecimen panels for (i) EQA programs (or equivalent inter-laboratory exercises), reference and quality control material production (ii) method validation, including evaluation and/or PQA, and (iii) R&D other than validation of the analytical and clinical performance characteristics of a diagnostic test. Such R&D includes the identification of novel diagnostic biomarkers and/or their preliminary evaluation.

Scope

This document is intended as a general description of the needs of biological materials in terms of Nipah virus panels, and not as a detailed specification in the format it would be expected to take in an MTA.

The scope of this document includes the needs of biospecimens for development, then validation of

- molecular biology assays, based on detection or measurement of nucleic acids, found in swabs or biological fluids (blood, urine, saliva, CSF)
- assays based on detection or measurement of analytes, other than nucleic acids, found in biological fluids (serum, plasma, whole blood, CSF). Assays intended to be used both in a context of screening and diagnosing Nipah virus infections are in the scope.

The following are out of the scope of this document: immunohistochemistry on tissue samples, virus isolation, electron microscopy and serum neutralization tests with native NiV.

Sources

The content of this report is based on information found in the **References**.

Clinical and biological diagnosis of Nipah virus

Biological diagnosis is critical since clinical symptoms are not specific. Overall, the gold standard for the biological diagnosis of Nipah virus is based on direct detection of the virus or its RNA, or on serum neutralization as serological confirmatory test. The context for Nipah virus diagnosis can be either surveillance or diagnosis of acute infection. The latter can be screening or confirmatory diagnosis.

Because virus isolation and serum neutralization tests require BSL-4 facilities, these are very difficult to deploy. Virus inactivation by sample irradiation at 24 kGy (Hume, 2016), by heat inactivation at 56 °C for 30 min following a 1:5 dilution in PBS buffer containing 0.5% Tween 20 and 0.5% Triton-X100 (Daniels 2001), by bromoethylamine hydrobromide (Berhane 2006) or by TNA-Cifer Reagent E (Pollak 2023) have been proposed, but these processes make virus isolation in cell culture impossible. The use of pseudo-type viruses has been proposed as a surrogate serum neutralization test that does not require BSL-4 facilities (Kaku, 2009).

In the context of surveillance, blood or capillary blood is used for detection of NiV specific antibodies.

In the context of cluster investigation and screening or confirmatory diagnosis, tests for NiV-specific IgM in blood or CSF are performed. Also, NiV antigen or NiV RNA are tested in swabs, urine, saliva, or CSF. RNA extracted from these types of biospecimens can be useful for development of direct diagnostic assays.

Important biospecimen annotations

- Necessary annotations for Nipah virus: geographical origin, headaches, cough, respiratory distress, altered mental status, hypotonia, gaze palsy, limb weakness, areflexia, segmental myoclonus, coma.
- Useful annotations for Nipah virus: preanalytical data (time and temperature before centrifugation/freezing/stabilization, storage temperature), CBC and aminotransferases results.

Nucleic acid based (molecular biology) assays

RT-PCR, RT-LAMP, nested PCR or sequencing-based assays can be used for direct detection of the viral RNA in blood, urine, saliva, CSF or throat swabs. Specimens should be shipped in refrigerated packaging and frozen if the shipment delay is expected to be more than 48 hours. The preferred target is the N (nucleocapsid protein) gene. A one-step qRT-PCR test has been developed targeting the intergenic region between the F and G genes (Jensen 2018). Unless viral inactivation is performed upon collection, these assays require BSL-4 laboratory facilities.

Critical preanalytical factors, for all types of fluid samples, include time and temperature between collection and start of processing, time and temperature between end of processing and storage, long term storage temperature, and RNA extraction kit or method used. Other critical preanalytical factors are the type of anticoagulant for whole blood and blood derivatives (buffy coat, serum, plasma), the centrifugation conditions and use of stabilizer for urine.

Non nucleic acid based (serological or cellular) assays

The Nucleocapsid protein N is the most abundant protein of the virus, it is immunogenic and relatively conserved, and different antibody or antigen capture ELISA systems have been developed for serological diagnosis. Indirect ELISAs using viral antigens for serological testing are preferably based on recombinant rather than native proteins.

Serological assays for IgM in serum or in CSF can be used for diagnosis. In this context, blood samples can be collected as early as one day after the onset of symptoms for testing for IgM. IgM antibodies persist for around 3 months.

Serological assays for IgG do not discriminate active from past infection since antibodies may persist for years after infection. These can be however used for surveillance purposes. IgG antibodies generally appear two weeks after the onset of symptoms.

For antibody-based assays, critical preanalytical factors include the long-term storage conditions. For antigen-based assays, critical preanalytical factors include the type of anticoagulant, the time and temperature between collection and start of processing or analysis, the centrifugation conditions, the time and temperature between end of processing and cryopreservation, the long-term storage conditions.

Potential cross reactivities (important to inform the needs for validation of specificity, as relevant, depending on the analyte):

Significant homologies exist between Nipah virus and Hendra virus genes coding for the N, P, C, V, M, F and G proteins of the viruses, 70%-90% homologies at the genetic level, and 68%-92% homologies at the amino-acid level. Hence, cross reactions can occur in both NAAT and immunoenzymatic assays.

Antibody cross reactivity against Hendra virus (HeV) should be tested. Antibody cross reactivity against different flaviviruses that cause similar symptoms, such as Dengue virus,

West Nile virus, Japanese encephalitis virus, and Zika virus should be tested, as well as against Chikungunya virus, Lassa virus and Respiratory Syncytial virus. Cross reactivity against measles, mumps, rubella, cytomegalovirus, Epstein Barr virus, CCHF, KFD can also be tested.

Table 1 gives the different contexts of use of diagnostic tests and the corresponding most important sample types and method types.

Table 1

Context of use	Sample type and method
Surveillance for Nipah virus	Serum/plasma, DBS (IgG/IgM ELISA or other biomarkers)
Screening for Nipah virus	Serum/plasma, DBS (IgG/IgM ELISA or other biomarkers)<br>DBS, urine, saliva, throat swab (NAAT or other biomarkers)
Confirmatory diagnosis of Nipah virus, confirmation of clinical cases	DBS, CSF, urine, saliva, throat swab (NAAT or other biomarkers)

Need for samples corresponding to different Nipah virus strains:

The main relevant geographical area for NiV biospecimen collection is South and Southeast Asia. Different strains of the virus circulate in different areas or may even coexist in the same area (Angeletti, 2016). A phylogenetic scheme has been proposed based on the nucleocapsid gene sequences, indicating two main lineages, the NiV-Bangladesh-India (NiV-B) and the NiV-

Malaysia-Singapore (NiV-M), with 91.8% genomic sequence similarity (Lo Presti, 2016). Hence, collection of samples from different geographical endemic areas is preferable.

Reference methods:

- NiV isolation on Vero cell line, or sequencing.

Reference materials

RNA, antigens and antibody standards are needed, to be able to compare the performance characteristics of commercially available kits or laboratory developed methods.

The CDC and the Australian Center for Disease Preparedness provide infected cell lysates containing NiV antigens.

Recently, the WHO released a freeze-dried pool of serum samples from 36 convalescent individuals from Bangladesh and Malaysia as reference sample NV1, NIBSC code 22/130. This is the First WHO IS for anti-Nipah virus antibodies for neutralisation assay (22/130nt) with an assigned arbitrary unitage of 250IU/ampoule and the first WHO IS for anti-Nipah virus antibodies for binding assays against the glycoprotein (22/130bd) with an assigned arbitrary unitage of 250IU/ampoule.

FDA reference panels

No FDA reference panel for Nipah virus could be found.

Validated (FDA approved) methods

No FDA approved diagnostic method could be found.

Other methods that have been described/made available

- Very few PCR kits are commercially available for use with human samples
  - Altona Diagnostics NiV & HeV RT-PCR kit (RUO)
  - Biopremier Real Time PCR detection kit Nipah virus (RUO)
  - YOUSEQ Nipah virus qPCR test kit (RUO)
  - Bioperfectus Nipah virus real time PCR kit (CE IVD)
  - Liferiver (Life Technologies) Nipah virus real time RT-PCR kit (CE IVD)
  - MolBio Diagnostics, Truenat RT PCR for Nipah virus (EUA in India)
- Very few ELISA kits are commercially available
  -

- Alpha Diagnostic International (Clinisciences), Human anti-Nipah virus glycoprotein IgG/IgM ELISA kit

No information on the composition of the panels that have been used for method validation by the companies could be found for any of these tests.

- Laboratory developed NAAT and serological tests have been reviewed in Mazzola & Kelly-Cirino 2019. Among these, a multiplex immunoassay on Luminex technology, differentiating between NiV and HeV antibodies has been developed (Bossart 2007). Two multiplex Taqman array cards have been described, one for CSF and one for blood samples and febrile syndromes (Onyango, 2017; Liu, 2016). An RT-LAMP has recently been described that detects all NiV strains (Ma, 2019).

No commercial RDT has been developed so far for NiV infection. A laboratory developed LFI RDT, based on a capture antigen that is common for NiV and HeV and detection monoclonal antibodies, of which one is NiV-specific, has recently been published (Yang, 2022). Another recently developed laboratory method is based on isothermal amplification and lateral flow detection, without requirement for an RNA extraction step (Pollak, 2023).

<u>EQA programs</u>

No NiV EQA program could be found on the EPTIS website.

One EQA scheme including NiV has been organized in China, based on spiked artificial CSF samples (Zhang, 2021).

Panel needs

R&D / method validation needs

Table 2a shows the needs in terms of panels for initial development /feasibility studies, for both nucleic acid-based and non-nucleic acid-based assays.

Table 2a

Sample type	Quantity per donor (for 1 development panel)	Number of biospecimen donors
Serum/ plasma	1ml	20 Nipah virus serologically and NAAT positive cases<br>20 Nipah virus serologically and NAAT negative cases
Urine	2ml	20 Nipah virus serologically and NAAT positive cases<br>20 Nipah virus serologically and NAAT negative cases
Saliva	1ml	20 Nipah virus serologically and NAAT positive cases<br>20 Nipah virus serologically and NAAT negative cases
Whole blood or DBS	0,4ml WB or 2 DBS	20 Nipah virus serologically and NAAT positive cases<br>20 Nipah virus serologically and NAAT negative cases
CSF*	0,5ml	20 Nipah virus serologically and NAAT positive cases<br>20 Nipah virus serologically and NAAT negative cases

*not the preferred sample type

Table 2b shows the needs in terms of complete validation panels for each indication and for both nucleic acid-based and non-nucleic acid-based assays. Numbers of donors are defined based on the draft WHO TPP document.

Table 2b

Sample type	Quantity per donor (for 1 validation panel)	Number of biospecimen donors
Serum/ plasma	0,5ml	62[(1)] / 150[(2)] Nipah virus serologically and NAAT positive cases (ideally including both NiV-B and NiV-M)<br>62[(1)] / 280[(2)] Nipah virus serologicall and NAAT negative cases, including cased serologically and/or NAAT positive for Hendra virus (HeV), and any of the following: Dengue virus, West Nile virus, Japanese encephalitis virus, Zika virus, Chikungunya virus, Lassa virus, RSV, measles, mumps, rubella, cytomegalovirus, Epstein Barr virus, CCHF, KFD
Urine	1ml	62[(1)] / 150[(2)] Nipah virus serologically and NAAT positive cases (ideally including both NiV-B and NiV-M)<br>62[(1)] / 280[(2)] Nipah virus serologicall and NAAT negative cases, including cased serologically and/or NAAT positive for Hendra virus (HeV), and any of the following: Dengue virus, West Nile virus, Japanese encephalitis virus, Zika virus, Chikungunya virus, Lassa virus, RSV, measles, mumps, rubella, cytomegalovirus, Epstein Barr virus, CCHF, KFD
Saliva	0,5ml	62[(1)] / 150[(2)] Nipah virus serologically and NAAT positive cases (ideally including both NiV-B and NiV-M)<br>62[(1)] / 280[(2)] Nipah virus serologicall and NAAT negative cases, including cased serologically and/or NAAT positive for Hendra virus (HeV), and any of the following: Dengue virus, West Nile virus, Japanese encephalitis virus, Zika virus, Chikungunya virus, Lassa virus, RSV, measles, mumps, rubella, cytomegalovirus, Epstein Barr virus, CCHF, KFD
Whole blood or DBS	0,2ml WB or 1 DBS	62[(1)] / 150[(2)] Nipah virus serologically and NAAT positive cases (ideally including both NiV-B and NiV-M)<br>62[(1)] / 280[(2)] Nipah virus serologicall and NAAT negative cases, including cased serologically and/or NAAT positive for Hendra virus (HeV), and any of the following: Dengue virus, West Nile virus, Japanese encephalitis virus, Zika virus,

		Chikungunya virus, Lassa virus, RSV, measles, mumps, rubella, cytomegalovirus, Epstein Barr virus, CCHF, KFD
CSF*	0,1ml	62(1) / 150(2) Nipah virus serologically and NAAT positive cases (ideally including both NiV-B and NiV-M)<br>62(1) / 280(2) Nipah virus serologicall and NAAT negative cases, including cased serologically and/or NAAT positive for Hendra virus (HeV), and any of the following: Dengue virus, West Nile virus, Japanese encephalitis virus, Zika virus, Chikungunya virus, Lassa virus, RSV, measles, mumps, rubella, cytomegalovirus, Epstein Barr virus, CCHF, KFD

[1] applicable to a context of confirmation of suspected and NAAT unconfirmed Nipah (assumed prevalence inside a highly endemic area of 1% positive screening results, expected sensitivity 98%, expected specificity 98%, desired full width of 95% confidence interval 10%)
[2] applicable to a context of screening/detection of Nipah virus (assumed prevalence inside a highly endemic area 5%, expected sensitivity 95%, expected specificity 90%, desired full width of 95% confidence interval 10%)
*not the preferred sample type

Positivity status, as an attribute of the donor/patient, encompasses both serological and NAAT positivity. Positivity for Nipah virus infection is defined as a suspected case who has laboratory confirmation of NiV infection either by (i) positive MiV RNA PCR in respiratory secretions, urine or CSF, (ii) isolation of NiV from respiratory secretions, urine or CSF (Aditi & Shariff, 2019).

Sample sizes have been estimated according to the Zhou et al. statistical approach, for the TPP-specified diagnostic sensitivity and specificity levels, using https://finddx.shinyapps.io/SampleSize at a significance level α=0.05 and for a full width of the 95% confidence interval of the evaluated sensitivity and specificity of 10%.

Complete biospecimen sets, including all biospecimen types, from the same donor are preferred. It is preferable that for a validation panel, all biospecimens come from the same collection, to avoid preanalytical bias. Preanalytical bias is unavoidable if using biospecimens from different collections with different or undocumented preanalytical specifications. However, given the rarity of the NiV infections, positive specimens have to be collected in the context of outbreaks, wherever these occur. Possible retrospective sources of specimens might be found in published literature, as the example in Table 3. No completed or ongoing clinical trials could be found that would be collecting biospecimens.

Table 3

Type of collection	Reference	Contact
18 IgM positive, 310 IgG positive, 279 negative serum samples, India	AM Shete et al. Indian J Med Res 2022;156:429-434	Dr D Pragya, Indian Council of Medical Research, National Institute of Virology, hellopragya22@gmail.com

Although the first priority is the needs for development, then for validation of new methods, in order for a new method to be deployed in the field, an external quality assurance (EQA) program is needed.

EQA needs

The needs for EQA panels for nucleic acid-based assays are listed below. Table 4 shows the minimum necessary quantities per panel for **one EQA scheme and round** for **40 participating laboratories**, with each laboratory receiving 1 DBS. The quantities correspond to the quantities to be distributed to the participating laboratories, supplemented by the quantities necessary to perform homogeneity and stability testing, and value assignment by the EQA provider.

Concerning non-nucleic acid-based assays (i.e. serological assays), it appears unrealistic for an EQA provider to be able to supply sufficient quantities of antibody positive serum.

Table 4

	Sample type	Quantity per item (for 1 EQA round)	Number of items
Nucleic acid based	DBS spiked with gamma irradiated NiV or with	60 spots	3 items Nipah positive (for the target assay)<br>1 items Nipah negative (for the target assay)

	synthetic NiV RNA		

References consulted for the Nipah virus TSP

WHO, 2024. Nipah virus infection, https://www.who.int/news-room/fact-sheets/detail/nipah-virus

CDC, 2022. Nipah virus, https://www.cdc.gov/vhf/nipah/index.html

S Angeletti et al. Molecular epidemiology and phylogeny of Nipah virus infection: a mini review. Asian Pacific J Trop Med 2016;9:630-634.

A Lo Presti et al. Origin and evolution of Nipah virus. J Med Virol 2016;88:380-388.

Aditi & M Shariff. Nipah virus infection: a review. Epidemiology and Infection 2019;147:e95.

P Daniels et al. Laboratory diagnosis of Nipah and Hendra virus infections. Microbes Infection 2001;3:289-295.

AJ Hume et al. Inactivation of RNA viruses by gamma irradiation: a study on mitigating factors. Viruses 2016;8:204.

Y Berhane et al. Production and characterization of monoclonal antibodies against binary ethylenimine inactivated Nipah virus. J Virol Methods 2006;132:59-68.

NM Pollak et al. Evaluation of three rapid low-resource molecular tests for Nipah virus. Frontiers in Microbiology 2023; 13:1101914.

Y Kaku et al. A neutralization test for specific detection of Nipah virus antibodies using pseudotyped vascular stomatitis virus expressing green fluorescent protein. J Virol Meth 2009;160:7-13.

N Thakur & D Bailey. Advances in diagnostics, vaccines and therapeutics for Nipah virus. Microbes and Infection 2019;21:278-286.

KS Jensen et al. Development of a novel real-time polymerase chain reaction assay for the quantitative detection of Nipah virus replicative viral RNA. PLoS One 2018;13:e0199534.

LT Mazzola & C Kelly-Cirino. Diagnostics for Nipah virus: a zoonotic pathogen endemic to spoutheast Asia. BMJ Global Health 2019;4:e001118.

KN Bossart et al. Neutralization assays for differential henipavirus serology using Bioplex protein array systems. J Virol Methods 2007;142:29-40.

CO Onyango et al. Evaluation of a TaqMan Array Card for detection of central nervous system infections. J Clin Microbiol 2017;55:2035-2044.

J Liu et al. Development of a TaqMan Array Card for acute febrile illness outbreak investigation and surveillance of emerging pathogens, including Ebola virus. J Clin Microbiol 2016;54:49-58.

L Ma et al. Rapid and specific detection of all known Nipah virus strains' sequences with reverse transcription loop mediated isothermal amplification. Front Microbiol 2019;10:418.

WHO Expert Committee on Biological Standardization, 2023/BS/2023.2458, https://cdn.who.int/media/docs/default-source/biologicals/bs-documents-(ecbs)/2023-bs-documents/who_bs_2023.2458_1st-is-for-anti_nipah.pdf?sfvrsn=3587cbb8_1&download=true

WHO R&D Blueprint: Priority Diagnostics for Nipah Use Cases and Target Product Profiles, 2019, https://cdn.who.int/media/docs/default-source/blue-print/call-for-comments/who-nipah-dx-tpps-d.pdf?sfvrsn=8a856311_4

M Yang et al. Detection of Nipah and Hendra viruses using recombinant human ephrin B2 capture viruses in immunoassays. Viruses 2022;14:1657.

https://www.nibsc.org/

https://www.fda.gov/medical-devices/device-advice-comprehensive-regulatory-assistance/medical-device-databases

https://www.eptis.org/

R Zhang et al. External quality assessment of molecular testing of 9 viral encephalitis-related viruses in China. Virus Research 2021;306:198598.

XH Zhou et al. Statistical Methods in Diagnostic Medicine. 2011;2:193-228.

Acronyms

BSL	Biosafety Level
CCHF	Crimean-Congo Haemorrhagic Fever
CSF	Cerebrospinal fluid
DBS	Dry blood spot
ELISA	Enzyme linked immunosorbent assay
EQA	External Quality Assurance
EUA	Emergency Use Authorization
HeV	Hendra virus
IS	International Standard
KFD	Kyasanur Forest Disease
LAMP	Loop mediated isothermal amplification
LFI	Lateral Flow Immunochromatographic
MTA	Material transfer agreement
NAAT	Nucleic acid amplification test
NASBA	Nucleic acid sequence based amplification
NiV	Nipah virus
PBMCs	Peripheral blood mononuclear cells
PCR	Polymerase chain reaction
POC	Point of care
PQA	WHO prequalification assessment
RDT	Rapid diagnostic test

RSV	Respiratory Syncytial virus
RUO	Research Use Only
SNP	Single nucleotide polymorphism
QC	Quality control
